# Tuneable thiol exchange linkers for traceless drug release applications in prodrugs and ADCs†

**DOI:** 10.1039/d4cc01558d

**Published:** 2024-06-12

**Authors:** Raoul Walther, Mahri Park, Nicola Ashman, Martin Welch, Jason S. Carroll, David R. Spring

**Affiliations:** a Yusuf Hamied Department of Chemistry, University of Cambridge Lensfield Road CB2 1EW Cambridge UK; b Department of Biochemistry, University of Cambridge CB2 1QW Cambridge UK; c Cancer Research UK Cambridge Institute, Robinson Way CB2 0RE Cambridge UK

## Abstract

We describe a versatile and tuneable thiol responsive linker system using thiovinylketones, which relies on the conjugate addition–elimination mechanism of Michael acceptors for the traceless release of therapeutics. In a proof-of-principle study, we translate our findings to exhibit potent thiol-cleavable antibiotic prodrugs and antibody–drug conjugates.

## Abstract 2

We describe a versatile and tuneable thiol responsive linker system using thiovinylketones, that relies on the conjugate addition–elimination mechanism of Michael acceptors for the traceless release of therapeutics.

## Graphical abstract

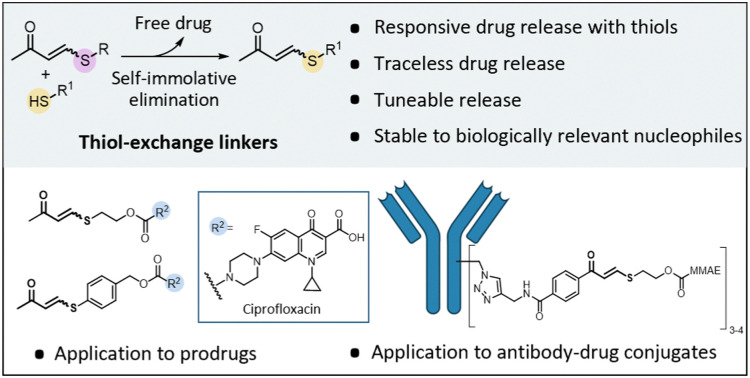

Traceless drug release is an important characteristic for the development of efficacious prodrugs and antibody–drug conjugates (ADCs). For prodrugs, the therapeutic is chemically modified to contain a promoiety, and for ADCs, a linker is used to connect a therapeutic antibody to reduce systemic toxicity and off-target activity. Bioactivity of the drug may be restored by the incorporation of a stimuli responsive linker, which responds to a physiological stimulus, such as differing redox environments, overexpression of enzymes, or changes in pH.^1,2^

Electrophilic warheads can be found in natural products, such as beta lactam antibiotics, mitomycin C, and duocarmycin, featuring the functional groups: beta-lactam, aziridine, and cyclopropane, respectively. Remarkably, many electrophile-bearing natural products are rather selective for a specific target in the cell.^3–6^ For example, electrophiles, such as cyclopropene, Michael acceptors, and bicyclo[1.1.0]butane carboxylic amide, have shown highly selective protein labelling in live cells.^7–10^ Similarly, ligand-directed protein labelling strategies further highlight the capacity to tune highly reactive functional groups for targeted and site-specific protein labelling in a cellular environment.^11,12^

The promising literature precedence encouraged us to explore electrophiles and covalent dynamic chemistry. We speculated that it should be possible to exploit the reversibility and addition–elimination mechanism of conjugate additions and translate this property to chemically triggered, traceless drug release. We were particularly interested in capitalising on the intrinsic redox discrepancies between the extracellular- and intracellular environments stemming from the low micromolar and high millimolar concentration of glutathione (GSH) outside and inside of the cell, respectively.^13^

So far, only a handful of examples utilise the addition–elimination mechanism of Michael acceptors, derived from Meldrum's acid, alpha-substituted methacrylates, and enaminone to generate stimuli responsive materials and chemical biology tools.^11,14–19^ A recent review gives a timely overview of the subject.^20^ One covalent dynamic system of particular interest to us was the addition of thiols to electron poor alkynones resulting in thiovinylketones (TVK) (Fig. 1a).^21^

**Fig. 1:**
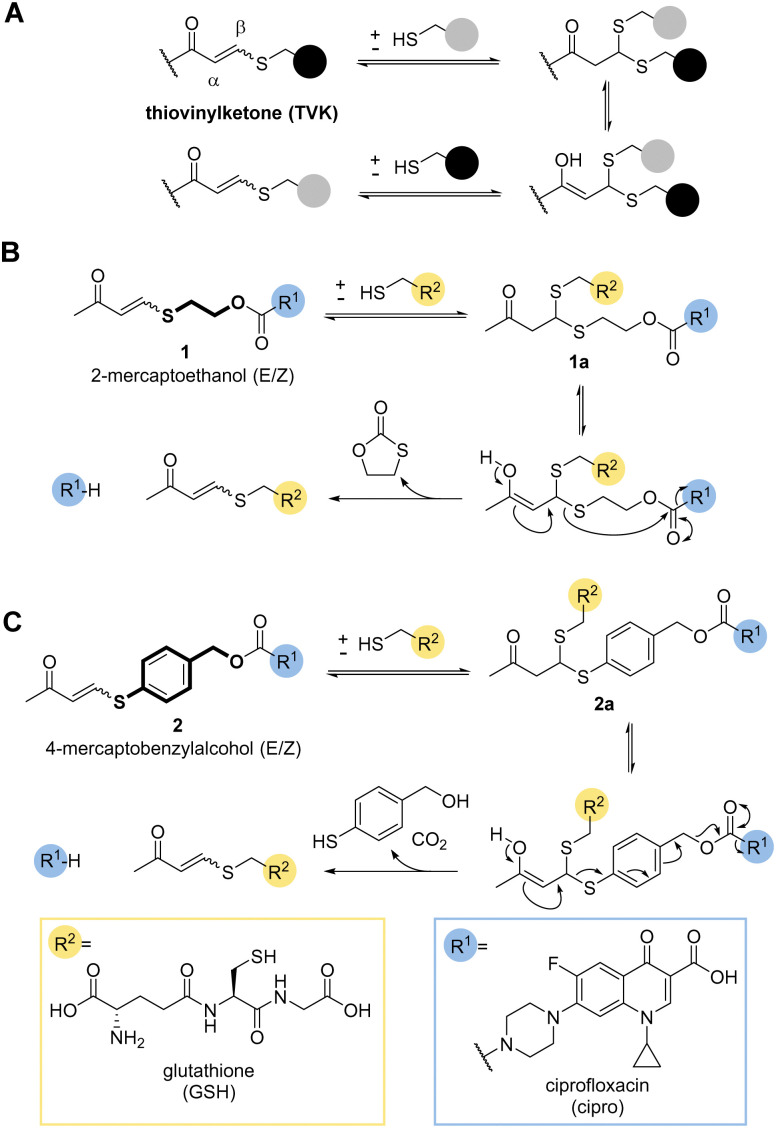
(A) Thiovinylketone (TVK) functional group and its addition–elimination reaction with thiols. (B) and (C) Proposed design of self-immolative linker containing TVK based on cyclisation (2-mercaptoethanol, B) and elimination (4-mercaptobenzyl alcohol, C) for traceless drug release.

Given its dynamic nature,^22^ thiol Michael addition to TVK produces thioacetals which are in constant exchange and can eliminate a thiol by retro-Michael reaction, *i.e.* undergo vinylic substitution (Fig. 1a). We anticipated that the cysteine thiol of GSH would act as a nucleophile and initiate the release of the chosen cargo. Prominent marketed examples of therapeutics, which rely on this endogenous targeting opportunity are antibody drug conjugates (ADCs), Mylotarg® and Besponsa®. In both cases, reducible disulfide bonds,^23–25^ are used for drug release. We postulated that we could use the addition–elimination mechanism of conjugate additions to TVK and push the equilibrium towards the product by the introduction of an irreversible reaction step. In this regard, we propose the use of self-immolative linkers, such as 2-mercaptoethanol (Fig. 1b) or 4-mercaptobenzylalcohol (Fig. 1c) for traceless drug release.

Herein, we present an analysis of the reactivity and stability of the designed linker motif against a panel of nucleophiles. We demonstrate that the electronic and steric design of the linker allows fine-tuning of the release kinetics. Lastly, we illustrate its applicability and feasibility in two biological settings, namely in the design of thiol responsive antibiotic prodrugs and a thiol- responsive ADC.

Initial work focused on model linker systems 1 and 2 (Fig. 1b and c, respectively), bearing the antibiotic ciprofloxacin (see ESI,† for detailed synthetic access, Scheme S1A). 1 consists of the cyclisation-based linker 2-mercaptoethanol, whereas 2 incorporates the elimination-based linker 4-mercaptobenzyl alcohol. Nucleophilic addition of a thiol, such as GSH, will generate the thioacetal species 1a and 2a. The retro-Michael reaction either liberates the 2-mercaptoethanol or 4-mercaptobenzylalcohol based linker and the release of the drug becomes irreversible (Fig. 1b and c). In the case of 2-mercaptoethanol, the thiol is removed from the equilibrium due to 5-*exo-trig* cyclisation. In the case of 4-mercaptobenzyl alcohol, expulsion of carbon dioxide makes the elimination of the cargo irreversible.

To investigate our hypothesis, the model antibiotic prodrugs 1 and 2 were first subjected to a range of thiols, including physiologically relevant thiols GSH and l-cysteine, in phosphate buffer at three pH values (6.8, 7.4, and 8.0); Fig. 2a and Fig. S1, S2 (ESI†). Pleasingly, in the absence of thiol, the drug-linker systems showed no evident release of the drug, ciprofloxacin, over a period of 24 h at 37 °C. At pH 6.8 and pH 8.0, linkers 1 and 2, respectively, showed decreased AUC which we attribute to compound precipitation, given that no release of the apparent drug was observed (Fig. 2a and Fig. S3, S4, ESI†).

**Fig. 2:**
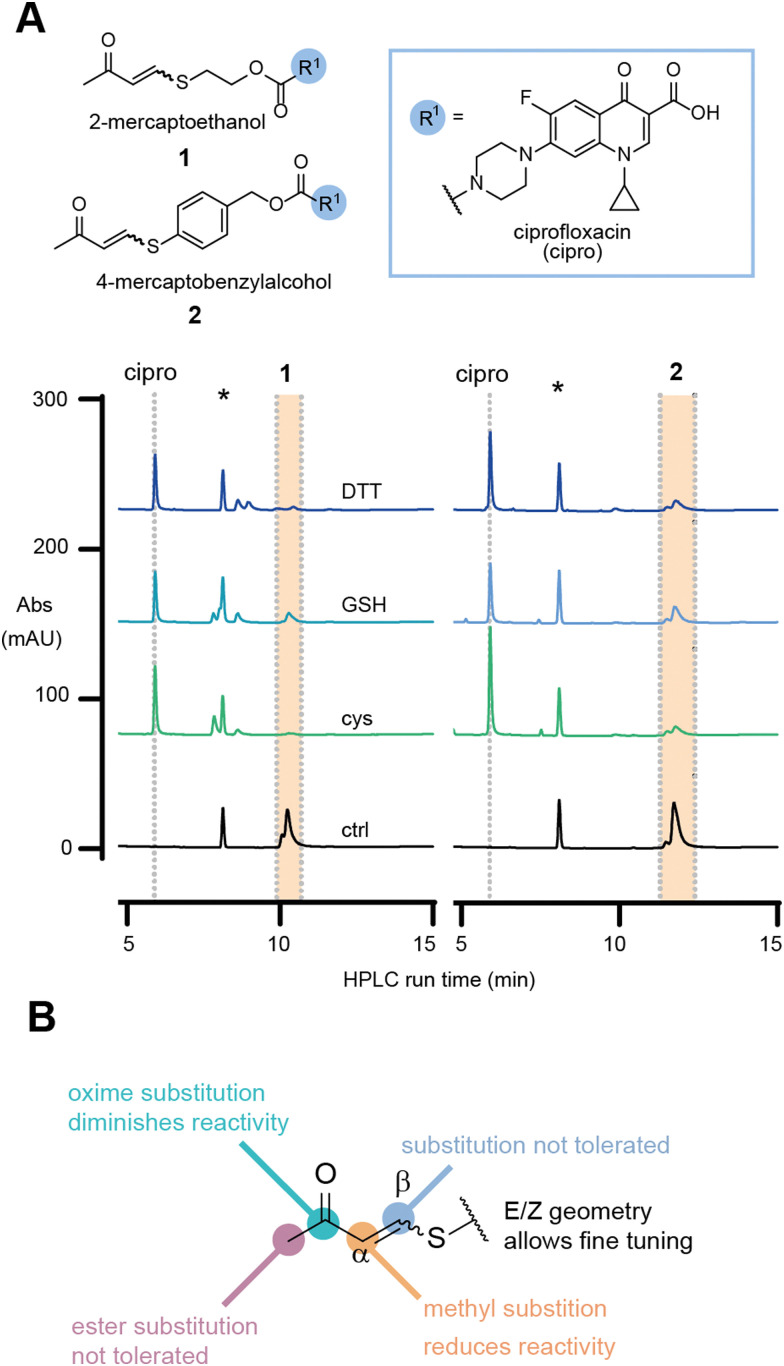
(A) Release studies of 1 and 2 in the presence of different thiol species at superstoichiometric concentrations (5 mM) in phosphate buffer, pH 7.4 at 37 °C after 60 min. ctrl = compounds in the absence of thiol; cys = l-cysteine; GSH = glutathione; DTT = dl-dithiothreitol; * marks internal standard. (B) Investigation of substitution pattern on TVK and its observed consequence on thiol responsiveness.

In the presence of a physiologically relevant intracellular thiol concentration (5 mM), both linker systems showed quantitative consumption of the starting material within 1 h at all pH values for 1, and for 2 at pH 7.4 and above (Fig. 2a and Fig. S1, S2, ESI†).

To rule out cross reactivity with other nucleophiles, such as lysine, we incubated 1 and 2 with a model amine, l-lysine methyl ester (l-Lys-OMe), and a peptide containing other biologically relevant nucleophiles, Ac-CRLYGFKC-NH_2_. For the reaction with l-Lys-OMe, no release was observed during the 24 h incubation (Fig. S5 and S7, ESI†). For the reaction with the peptide, release was observed only when cysteine thiols were available (Fig. S6, ESI†). No release, nor addition products, were observed when cysteine side chains were alkylated with iodoacetamide (Fig. S6 and S7, ESI†). This experiment illustrates that the linker is stable towards arginine, amine and phenol nucleophiles.

In the initial evaluation of TVK as a thiol responsive linker, the stereochemical geometry of the linker was neglected. Nevertheless, the initial experiments using an *E*/*Z* mixture of 1 and 2 indicated that both stereochemical geometries, *E* and *Z*, will undergo conjugate addition with thiols (at superstoichiometric concentrations – 5 mM) and release the model drug cargo. With our greater interest in antibody–drug conjugates and the understanding that the incorporation of highly hydrophobic linkers and drugs onto antibodies remains a challenge, we focused on the characterisation of linkers based on 1. Separation of the diastereomers of 1 and HPLC analysis of the single isomeric species in the same HPLC assay revealed that the *E*-isomer is more reactive towards GSH, with a half-life of 68 min for the consumption of the starting material, whereas a half-life of >1500 min was determined for the *Z*-isomer (Fig. S8, ESI†).

Collectively, these experiments revealed no cross reactivity of the linker against other biological nucleophiles and demonstrates the versatility of TVK as a chemically triggerable linker, where the release may be tuned by the stereochemical geometry. Further evaluation of the linker substitution pattern (see ESI,† for experimental details and Scheme S1B, C) indicated that substitution in the beta position and alteration of the electron withdrawing group to an ester were not tolerated (Fig. 2b and Table S1, ESI†). Introduction of a methyl group in the alpha position was tolerated, although resulted in slower release of the functional cargo. This may be suitable for the development of prodrugs or smart materials capable of sustained drug release^26^ or low reactivity activity-based protein profiling applications.^27^ Converting the carbonyl of 1 into an oxime resulted in a stable analogue that was inert to reactions with thiols. As such, the oxime analogue serves as a negative control for later evaluation.

Having established a fundamental understanding of TVK in a model system, we set out to answer how TVK may perform in a biological context, namely for the design of thiol-triggerable prodrugs and antibody–drug conjugates (ADCs). To this end, we determined the efficacy of 1 and 2 (mixture of isomers) in inhibiting the growth of bacteria by assessing the minimum inhibitory concentration (MIC). We anticipated that blocking the secondary amine of ciprofloxacin as a carbamate should result in a decreased potency due to a net negative charge of the resulting prodrugs.

Indeed, MIC experiments against two selected Gram-negative bacterial strains, *Pseudomonas aeruginosa* PA01 and PA14, revealed a drop in potency for 1 and 2 as opposed to the parent antibiotic (Fig. 3a and Fig. S9, ESI†). In the presence of a physiologically relevant concentration of GSH (5 mM), the potency of 1 and 2 increased, with 2 exhibiting essentially the same potency as ciprofloxacin (Fig. 3a).

**Fig. 3:**
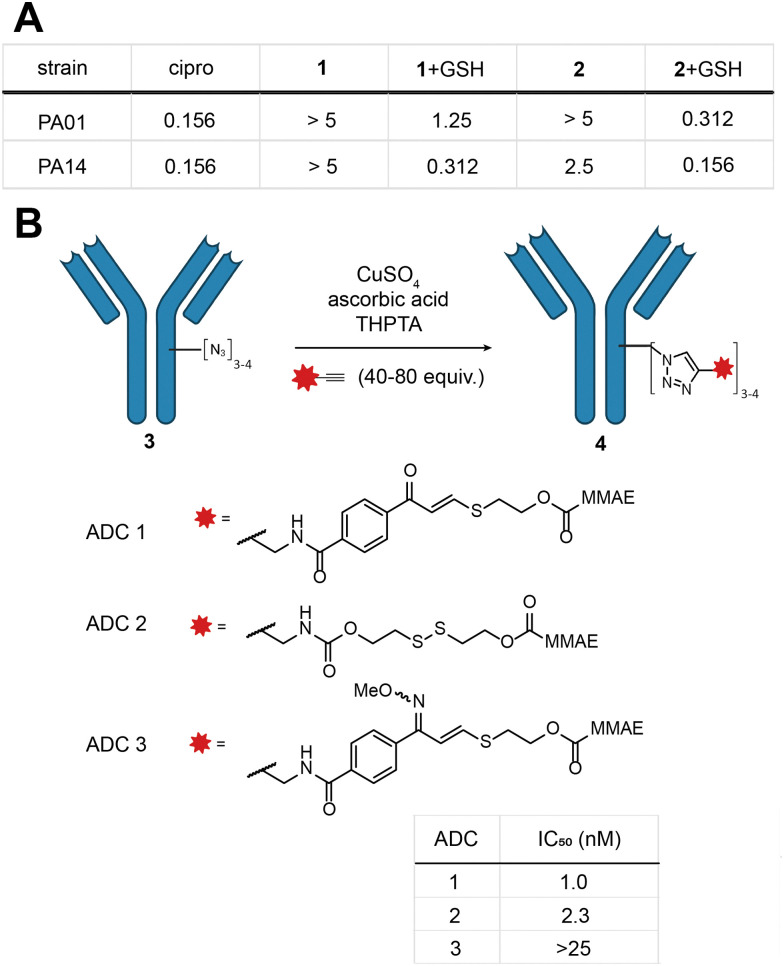
(A) Summary table of MIC (concentration in μM) of 1 and 2 (mixture of isomers) in the presence and absence of 5 mM GSH; (B) *in vitro* cytotoxicity of ADCs 1–3 was measured against the HER2-positive breast cancer cell line SKBR3 and IC_50_ values are given.

With these promising results in hand, we turned our attention to the application of the *E*-isomer of linker 1 towards ADCs (see ESI,† for full synthetic details Scheme S2, S3 and ADC characterisation). We opted for a two-step approach to generate the ADC: first installation of a bioorthogonal chemical handle by the established rebridging technology developed in our laboratory (Fig. 3b),^28^ followed by the incorporation of the TVK cleavable motif through copper-catalysed click chemistry containing the cytotoxin monomethylauristatin E (MMAE) (Fig. 3a). Three ADCs (ADC 1–3) were selected for synthesis, each comprising trastuzumab, a HER2 targeting therapeutic antibody. ADC 1 contains the chemically cleavable TVK motif based on 2-mercaptoethanol. ADC 2 contains an established thiol cleavable disulfide motif, to allow comparison with our novel linker. Lastly, we synthesised a control ADC where the carbonyl motif of ADC 1 was masked by an oxime (ADC 3) to precisely demonstrate the thiol reactivity. The stability of the oxime in the presence of thiols was previously confirmed in model linker studies. With the three ADCs in hand, their *in vitro* cytotoxicity against HER2-positive breast cancer cells (SKBR3) was examined. ADC 1 and ADC 2 exhibited potent dose-dependent cytotoxicity, with IC_50_ values of 1.0 and 2.3 nM, respectively (Fig. 3c and Fig. S10, ESI†). Masking the carbonyl of ADC 1 as an oxime (ADC 3) markedly reduced the potency in the investigated concentration range (IC_50_ > 25 nM). Collectively, these results illustrate the successful incorporation of TVK as a cleavable thiol-responsive linker in ADC design. ADC 3 further supports the releasable linker strategy and underscores the importance of the electrophilic warhead in releasing the cargo.

In summary, we have demonstrated that our TVK-containing thiol triggerable linkers are shown to be cleavable by physiologically relevant thiols and stable towards several biologically relevant nucleophilic residues, such as lysine. We showed that stereochemical geometry of the linker may allow tuneable drug release since the *E*-isomer of the linker is more reactive towards GSH than the *Z*-isomer. Prodrugs of the antibiotic ciprofloxacin were generated and shown to be potent in the presence of physiologically relevant concentrations of GSH. Finally, thiol responsive anti-HER2 ADCs were successfully generated and displayed potent cytotoxicity *in vitro*. We anticipate that these thiol responsive linkers may be a resourceful linker choice for the development of prodrugs and ADCs allowing tuneable, traceless drug release.

RW conceptualised the project. RW, MIP, NA performed experiments and conducted data interpretation. DRS supervised the project. The manuscript was written with input from all authors. MIP and NA each acknowledge a studentship from AstraZeneca. JSC was funded by CRUK core funding. MW acknowledges the Cystic Fibrosis Trust UK. RW acknowledges the Danish Independent Research Fund (0170-00001B). DRS acknowledges support from EPSRC, BBSRC, MRC and Cystic Fibrosis Trust UK. ADC figure created with https://Biorender.com.

## Data availability

The data supporting this article have been included as part of the ESI.†

## Conflicts of interest

There are no conflicts to declare.

## Supplementary Material

CC-060-D4CC01558D-s001
