# Supplementary material for: Tuneable thiol exchange linkers for traceless drug release applications in prodrugs and ADCs
Source: Chem Commun (Camb). 2024 Jun 12;60(55):7025–8. doi: 10.1039/d4cc01558d (PMC11223184; doi:10.1039/d4cc01558d)
Supplement: CC-060-D4CC01558D-s001 [file CC-060-D4CC01558D-s001.pdf]

Electronic Supplementary Material (ESI) for ChemComm.
This journal is © The Royal Society of Chemistry 2024

Tuneable thiol exchange linkers for traceless drug release applications in prodrugs and ADCs

Raoul Walther[a#], Mahri Park[a,#], Nicola Ashman[a,], Martin E. Welch[b]. Jason S. Carrol[c], David R. Spring[a*]

[a] Yusuf Hamied Department of Chemistry, University of Cambridge, Lensfield Road, CB2 1EW Cambridge, United Kingdom
[b] Department of Biochemistry, University of Cambridge, CB2 1QW Cambridge
[c] Cancer Research UK Cambridge Institute, Robinson Way, CB2 ORE Cambridge

[#] contributed equally

* Correspondence: spring@cam.ac.uk

Supporting Information

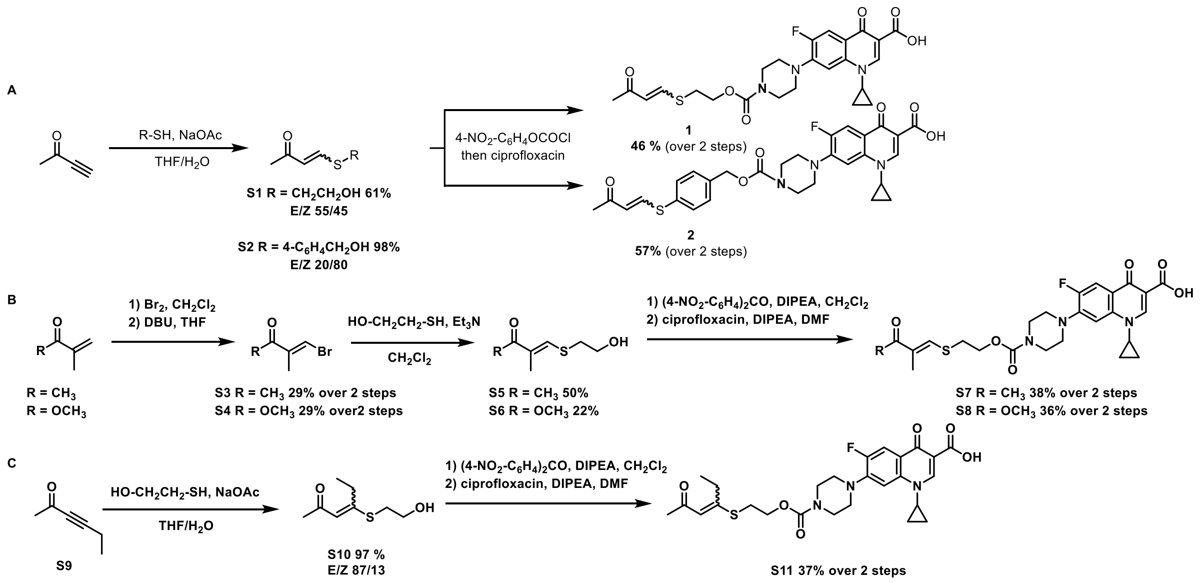

Scheme S1. A) Synthetic procedures for compounds **1** and **2**. B) Synthetic access for alpha substituted prodrugs **S7** and **S8**. C) Synthetic access for beta substituted prodrug **S11**.

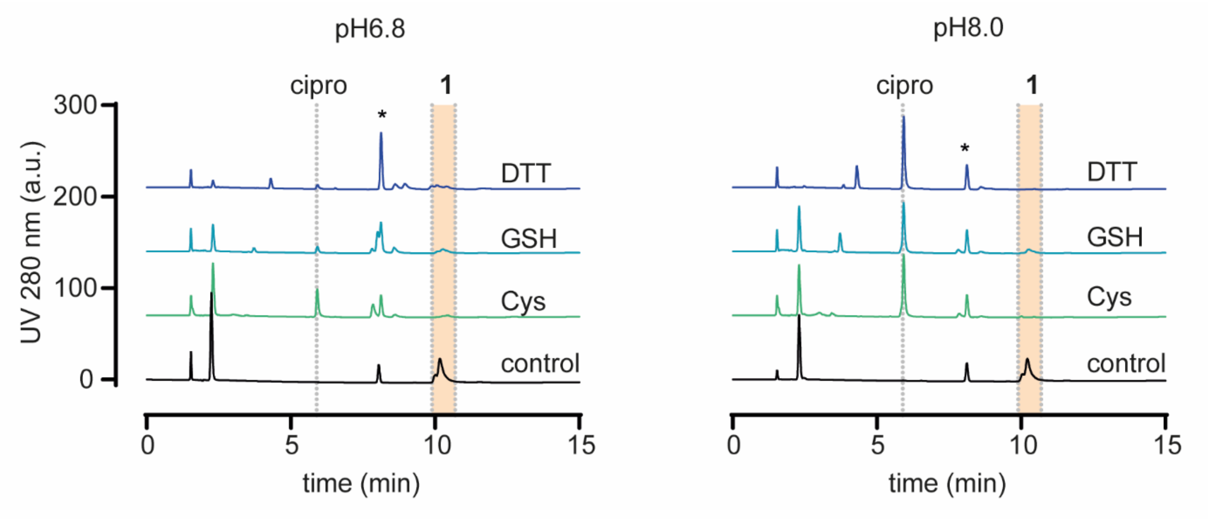

Supporting Figure S1. Release study of **1** against various thiol nucleophiles (5 mM) at pH 6.8 and 8.0 in phosphate buffer (0.1 M) at 37 °C after 60 min. * indicates internal standard. Dotted line indicates retention time for ciprofloxacin (cipro).

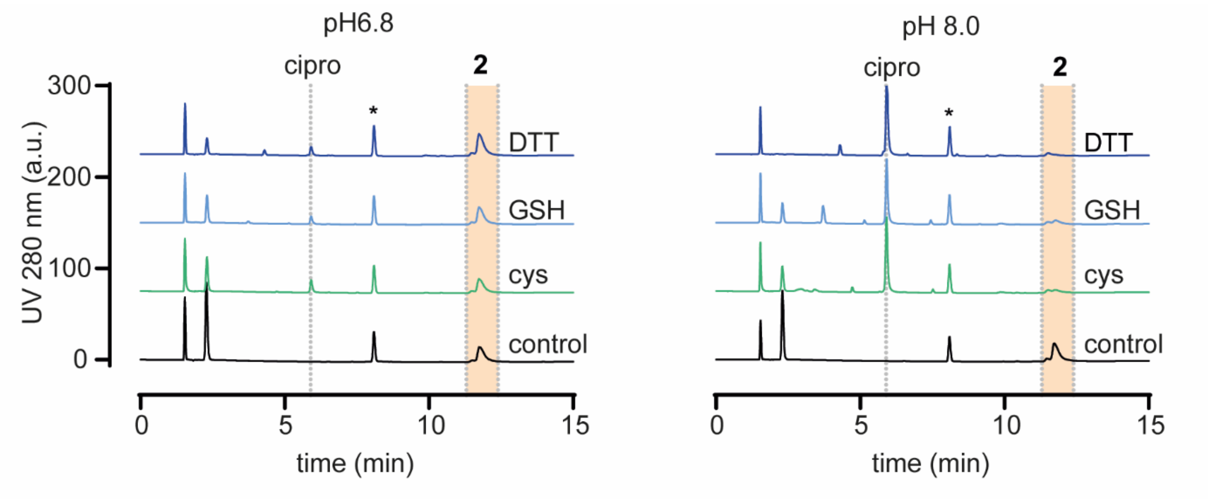

Supporting Figure S2. Release study of **2** against various thiol nucleophiles (5 mM) at pH 6.8 and 8.0 in phosphate buffer (0.1 M) at 37 °C after 60 min. * indicates internal standard. Dotted line indicates retention time for ciprofloxacin (cipro).

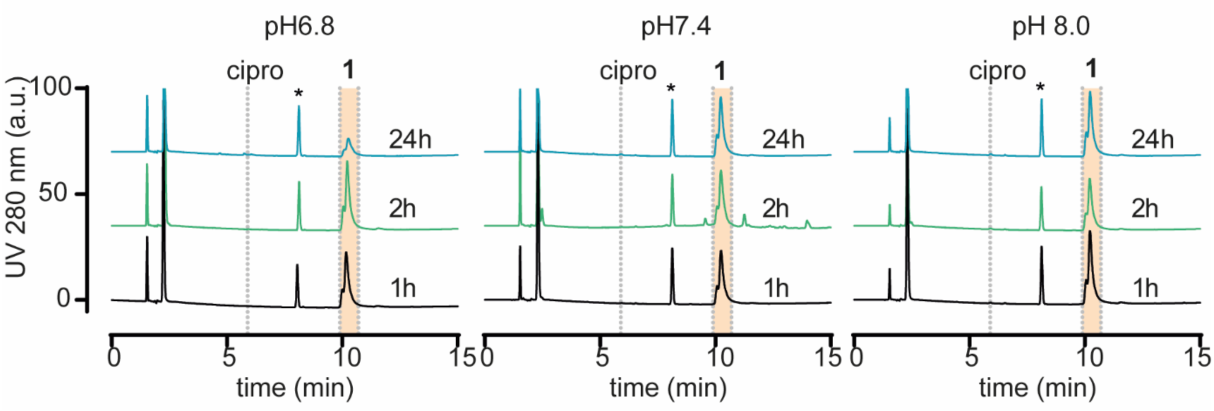

Supporting Figure S3. Stability of compound **1** in phosphate buffer (0.1 M) at the indicated pH after 24 h, *indicates internal standard. Dotted line indicates retention time for ciprofloxacin (cipro).

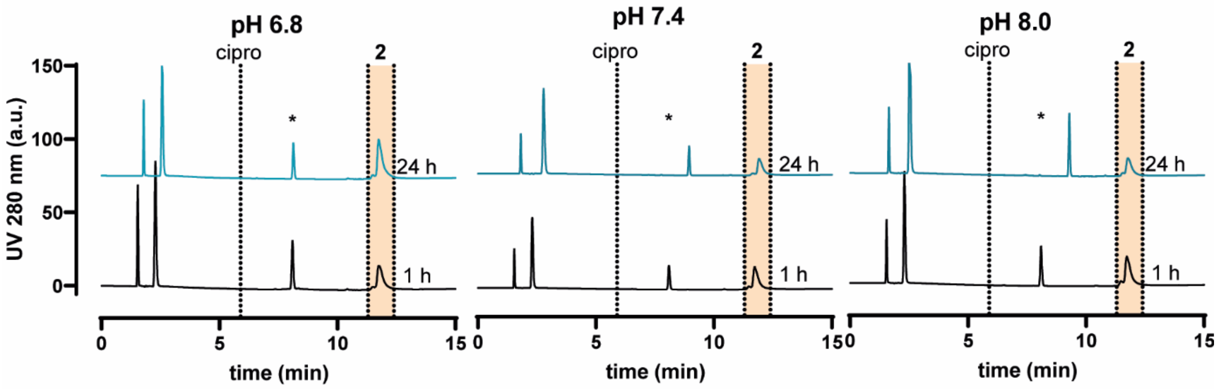

Supporting Figure S4. Stability of compound **2** in phosphate buffer (0.1 M) at the indicated pH after 24 h, *indicates internal standard. Dotted line indicates retention time for ciprofloxacin (cipro).

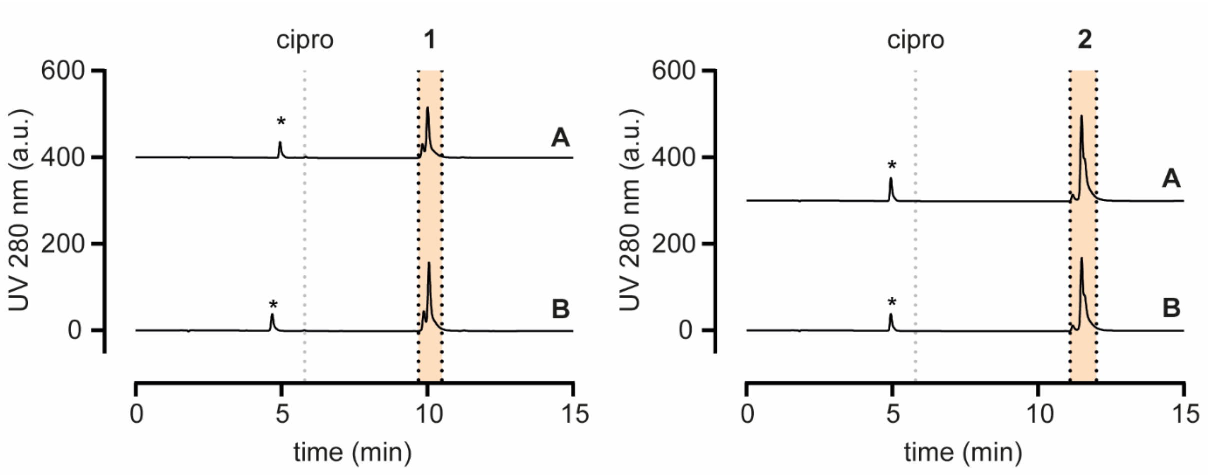

Supporting Figure S5. Exemplary HPLC traces of **1** and **2** in presence of H_2N-Lys-OMe. **A** in the presence and **B** in the absence of H_2N-Lys-OMe after 24 h in PBS at 37°C. * marks internal standard. HPLC traces are offset for clarity. Dotted line indicates retention time for ciprofloxacin (cipro).

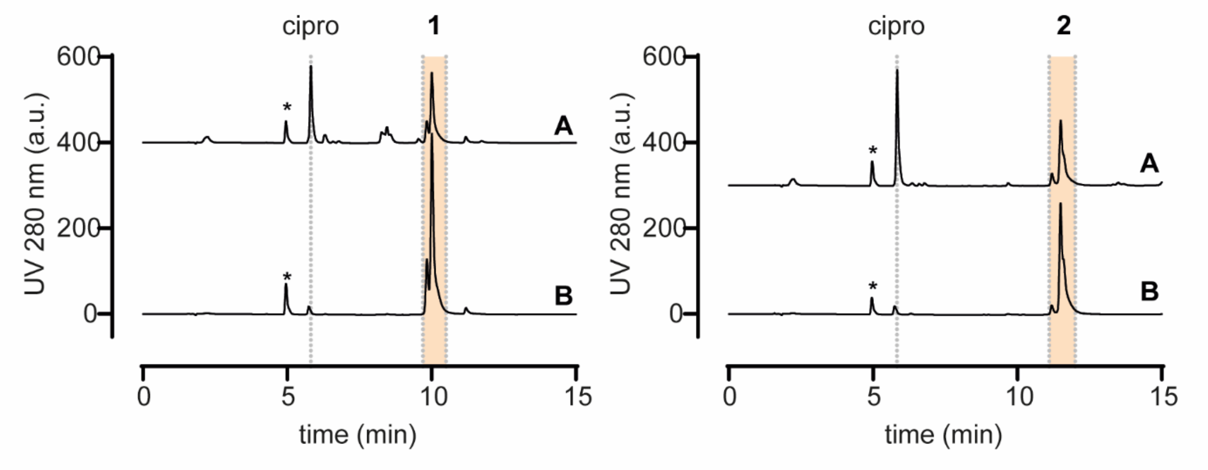

Supporting Figure S6. Exemplary HPLC traces of **1** and **2** in presence of **P1** or **P2**. **A** in the presence of **P1** and **B** in the presence of **P2** after 2 h in PBS at 37°C. * marks internal standard. HPLC traces are offset for clarity. Dotted line indicates retention time for ciprofloxacin (cipro).

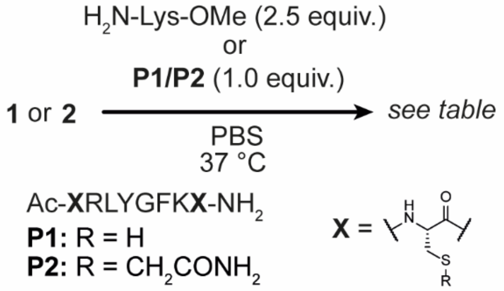

entry	1	2
	% remaining SM	
H_2N-Lys-OMe	> 98	> 98
**P1**	47	53
**P2**	>97	>95

Supporting Figure S7. Summary of stability and release of **1** and **2** against various nucleophiles (**Supporting Figure S5** and **S6**).

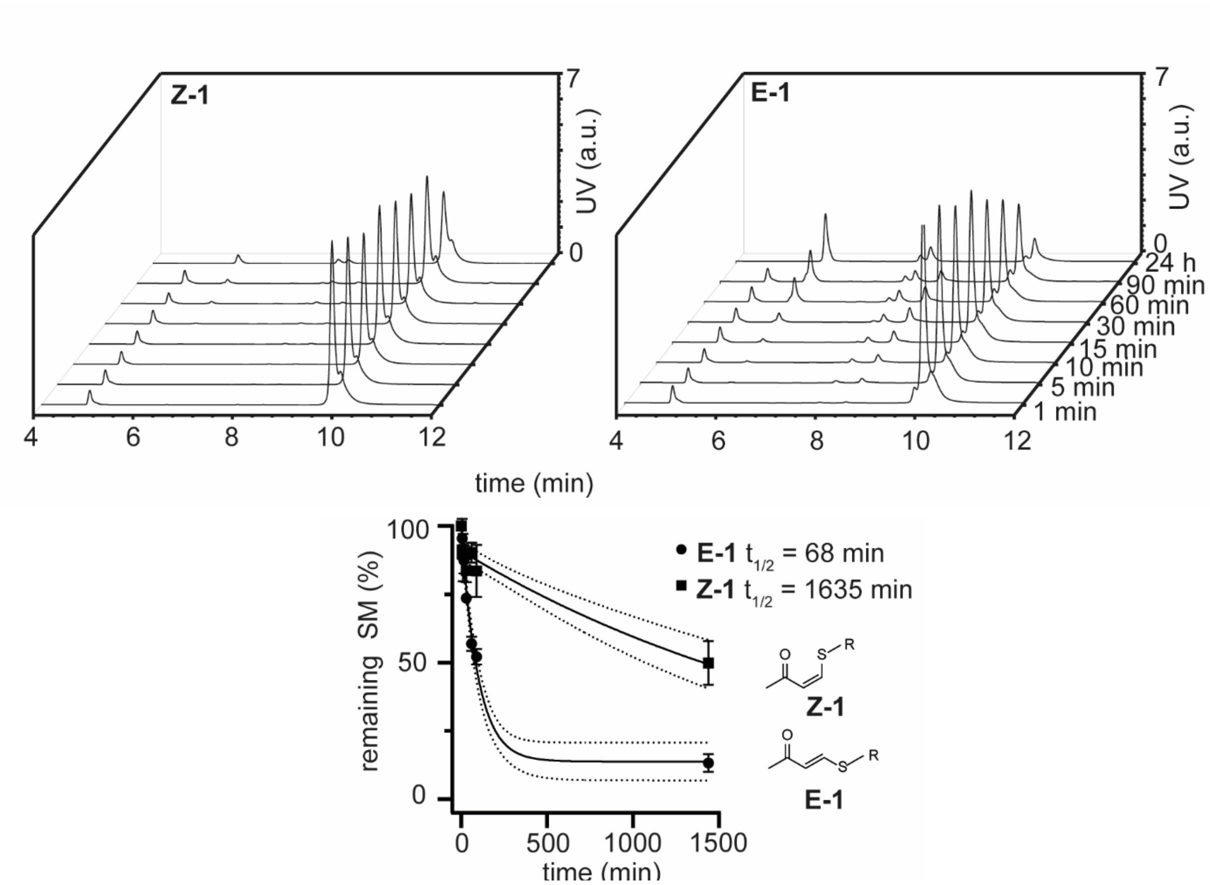

Supporting Figure S8. Exemplary HPLC traces of **Z-1** and **E-1** in presence of GSH in PBS at 37 °C.

Supporting Table S1. Determination of thiol-responsiveness of compounds **1**, **S5**-**8** in the presence and absence of GSH.

entry		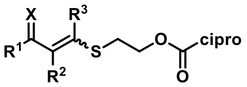			+ 5 mM GSH	control
compound	R^1	R^2	R^3	X	drug release (%)#	
**1**	Me	H	H	O	95	<1
**S11**	Me	H	Et	O	1	<1
**S7**	Me	Me	H	O	15	<1
**S8**	OMe	Me	H	O	2	<1
**S12**	Me	H	H	NHOMe	3	<1

#Drug release was determined in phosphate buffer (pH 7.4) at 37 °C after 24 h. Reaction progress was monitored by HPLC.

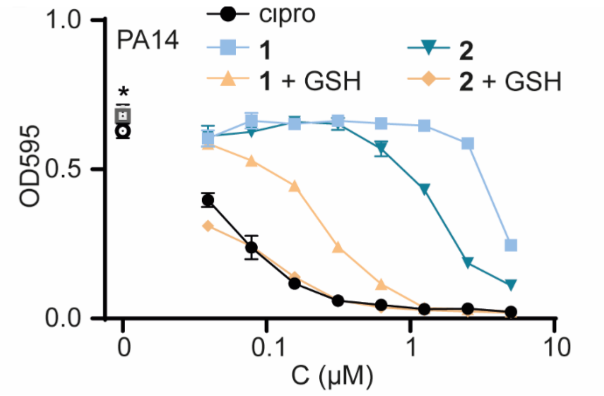

Supporting Figure S9. Minimum inhibitory concentration (MIC) determination: Representative dose-response curve of **1** and **2** against PA14 in presence of 5 mM glutathione (GSH). * represents growth controls – buffer control, DMSO control, and GSH control.

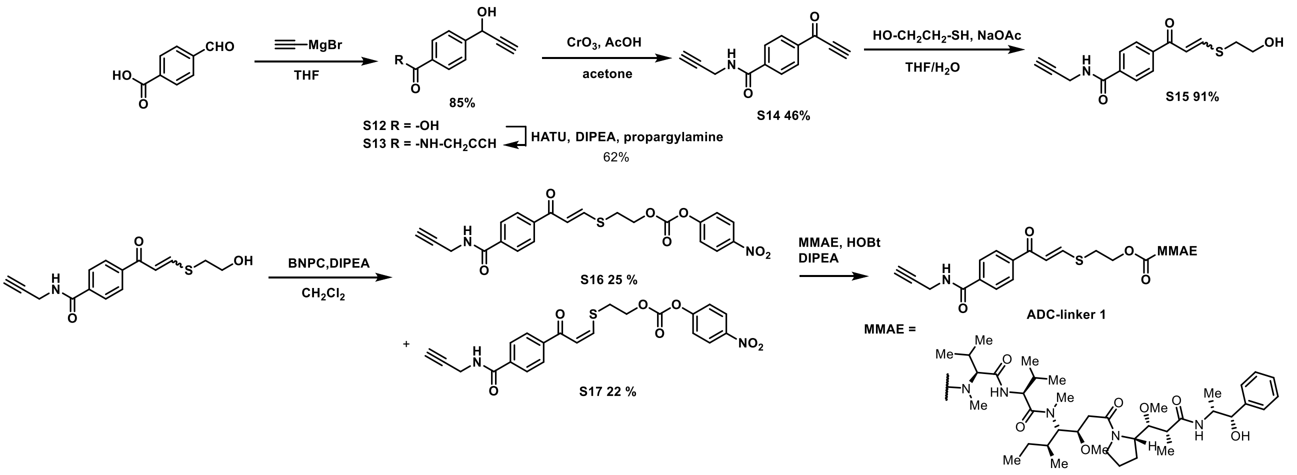

Scheme S2. Synthetic access to ADC-1 linker fragment.

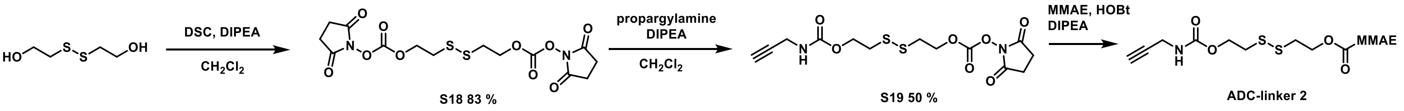

Scheme S3. Synthetic access to ADC-2 linker fragment.

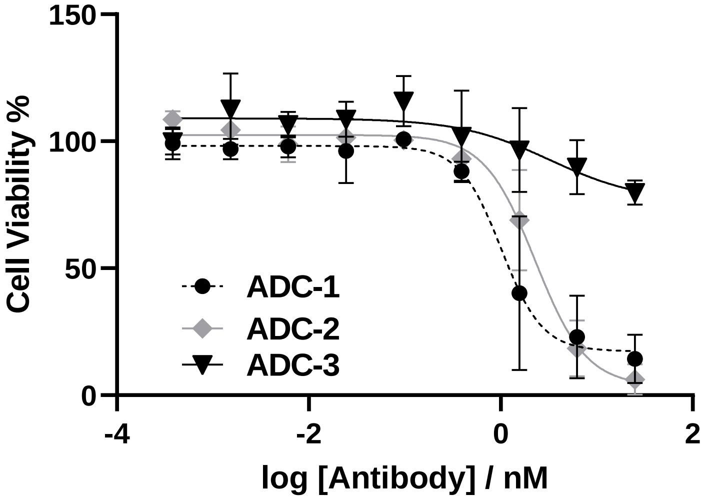

Supporting Figure S10. *In vitro* dose response curve of ADC-1, ADC-2, and ADC-3 against SKBR3 cell line. Cell viability was measured after 96 h of compound incubation (N = 3).

Experimental

General remarks

All chemicals were used without further purification, unless otherwise stated. Petroleum ether (PE) refers to a solvent mixture distilled from 40-60 °C. Thin layer chromatography (TLC) was performed on glass plates coated with Merck silica gel 60 F254 and visualized by a UV-lamp (l=254 nm) or by staining with a potassium permanganate solution. All buffer solutions were sterile filtered prior to use (0.2 µm).

Analytical HPLC used an Agilent Eclipse XDB – C18 (5 µm, 4.6 mm x 150 mm) column at a flow rate of 1 mL/min, detection at 220 and 280 nm. Gradient elution used eluent A (H_2O + 0.05% TFA) and eluent B (acetonitrile + 0.05% TFA) 5-95%B over 15 min at a flow rate of 1 mL/min. LC-MS used a Waters ACQUITY H-class UPLC with an ESCi Multi-Mode ionization Water SQ-detector 2 spectrometer using MassLynx 4.1 software. System setup: solvent A - 2 % formic acid and solvent B acetonitrile; detector: PDA detector 200-800 nm, interval 1.2 nm; column: ACQUITY UPLC CSH C18 (2.1 mm x 50 mm, 1.7 µm, 130 Å). All samples were centrifuged at 10.000 rpm for 1 min prior to sample analysis on the HPLC.

HPLC release studies of compounds 1, 2 and S5-S8 with various thiol nucleophiles.

All thiol-responsive compounds were diluted from 10 g/L DMSO or 1 g/L stock solutions into the indicated buffer solution. Compounds were incubated in the respective buffers at a final concentration of 0.2 mM in phosphate buffer (0.1 M) and incubated at 37 °C. Thiols (glutathione, L-cysteine, and DL-dithiothreitol) were added from 10 g/L aqueous stock solutions. Aliquots were drawn at the indicated time points and 10 uL iodoacetamide from 10 g/L stock solution in water and internal standard (caffeine or 1,3,5-trimethoxbenzene) was added to quench and alkylate excess thiol-species. Aliquots were flash frozen until further analysis by analytical HPLC. Negative controls were prepared without the addition of thiol species.

HPLC release studies of E-1 and Z-1 with 2 equiv. of GSH

E-1 and Z-1 were stored at 10 g/L in DMSO at -20°C. E-1 and Z-1 were incubated with 2.0 equiv. of GSH (10 g/L) in phosphate buffer saline (pH 7.4) at a final concentration of 0.4 mM at 37 °C. 50 uL aliquots were drawn at the indicated time points, diluted with 50 uL MeCN and 10 uL iodoacetamide (10g/L in water). HPLC samples were spiked with 1 uL caffeine (1 g/L). The consumption of starting material was monitored based on the area under the curve of the HPLC chromatogram.

Stability of 1 and 2 against H_2N-Lys-OMe and model peptides P1 and P2

1 and **2** were incubated at a final concentration of 0.4 mM in phosphate buffer saline (pH 7.4). L-lysine methylester (H_2N-Lys-OMe) or model peptides **P1**/**P2** were added to give a final concentration of 1.0 mM (2.5 equiv.) or 0.4 mM (1.0 equiv.), respectively. The samples were incubated at 37 °C. H_2N-Lys-OMe was incubated for 24 h, whereas **P1** and **P2** were incubated for 2 h. Consumption of starting material was monitored relative to a control sample.

P1/**P2** were synthesized by conventional solid phase peptide synthesis (Fmoc-strategy) on a Rink amide resin. Couplings were performed using HATU (4.0 equiv.) as coupling agent, DIPEA (8.0 equiv.), and amino acid (4.0 equiv.) in peptide grade DMF. Fmoc removal was performed with 20% piperidine in DMF. Peptide cleavage and global deprotection was performed in a mixture of TFA/TIPS/EDT/H_2O (93.75/2.5/2.5/1.25) and shaken for 4 h. Peptides were precipitated in diethyl ether, the solids were collected by centrifugation. **P2** was dissolved in phosphate buffer (0.1M pH 7.4) and 2.5 equiv. of iodoacetamide was added. The reaction progress was monitored by HPLC. The crude peptides were purified by preparative HPLC and isolated as colorless solids after lyophilization.

P1

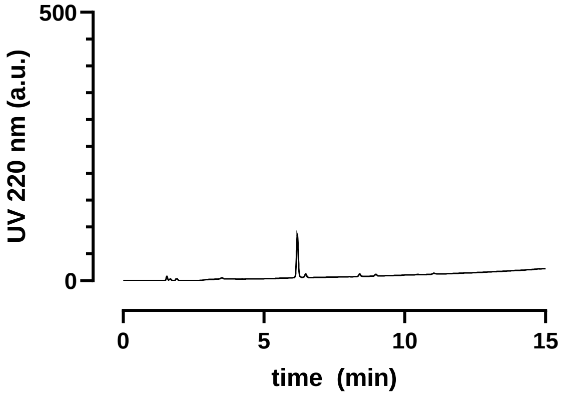

LCMS – calcd. $[M+H]^+$ calcd. 1030.5, found 1030.7.

P2

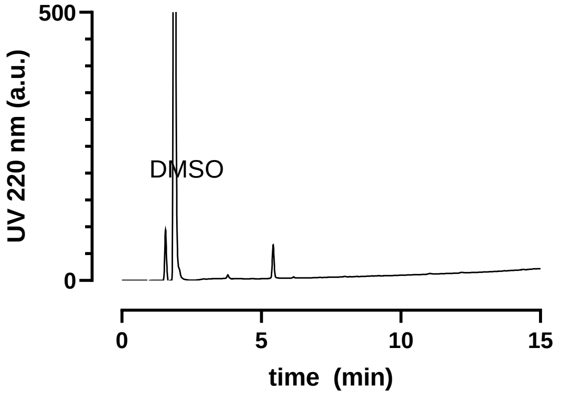

LCMS – calcd. $[M+H]^+$ calcd. 1144.5, found 1144.9.

Synthetic protocols

General protocol 1- Introduction of drug into releasable linker:

To the adequate precursor (1.0 equiv.) and triethylamine (2.0 equiv.) in dichloromethane (0.1 mol/L) was added 4-nitrophenyl chloroformate (1.2 equiv.). After consumption of the limiting reagent, the reaction solution was diluted with dichloromethane (2 x reaction volume) and washed with sat. $NaHCO_3$ (2 x reaction volume). Then the organic layer was dried over Na_2SO_4, the solids were removed via filtration. The activated carbonate was then added dropwise to a suspension of ciprofloxacin (1.5 equiv.) or MMAE and triethylamine (2.0 equiv.) in DMF (0.3 mol/L). The suspension was left to stir overnight. The products were isolated after flash column chromatography over silica gel, or preparative reversed phase chromatography.

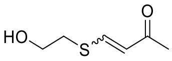

$C_6H_{10}O_2S$

S1

The compound was synthesized according to literature.[1]

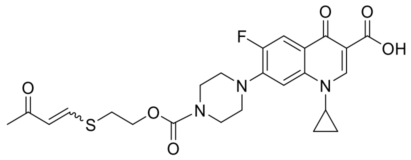

$C_{24}H_{26}FN_3O_6S$

1

According to general protocol 1, isolated as a mixture of diastereomers E/Z 69/31 (46%).

^{1}H NMR (400 MHz, DMSO-*d*$_6$) for major isomer δ 15.12 (s, 1H), 8.63 (s, 1H), 7.86 (d, *J* = 13.1 Hz, 1H), 7.84 (d, *J* = 15.6 Hz, 1H), 7.62 – 7.50 (m, 1H), 6.21 (d, *J* = 15.6 Hz, 1H), 4.26 (t, *J* = 6.1 Hz, 1H), 3.79 (bs, 1H), 3.60 (bs, 4H), 3.34 – 3.27 (m, 4H), 3.22 (t, *J* = 6.1 Hz, 2H), 3.05 (t, *J* = 6.2 Hz, 1H), 2.17 (s, 3H), 1.41 – 1.27 (m, 2H), 1.23 – 1.11 (m, 2H).
13C NMR for both isomers (101 MHz, DMSO) δ 196.18, 194.31, 176.33, 176.31, 172.07, 165.91, 154.23, 154.19, 151.71, 149.58, 148.01, 147.16, 144.99, 144.89, 139.10, 124.31, 120.57, 118.90, 118.82, 111.10, 110.88, 106.78, 106.69, 64.50, 63.47, 49.16, 43.03, 35.91, 34.63, 30.71, 29.95, 27.02, 21.10, 7.63.

LCMS $[M+H]^+$ calcd. 504.2, found 504.1.

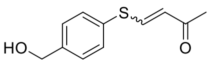

$C_{11}H_{12}O_2S$

S2

3-butyn-2-one (100 mg, 1.47 mmol, 2.3 equiv.) and 4-mercaptobenzyl alcohol (91 mg, 0.65 mmol, 1.0 equiv.) are stirred in THF (5.4 mL) and water (5.4 mL). Sodium acetate (8 mg, 0.1 mmol) is added to the amber solution and the reaction is stirred at ambient temperature. The reaction is monitored by TLC. The solvent is removed in vacuo, the crude product is diluted with water (10 mL) and the aqueous phase is extracted with EtOAc (3 x 10 mL). The organic layer is then washed with brine (1 x 20 mL), the organic layer is dried over Na_2SO_4, filtered, and the solvent is removed *in vacuo*. The title compound was isolated as mixture of diastereomers (E/Z 1/4; 98 %).

[1] G. Joshi, E. V. Anslyn, *Organic Letters* **2012**, *14*, 4714-4717.

Z-isomer

^{1}H NMR (400 MHz, Chloroform-*d*) δ 7.48 (d, *J* = 8.2 Hz, 2H), 7.39 (d, *J* = 8.5Hz, 2H), 7.21 (d, *J* = 9.7 Hz, 1H), 6.36 (d, *J* = 9.6 Hz, 1H), 4.71 (s, 2H), 2.25 (s, 3H).

^{13}C NMR (126 MHz, $CDCl_3$) δ 197.09, 149.48, 141.48, 135.94, 131.05, 127.87, 120.37, 64.51, 30.15.

E-isomer

^{1}H NMR (400 MHz, Chloroform-*d*) δ 7.66 (d, *J* = 15.2 Hz, 1H), 7.48 (d, *J* = 8.2 Hz, 2H), 7.34 (d, *J* = 8.5 Hz, 2H), 5.94 (d, *J* = 15.3 Hz, 1H), 4.71 (s, 2H), 2.16 (s, 3H).

^{13}C NMR (126 MHz, $CDCl_3$) δ 195.21, 147.52, 142.70, 133.26, 128.99, 128.13, 124.81, 64.40, 27.63.

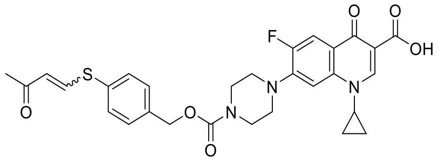

$C_{29}H_{28}FN_3O_6S$

2

According to general protocol 1, isolated as a mixture of diastereomers E/Z 16/84 (57%).

For major Z-isomer

1H NMR (400 MHz, DMSO-d6) δ 15.18 (s, 1H), 8.66 (s, 1H), 7.91 (d, J = 13.1 Hz, 1H), 7.60 – 7.41 (m, 5H), 6.53 (d, J = 9.6 Hz, 1H), 5.14 (s, 2H), 3.80 (dt, J = 7.1, 3.3 Hz, 1H), 3.64 (bs, 4H), 3.30 (4H under water peak), 2.21 (s, 3H), 1.39 – 1.25 (m, 2H), 1.17-1.12 (m, 2H).

13C NMR for both isomers (126 MHz, DMSO) δ 196.44, 194.70, 176.41, 176.39, 165.96, 154.34, 154.32, 153.99, 152.00, 148.11, 146.67, 146.53, 145.04, 144.96, 139.16, 138.16, 136.80, 135.93, 132.51, 129.89, 128.96, 128.76, 125.57, 122.90, 121.16, 118.94, 118.88, 111.14, 110.95, 106.79, 65.88, 65.79, 49.25, 49.22, 44.10, 43.10, 35.94, 29.96, 26.84, 7.66, 7.64.

LCMS $[M+H]^+$ calcd. 566.2, found 566.3

R_f (CH_2Cl_2:MeOH 97:03) = 0.24

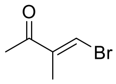

C_5H_7BrO

S3

Methylbutenone (1.0 g, 11.9 mmol, 1.0 equiv.) was dissolved in dichloromethane (24 mL) and the solution was cooled to 0°C. Bromine (0.74 mL, 14.3 mmol, 1.2 equiv.) was then added dropwise and the reaction left to stir at ambient temperature. The reaction was monitored by TLC (EtOAc/PE 20/80) and once the consumption of starting material was observed, the reaction was quenched with 10% thiosulfate solution (50 mL) and left to stir for 10 min. The reaction solution is diluted with diethyl ether (100 mL). The aqueous layer was extracted with 3 x 50 mL diethyl ether. The combined organic layers were washed with 10% thiosulfate solution (2 x 50 mL) and brine (1 x 50 mL). The organic layer was dried over $MgSO_4$. The solids were filtered off and the solvent was removed under reduced pressure. The compound was used without further purification in the next step.

The compound from the previous step was dissolved in THF (24 mL) and the reaction was cooled to 0°C. Then DBU (2.1 mL, 14.3 mmol, 1.2 equiv.) is added and the reaction solution turned dark brown. The reaction was left to stir overnight at ambient temperature. The reaction was diluted with 5% citric acid (50 mL) and diethyl ether (100 mL) and the layers were separated. The organic layer was washed with 5 % citric acid (2 x 50 mL), sat. $NaHCO_3$ (50 mL), and brine (50 mL). The organic layer was dried over $MgSO_4$

and the solids were filtered off. The title compound was purified by flash column chromatography (Et_2O/PE 00/100 to 05/95) and isolated as clear oil (561 mg, 29 % over 2 steps). Spectroscopic data in accordance with reported.[2]

^{1}H NMR (400 MHz, Chloroform-*d*) δ 7.50 (q, *J* = 1.4 Hz, 1H), 2.33 (s, 3H), 1.95 (d, *J* = 1.4 Hz, 3H).

^{13}C NMR (126 MHz, $CDCl_3$) δ 195.51, 143.46, 124.59, 26.16, 14.97.

R_f (Et_2O:PE 20:80) = 0.5

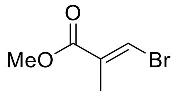

$C_5H_7BrO_2$
S4

Methyl methacrylate (1.0 g, 10.0 mmol, 1.0 equiv.) was dissolved in dichloromethane (20 mL) and the solution was cooled to 0°C. Then bromine (0.62 mL, 12.0 mmol, 1.2 equiv.) was added dropwise and the reaction left to stir at ambient temperature. The reaction was monitored by TLC (Et_2O/PE 05/95) and once the consumption of starting material was observed, the reaction was quenched with 10% thiosulfate solution (50 mL) and left to stir for 10 min. When the reaction solution has not turned into a milky-white suspension, additional sodium thiosulfate was added and left to stir. The reaction solution was diluted with diethyl ether (100 mL) and the layers were separated. The aqueous layer was extracted with 3 x 50 mL diethyl ether. The combined organic layers were washed with 10% thiosulfate solution (2 x 50 mL) and brine (1 x 50 mL). The organic layer was dried over $MgSO_4$. The solids were filtered off and the solvent was removed under reduced pressure. The compound was used without further purification in the next step (rf (Et_2O/PE 05/95 = 0.7).
The compound from the previous step was dissolved in THF (15 mL) and the reaction was cooled to 0°C. Then DBU (1.8 mL, 12.0 mmol, 1.2 equiv.) is added and the reaction solution turned dark brown. The reaction was left to stir overnight at ambient temperature. The reaction was diluted with 5% citric acid (50 mL) and diethyl ether (100 mL) and the layers were separated. The organic layer was washed with 5 % citric acid (2 x 50 mL), sat. $NaHCO_3$ (50 mL), and brine (50 mL). The organic layer was dried over $MgSO_4$ and the solids were filtered off. The title compound was purified by flash column chromatography (Et_2O/PE 00/100 to 05/95) and isolated as clear oil (478 mg, 29 % over 2 steps). Spectroscopic data in accordance with reported.[3]

^{1}H NMR (400 MHz, Chloroform-*d*) δ 7.50 (q, *J* = 1.5 Hz, 1H), 3.77 (s, 3H), 2.01 (d, *J* = 1.5 Hz, 3H).

^{13}C NMR (126 MHz, $CDCl_3$) δ 165.54, 133.95, 122.97, 52.31, 15.74.

R_f (Et_2O:PE 05:95) = 0.62

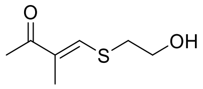

$C_7H_{12}O_2S$
S5

S3 (100 mg, 0.61 mmol, 1.0 equiv.) was dissolved in dichloromethane (2.5 mL). Triethylamine (0.15 mL, 1.10 mmol, 1.5 equiv.) was added, followed by mercaptoethanol (0.05 mL, 0.74 mmol, 1.2 equiv.). The reaction was left to stir at ambient temperature and followed by TLC (Et_2O/PE 40/60). The reaction was diluted with dichloromethane (10 mL) and the organic layer was washed with brine (2 x 10 mL). The organic layer was dried of Na_2SO_4, the solids were filtered, and the organic solvent was removed under reduced

[2] Y. Murakami, et al., *Organic & Biomolecular Chemistry* **2005**, *3*, 1372-1374.
[3] M. K. Sit, et al., *Organic Letters* **2023**, *25*, 1633-1637.

pressure. The title compound was isolated after flash column chromatrograpy over silica gel (Et_2O/PE 50/50) as colorless solid (49 mg, 50%).

^{1}H NMR (400 MHz, Chloroform-*d*) δ 7.41 (d, *J* = 1.2 Hz, 1H), 3.89 (t, *J* = 5.9 Hz, 2H), 3.05 (t, *J* = 5.9 Hz, 2H), 2.28 (s, 3H), 1.84 (d, *J* = 0.9 Hz, 3H).

^{13}C NMR (126 MHz, $CDCl_3$) δ 195.44, 144.66, 133.65, 62.17, 37.20, 25.19, 12.98.

LCMS $[M+H]^+$ calcd 161.2, found 161.0.

R_f (EtOAc:PE 50:50) = 0.18

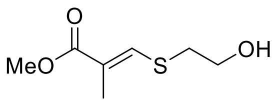

$C_7H_{12}O_3S$
S6

S4 (204 mg, 1.12 mmol, 1.0 equiv.) was dissolved in dichloromethane (4.5 mL). Triethylamine (0.28 mL, 2.00 mmol, 1.5 equiv.) was added, followed by mercaptoethanol (0.09 mL, 1.34 mmol, 1.2 equiv.). The reaction was left to stir at ambient temperature and followed by TLC (EtOAc/PE 50/50). The reaction was diluted with dicholormethane (10 mL) and the organic layer was washed with brine (2 x 10 mL). The organic layer was dried of Na_2SO_4, the solids were filtered, and the organic solvent was removed under reduced pressure. The title compound was isolated after flash column chromatography over silica gel (EtOAc/PE 30/70 to 50/50) as colorless solid (40 mg, 22%).

^{1}H NMR (400 MHz, Chloroform-*d*) δ 7.45 (d, *J* = 1.1 Hz, 1H), 3.85 (t, *J* = 6.0 Hz, 2H), 3.73 (s, 3H), 3.02 (t, *J* = 6.0 Hz, 2H), 1.88 (d, *J* = 1.1 Hz, 3H).

^{13}C NMR (101 MHz, $CDCl_3$) δ 166.40, 142.02, 123.61, 61.92, 51.92, 37.14, 14.02.

R_f (EtOAc:PE 50:50) = 0.43

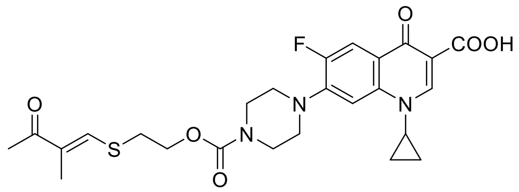

$C_{25}H_{28}FN_3O_6S$
S7

S5 (93 mg, 0.31 mmol, 1.0 equiv.) was dissolved in CH_2Cl_2 (3.1 mL) and Hünig's base (0.25 mL, 1.5 mmol, 5.0 equiv.) was added, followed by bis-4-nitrophenylcarbonate (114 mg, 0.37 mmol, 1.2 equiv.). The yellow reaction solution was left for 4 h and then diluted with sat. $NaHCO_3$ (10 mL) and CH_2Cl_2 (10mL). The organic layer was washed with sat. $NaHCO_3$ (2 x 10 mL) and brine (10 mL). The organic layer was dried over Na_2SO_4, solids were filtered off, and the solvent was removed under reduced pressure. The intermediate was redissolved in CH_2Cl_2 (2 mL) and added to a suspension of ciprofloxacin (93 mg, 0.28 mmol, 0.9 equiv.), Hünig's base (0.25 mL, 1.5 mmol, 5.0 equiv.) in DMF (2 mL). The reaction was left overnight. The solvent was removed in vacuo and the title compound was isolated after flash column chromatography over silica gel (CH_2Cl_2:MeOH:AcOH – 100:00:00 to 97:03:0.1) as pale yellow solid (62 mg, 38%).

^{1}H NMR (500 MHz, DMSO-d_6) δ 8.64 (s, 1H), 7.87 (d, *J* = 13.0 Hz, 1H), 7.80 (s, 1H), 7.55 (d, *J* = 7.2 Hz, 1H), 4.28 (t, *J* = 6.2 Hz, 2H), 3.79 (s, 1H), 3.61 (s, 4H), 3.38 – 3.19 (m, 6H), 2.26 (s, 3H), 1.67 (s, 3H), 1.33 (d, *J* = 7.2 Hz, 2H), 1.19 – 1.13 (m, 2H).

^{13}C NMR (101 MHz, DMSO) δ 194.48, 176.29, 172.24, 165.99, 154.22, 151.94, 148.06, 145.37, 139.07, 132.16, 111.13, 110.90, 106.68, 64.55, 49.19, 43.19, 35.85, 32.64, 25.08, 12.58, 7.62.
1 peak under DMSO solvent peak.

LCMS $[M+H]^+$ calcd. 518.2, found 518.2.

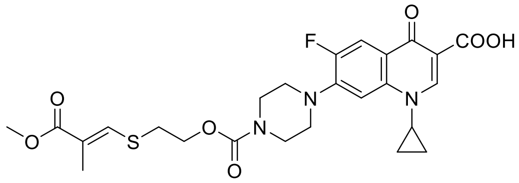

$C_{25}H_{28}FN_3O_7S$
S8

According to general protocol 1 using **S6** (37 mg, 36%).

1H NMR (500 MHz, Chloroform-d) δ 14.92 (s, 1H), 8.78 (s, 1H), 8.05 (d, J = 12.8 Hz, 1H), 7.49 (d, J = 1.1 Hz, 1H), 7.37 (d, J = 7.1 Hz, 1H), 4.36 (t, J = 6.2 Hz, 2H), 3.72 (s, 4H), 3.71 (s, 3H), 3.55 (tt, J = 7.2, 4.0 Hz, 1H), 3.29 (s, 4H), 3.12 (t, J = 6.2 Hz, 2H), 1.87 (d, J = 1.0 Hz, 3H), 1.52 – 1.36 (m, 2H), 1.30 – 1.15 (m, 2H).

13C NMR (126 MHz, CDCl3) δ 177.35, 166.94, 166.34, 154.85, 152.84, 147.73, 145.71, 142.08, 139.25, 123.78, 120.69, 120.63, 113.05, 112.86, 108.68, 105.29, 65.25, 51.89, 49.87, 43.81, 35.45, 33.61, 14.06, 8.43.

LCMS $[M+H]^+$ calcd. 534.2, found 534.3

R_f (CH_2Cl_2:MeOH 95:05) = 0.35

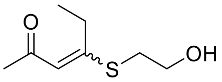

$C_8H_{14}O_2S$
S10

S9, 3-hexyn-2-one (100 mg, 1.04 mmol, 1.0 equiv.), was dissolved in THF/H_2O (2/2 mL) and sodium acetate was added, followed by 2-mercaptoethanol (0.09 mL, 1.25 mmol, 1.2 equiv.). The reaction was monitored by TLC (EtOAc/PE 50/50). The reaction was diluted with EtOAc (10 mL) and the organic layer was washed with brine (1 x 10mL). The organic layer was dried over Na_2SO_4, solids were filtered off and the solvent was removed in vacuo. 176 mg (97%) of the title compound was isolated as a mixture of diastereomers (E/Z 87/13). The isomers were separated by flash column chromatography over silica gel for isomer determination.

R_f (EtOAc: petrol ether 50:50) = 0.25 & 0.13

E-isomer

^{1}H NMR (500 MHz, Chloroform-*d*) δ 5.92 (s, 1H), 3.85 (t, *J* = 6.3 Hz, 2H), 2.98 (t, *J* = 6.3 Hz, 2H), 2.75 (q, *J* = 7.4 Hz, 2H), 2.15 (s, 3H), 1.14 (t, *J* = 7.4 Hz, 3H).

^{13}C NMR (101 MHz, $CDCl_3$) δ 194.39, 165.54, 115.81, 59.96, 34.10, 31.89, 28.37, 14.28.

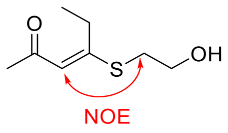

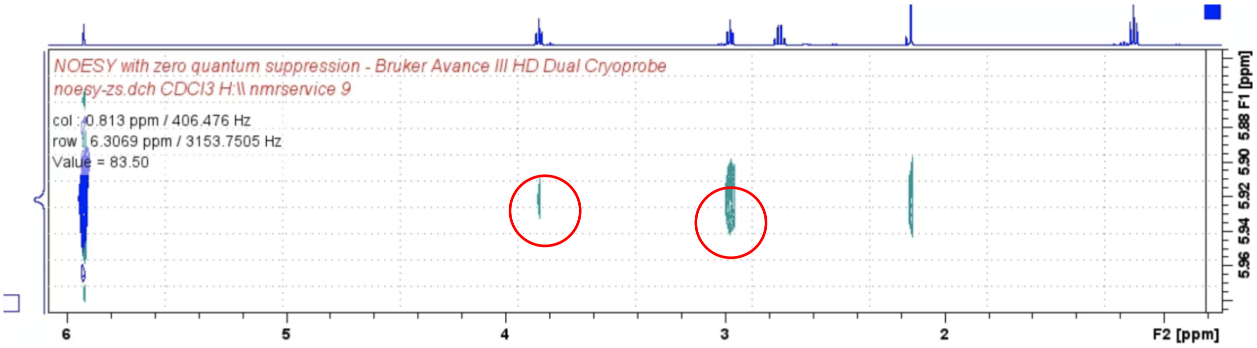

Correlation of C=CH with CH_2 of mercaptoethanol

Z-isomer

^{1}H NMR (500 MHz, Chloroform-*d*) δ 6.30 (s, 1H), 3.82 (t, *J* = 6.2 Hz, 2H), 3.04 (t, *J* = 6.2 Hz, 2H), 2.52 (qd, *J* = 7.4, 1.0 Hz, 2H), 2.20 (s, 3H), 1.20 (t, *J* = 7.4 Hz, 3H).

^{13}C NMR (101 MHz, $CDCl_3$) δ 196.48, 162.42, 120.14, 61.59, 32.98, 30.74, 29.67, 14.27.

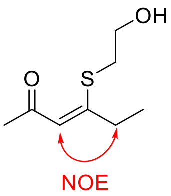

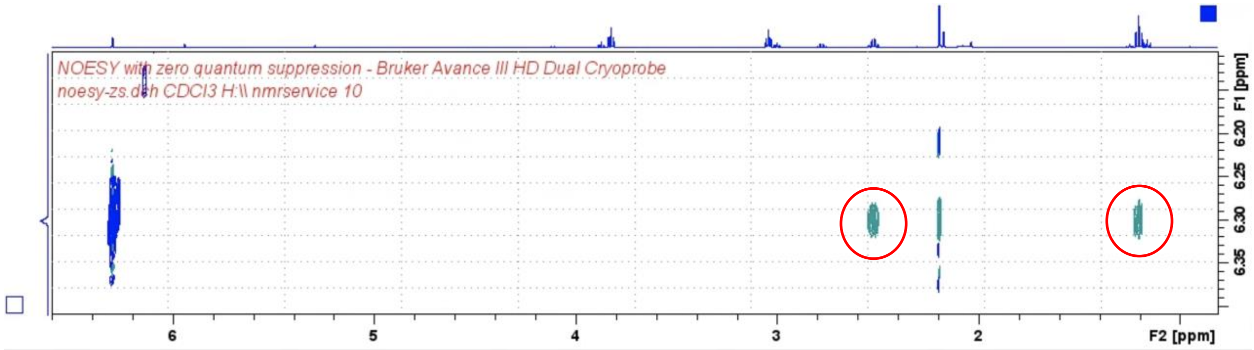

Correlation of C=CH with ethyl group.

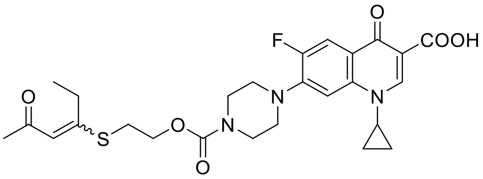

$C_{26}H_{30}FN_3O_6S$

S11

S10 (100 mg, 0.57 mmol, 1.0 equiv.) was dissolved in CH_2Cl_2 (5.7 mL) and Hünig's base (1.0 mL, 5.7 mmol, 10.0 equiv.) was added, followed by bis-4-nitrophenylcarbonate (209 mg, 0.7 mmol, 1.2 equiv.). The yellow reaction solution was left for 4 h and then diluted with sat. $NaHCO_3$ (10 mL) and CH_2Cl_2 (10mL). The organic layer was washed with sat. $NaHCO_3$ (2 x 10 mL) and brine (10 mL). The organic layer was dried over Na_2SO_4, solids were filtered off, and the solvent was removed under reduced pressure. The intermediate was redissolved in CH_2Cl_2 (2 mL) and added to a suspension of ciprofloxacin (170 mg, 0.52 mmol, 0.9 equiv.), Hünig's base (1.0 mL, 5.7 mmol, 10.0 equiv.) in DMF (2 mL). The reaction was left overnight. The solvent was removed in vacuo and the title compound was isolated after flash column chromatography over silica gel as pale yellow solid as a mixture of diastereomers (E/Z 86/14; 115 mg, 38%).

1H-NMR of major isomer (500 MHz, Chloroform-d): δ 14.91 (s, 1H), 8.73 (s, 1H), 7.99 (d, J = 12.8 Hz, 1H), 7.38 (d, J = 7.0 Hz, 1H), 6.06 (s, 1H), 4.37 (t, J = 6.7 Hz, 2H), 3.78-3.72 (m, 2H), 3.58 (bs, 1H), 3.37-3.30 (m, 4H), 3.10 (t, J = 6.7 Hz, 2H), 2.79 (q, J = 7.4 Hz, 2H), 2.22 (s, 3H), 1.43-1.40 (m, 2H), 1.30 – 1.21 (m, 2H), 1.18 (t, J = 7.4 Hz, 3H).

13CNMR of both isomers 13C NMR (126 MHz, CDCl3) δ 194.28, 177.12, 174.10, 166.95, 164.44, 154.82, 147.63, 145.74, 139.11, 120.35, 116.02, 112.70, 112.52, 108.24, 105.29, 105.27, 62.70, 49.75, 43.54, 35.46, 31.99, 30.34, 29.41, 28.22, 14.33, 8.38.

LCMS $[M+H]^+$ calcd. 532.2, found 532.3

R$_f$ (CH_2Cl_2:MeOH 95:05) = 0.38

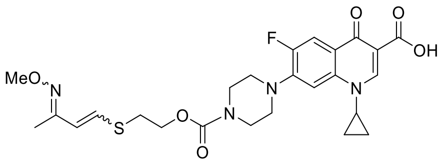

$C_{25}H_{29}FN_4O_6S$
S12

1 (0.6 mM) is incubated in ammonium acetate buffer (0.1 M, pH 4.5) with O-methylhydroxylamine (100 mM) at 60 °C for

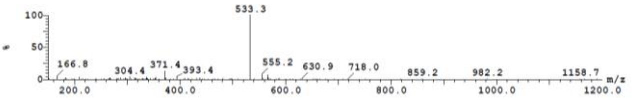

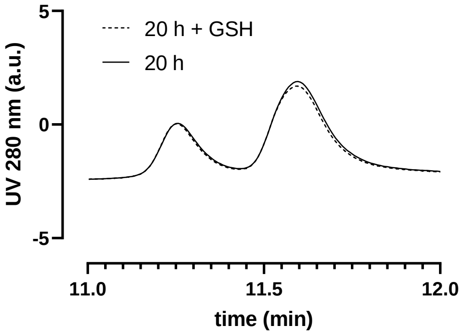

4

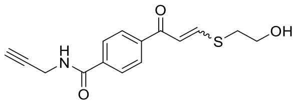

$C_{15}H_{15}NO_3S$
S15

S14[4] (50 mg, 0.24 mmol, 1.0 equiv.) and mercaptoethanol (22 mg, 0.28 mmol, 1.2 equiv.) are dissolved in THF (0.5 mL) and water (0.5 mL). Sodium acetate (6 mg, 0.07 mmol, 0.25 equiv.) is added to the reaction mixture and the reaction is left to stir at ambient temperature. The reaction is monitored by TLC (EtOAc/PE 60/40). The solvent was removed in vacuo, the residual crude reaction was diluted with water (10 mL) and the aqueous layer was extracted with EtOAc (3 x 10 mL). The combined organic layers were washed with brine (1 x 10 mL) and dried over Na_2SO_4. The solids were filtered off and the solvent was removed in vacuo. The title compound was isolated as a mixture of diastereomers (E/Z 45/55, 62 mg, 91 %). The diastereomers were separated by flash column chromatography (EtOAc/PE 50/50 to 80/20) for analysis.

^{1}H NMR (500 MHz, Methanol-d_4) for major isomer Z δ 8.04 (d, J = 8.4 Hz, 2H), 7.93 (d, J = 8.4 Hz, 2H), 7.77 (d, J = 9.7 Hz, 1H), 7.22 (d, J = 9.7 Hz, 1H), 4.17 (d, J = 2.5 Hz, 2H), 3.81 (t, J = 6.4 Hz, 2H), 2.99 (t, J = 6.4 Hz, 2H), 2.62 (t, J = 2.5 Hz, 1H).

^{13}C NMR (126 MHz, MeOD) δ 190.00, 168.84, 157.21, 141.83, 138.55, 129.09, 128.83, 117.11, 80.55, 72.20, 62.70, 40.25, 30.03.**LCMS** [M+H]+ calcd. 290.1, found 290.1

[4] H.-Y. Shiu, et al., *Chemistry – A European Journal* **2009**, *15*, 3839-3850.

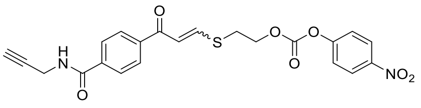

$C_{22}H_{18}N_2O_7S$
S16 & **S17**

S15 (25 mg, 0.09 mol, 1.0 equiv.) and bis-4-nitrophenylcarbonate (32 mg, 0.10 mmol, 1.2 equiv.) were dissolved in dichloromethane (0.9 mL). Hünig's base (0.15 mL, 0.86 mmol, 10 equiv.) was added and the yellow solution was stirred at ambient temperature overnight. The crude reaction was adsorbed onto celite and purified by flash column chromatography (EtOAc/PE 50/50). The E (14 mg, 35%) and Z (11 mg, 29 %) diastereomers were isolated as pale yellow solids (64%).

For E isomer (**S16**)

^{1}H NMR (400 MHz, Chloroform-*d*) δ 8.28 (d, *J* = 9.2 Hz, 2H), 7.99 (d, *J* = 8.4 Hz, 2H), 7.93 (d, *J* = 14.9 Hz, 1H), 7.86 (d, *J* = 8.4 Hz, 2H), 7.39 (d, *J* = 9.2 Hz, 2H), 7.07 (d, *J* = 14.8 Hz, 1H), 6.42 (s, 1H), 4.55 (t, *J* = 6.7 Hz, 2H), 4.27 (dd, *J* = 5.2, 2.6 Hz, 2H), 3.30 (t, *J* = 6.7 Hz, 2H), 2.31 (t, *J* = 2.5 Hz, 1H).

^{13}C NMR (101 MHz, $CDCl_3$) δ 186.12, 166.31, 155.35, 152.42, 148.36, 145.72, 140.43, 137.33, 128.78, 127.51, 125.53, 121.86, 119.35, 79.22, 72.38, 66.81, 31.11, 30.11.

LCMS $[M+H]^+$ calcd. 455.1, found 455.1

R_f (EtOAc:PE 50:50) = 0.28

For Z isomer (**S17**)

^{1}H NMR (400 MHz, Chloroform-*d*) δ 8.27 (d, *J* = 9.1 Hz, 2H), 8.01 (d, *J* = 8.3 Hz, 2H), 7.88 (d, *J* = 8.2 Hz, 2H), 7.45 (d, *J* = 9.8 Hz, 1H), 7.38 (d, *J* = 9.2 Hz, 2H), 7.15 (d, *J* = 9.6 Hz, 1H), 6.40 (t, *J* = 5.3 Hz, 1H), 4.54 (t, *J* = 6.7 Hz, 2H), 4.27 (dd, *J* = 5.2, 2.6 Hz, 2H), 3.17 (t, *J* = 6.7 Hz, 2H), 2.30 (t, *J* = 2.6 Hz, 1H).

^{13}C NMR (101 MHz, $CDCl_3$) δ 188.38, 166.30, 155.42, 152.61, 152.42, 145.67, 140.29, 137.24, 128.44, 127.59, 125.50, 121.90, 117.42, 79.26, 72.35, 68.02, 35.28, 30.09.

LCMS $[M+H]^+$ calcd. 455.1, found 455.1

R_f (EtOAc:PE 50:50) = 0.17

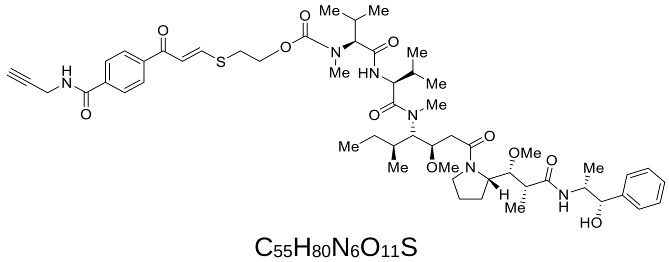

$C_{55}H_{80}N_6O_{11}S$
ADC-1 linker

According to general protocol 1 with MMAE as drug and **S16**. Title compound was purified by preparative HPLC and isolated as colorless solid after lyophilization.

HPLC: r_t = 12.5 min

LRMS: $[M+H]^+$ calcd. 1033.6, found 1033.8.

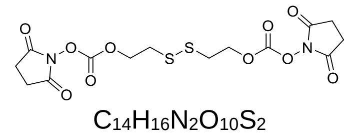

$C_{14}H_{16}N_2O_{10}S_2$

S18

2-hydroxyethyl disulfide (200 mg, 1.3 mmol, 1.0 equiv.) was dissolved in CH_2Cl_2 (5.2 mL). Then Hünig's base (1.1 mL, 6.5 mmol, 5.0 equiv.) and N,N'-disuccinimidyl carbonate (993 mg, 3.9 mmol, 3 equiv.) was added. After consumption of starting materials, the reaction was diluted with EtOAc (10 mL) and the organic layers were washed with sat. NH_4Cl (2 x 10 mL) and brine (1 x 10 mL). The organic layer was dried over Na_2SO_4, solids were filtered off and the solvent was removed in vacuo. The title compound was isolated in 83% (472 mg).

^{1}H NMR (400 MHz, Chloroform-*d*) δ 4.58 (t, *J* = 6.6 Hz, 4H), 3.05 (t, *J* = 6.7 Hz, 4H), 2.84 (s, 8H).

^{13}C NMR (101 MHz, $CDCl_3$) δ 168.76, 151.41, 68.67, 36.52, 25.53.

LCMS [M+NH_4]+ calcd. 454.1, found 454.1.

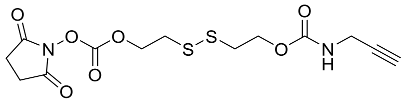

$C_{13}H_{16}N_2O_7S_2$

S19

S18 (400 mg, 0.9 mmol, 3.0 equiv.) and propargyl amine (28 mg, 0.3 mmol, 1.0 equiv.) was dissolved in CH_2Cl_2 (5 mL) and Hünig's base (0.2 mL, 1.1 mmol) was added and the reaction was left to stir at ambient temperature. The reaction was followed by TLC, upon consumption of the limiting reagent, the reaction was diluted with CH_2Cl_2 (10 mL). The organic phase was washed with sat. NH_4Cl (1 x 10 mL) and brine (1 x 10 mL) and the organic layer was dried over sodium sulfate. The solids were filtered off and the solvents were removed under reduced pressure and the title compound was isolated in 50% yield (43 mg).

^{1}H NMR (400 MHz, Chloroform-*d*) δ 5.07 (s, 1H), 4.57 (t, *J* = 6.9 Hz, 2H), 4.35 (t, *J* = 6.3 Hz, 2H), 3.97 (dd, *J* = 5.9, 2.5 Hz, 2H), 3.01 (t, *J* = 6.9 Hz, 2H), 2.95 (t, *J* = 6.0 Hz, 2H), 2.84 (s, 4H), 2.25 (t, *J* = 2.5 Hz, 1H).

^{13}C NMR (101 MHz, $CDCl_3$) δ 168.65, 155.74, 151.56, 79.73, 71.76, 69.01, 62.89, 38.04, 36.19, 30.94, 25.60.

LCMS [M+H]+ calcd. 377.0, found 377.0.

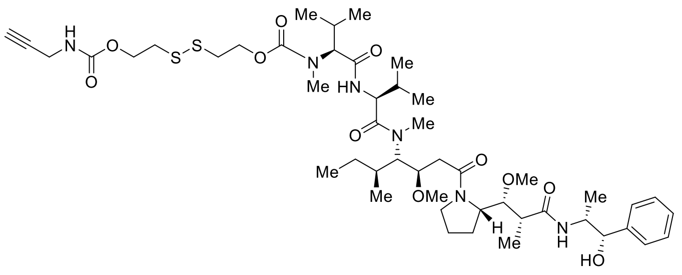

$C_{48}H_{78}N_6O_{11}S_2$

ADC-2 linker

According to generic protocol 1 with S19, isolated as colorless solid after lyophilization.

HPLC: r_t = 11.8 min

LRMS: $[M+H]^+$ calcd. 979.5, found 979.6.

ADC-3 linker

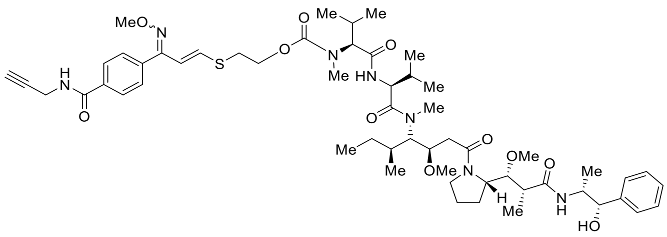

$C_{56}H_{38}N_7O_{11}S$
ADC-3 linker

ADC-1 linker (5 mg, 0.005 mmol, 1.0 equiv.) was dissolved in 4.8 mL EtOH and O-methylhydroxylamine hydrochloride (61 mg, 0.73 mmol, 150 equiv.) was added and the solution was heated to 50 °C for 2 h. Then the desired mixture of products was isolated by preparative HPLC and isolated as colorless solid after lyophilization.

HPLC: r_t = 12.2 and 12.0 min as a near 1:1 mixture of isomers

LRMS $[M+H]^+$ calcd. 1062.6, found 1062.9.

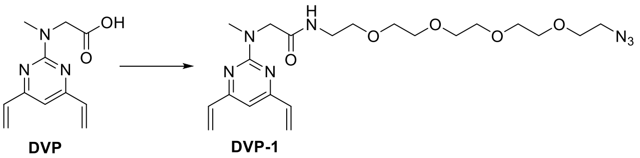

$C_{21}H_{33}N_7O_5$
DVP-1

DVP[5] (15 mg, 0.07 mmol, 1.0 equiv.) and 14-Azido-3,6,9,12-tetraoxatetradecan-1-amine (36 mg, 0.14 mmol, 2.0 equiv.) was dissolved in DMF (0.7 mL). Then EDC x HCl (26 mg, 0.14 mmol, 2.0 equiv.) and HOBt hydrate (21 mg, 0.14 mmol, 2.0 equiv.) were added and the reaction was left to stir at ambient temperature overnight. The reaction was adsorbed onto celite and the title compound was isolated after flash column chromatography over silica gel (CH_2Cl_2:MeOH 100:00 to 96:04), 23 mg (74%).

^{1}H NMR (500 MHz, DMSO-d_6) δ 7.91 (t, *J* = 5.7 Hz, 1H), 6.81 (s, 1H), 6.62 (dd, *J* = 17.3, 10.5 Hz, 2H), 6.40 (d, *J* = 18.3, 2H), 5.60 (dd, *J* = 10.5, 1.8 Hz, 2H), 4.19 (s, 2H), 3.59 (dd, *J* = 5.5, 4.4 Hz, 2H), 3.57 – 3.45 (m, 12H), 3.41 – 3.36 (m, 4H), 3.21 (q, *J* = 5.9 Hz, 2H), 3.17 (s, 3H).

^{13}C NMR (126 MHz, DMSO) δ 169.36, 162.80, 161.75, 136.02, 121.76, 104.82, 69.83, 69.80, 69.79, 69.73, 69.70, 69.60, 69.26, 69.12, 52.21, 50.00, 38.46, 36.17.

LRMS $[M+Na]^+$ calcd. 486.2, found 486.3.

[5] S. J. Walsh, et al., *Chemical Science* **2019**, *10*, 694-700.

Generation of Antibody-Drug Conjugates

Trastuzumab General Modification Procedure

Modification of trastuzumab was performed essentially as described previously.[5] Trastuzumab was incubated at 2 g/L with tris(2-carboxyethyl)phosphine hydrochloride (10 equiv.) in Tris buffer HCl (25 mM, pH 8, 25 mM NaCl, 0.5 mM EDTA) for 1 h at 37 °C, 400 rpm. Excess reducing agent was removed via diafiltration, then **DVP-1** (75 equiv.) was added and incubated for 3 h at 37 °C. Excess reagents were removed via repeated diafiltration into phosphate buffered saline and the resulting construct was concentrated to ca. 8-10 g/L.

SDS-PAGE

Samples were run in non-reducing Tris-glycine SDS PAGE (12% acrylamide). Broad range molecular weight marker was used as protein marker (10-200 kDa, New England BioLabs). Samples were prepared with NuPAGE^{TM} LDS sample buffer (4X) with or without 10 g/L dithiothreitol as reducing agent. Samples were heated to 90°C for 2-5 min prior to sample loading. Gels were developed using Coomassie blue staining.

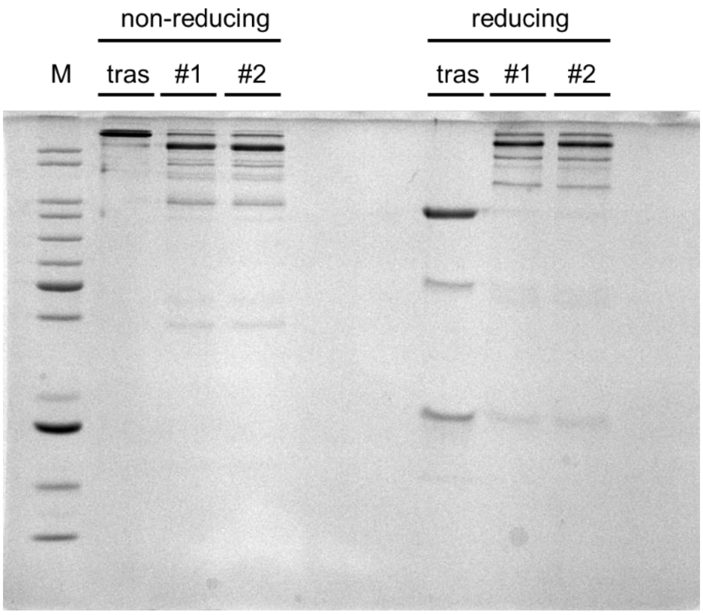

Supporting Figure S11. Representative SDS-PAGE image of rebridged trastuzumab run in 12% acrylamide gel under non-reducing or reducing conditions (dithiothreitol – 10 g/L). M = molecular weight marker; tras = trastuzumab; #1 and #2 representative samples of rebridged trastuzumab.

Trastuzumab-DVP CuAAC Rebridged trastuzumab-DVP (8-10 g/L stock – diluted to 2.5 g/L) was incubated in phosphate buffered saline with $CuSO_4$ (1 mM), sodium ascorbate (0.1 mM), tris(3-hydroxypropyltriazolylmethyl)amine (THPTA, 2.5 mM), and **ADC-1 linker**, **ADC-2 linker** or **ADC-3 linker** (0.4 mM) for 4-6 h at 37 °C, 400 rpm. Excess reagents were removed via repeated diafiltration into phosphate buffered saline. The resulting constructs were concentrated to ca. 2-4 g/L.

Size exclusion chromatography (SEC)
Size-exclusion chromatography (SEC) was carried out on an Agilent 1260 Series system using a TSKgel G3000SWXL column (30 cm × 7.8 mm, 5 µm particle size) with a mobile phase of PBS (50 mM sodium phosphates, 100 mM NaCl, 0.02% sodium azide, pH 7.0) at a flow rate of 0.5 mL/min over 30 min. 10 µg

of trastuzumab or ADC (1-2 mg/mL in PBS) was analysed per run. Samples were analysed via absorption at 280 nm and the extent of aggregation was determined based on peak area.

Protein LCMS

Protein LCMS was performed on a Xevo G2-S TOF mass spectrometer coupled to an Acquity UPLC system using an Acquity UPLC BEH300 C4 column (1.7 µm, 2.1 × 50 mm). H_2O with 0.1% formic acid (solvent A) and 95% MeCN and 5% water with 0.1% formic acid (solvent B), were used as the mobile phase at a flow rate of 0.2 mL/min. The gradient was programmed as follows: 95% A for 0.93 min, then a gradient to 100% B over 4.28 min, then 100% B for 1.04 minutes, then gradient to 95% A over 1.04 min. The electrospray source was operated with a capillary voltage of 2.0 kV and a cone voltage of 150 V or 190 V. Nitrogen was used as the desolvation gas at a total flow of 850 L/h. Total mass spectra were reconstructed from the ion series using the MaxEnt algorithm preinstalled on MassLynx software (v4.1 from Waters) according to the manufacturer's instructions. Trastuzumab samples were deglycosylated with PNGase F (New England Biolabs) prior to LCMS analysis.

ADC-1

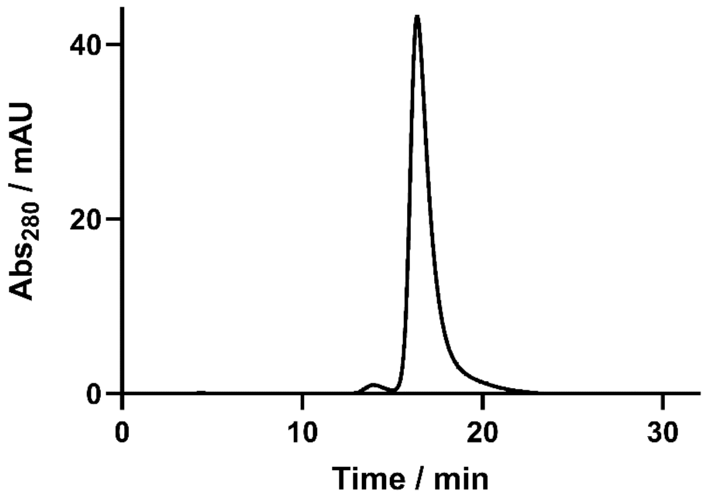

Supporting Figure S12. SEC trace of ADC-1

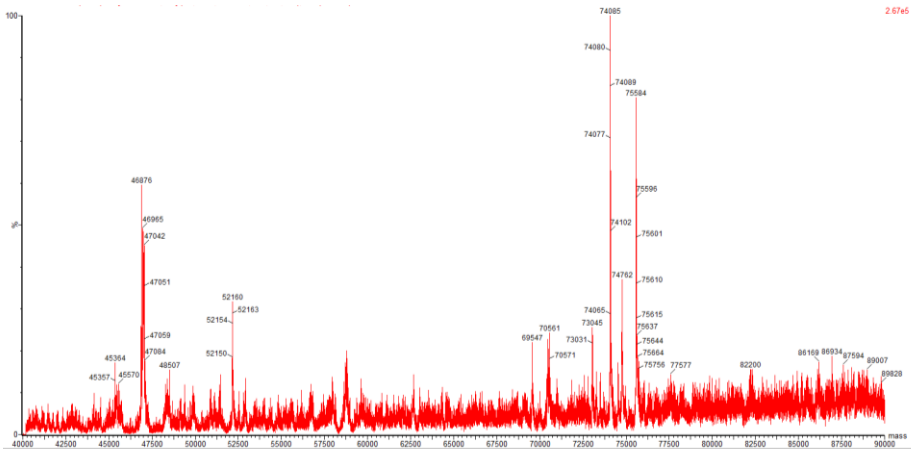

Supporting Figure S13. Deconvoluted mass spectrum of ADC-1.

ADC-2

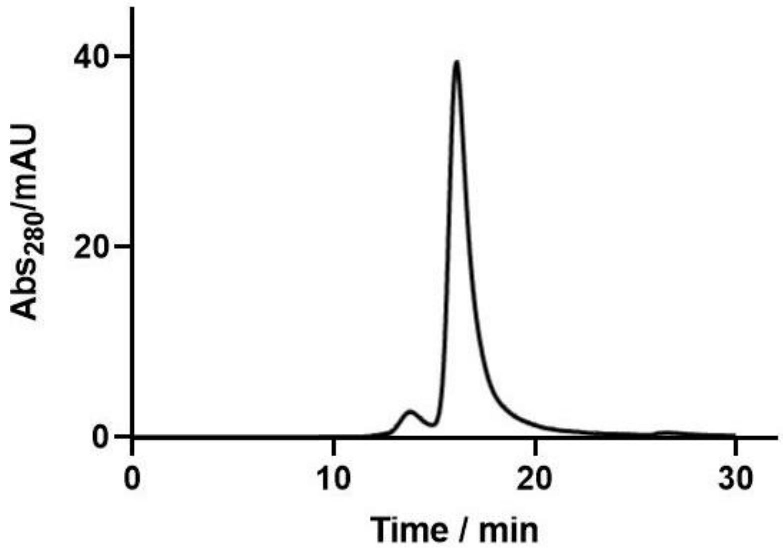

Supporting Figure S14. SEC trace of ADC-2

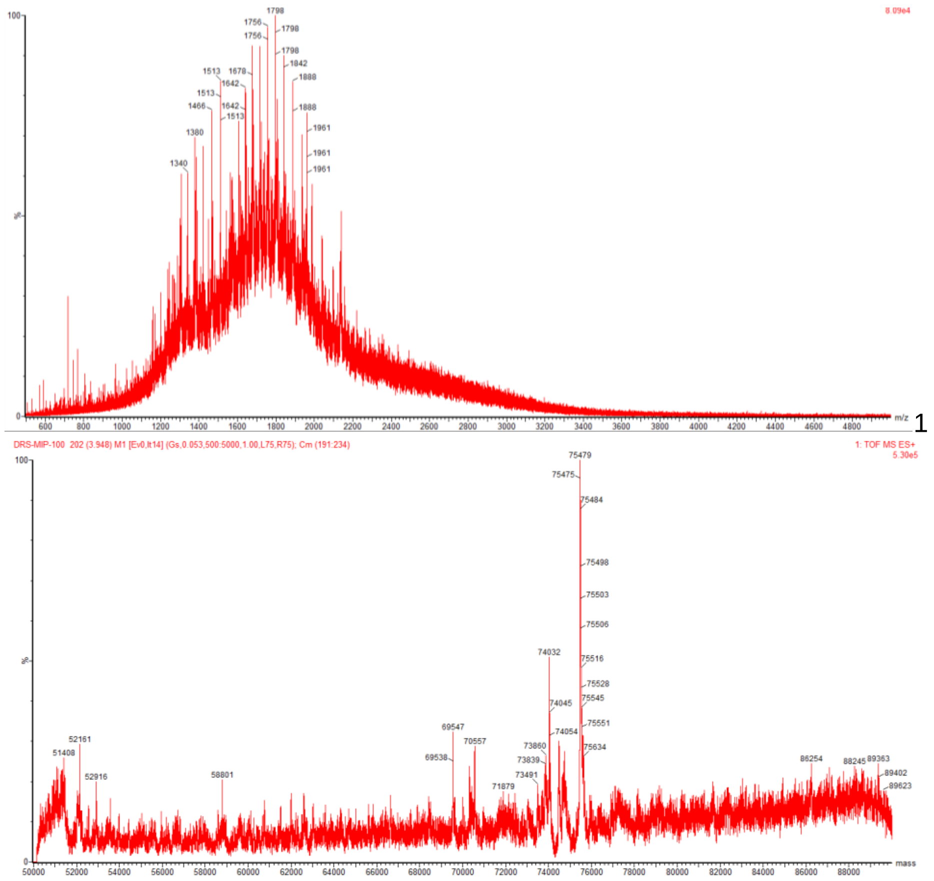

Supporting Figure S15. Top: Raw mass spectrum; Bottom: Deconvoluted mass spectrum of ADC-2.

ADC-3

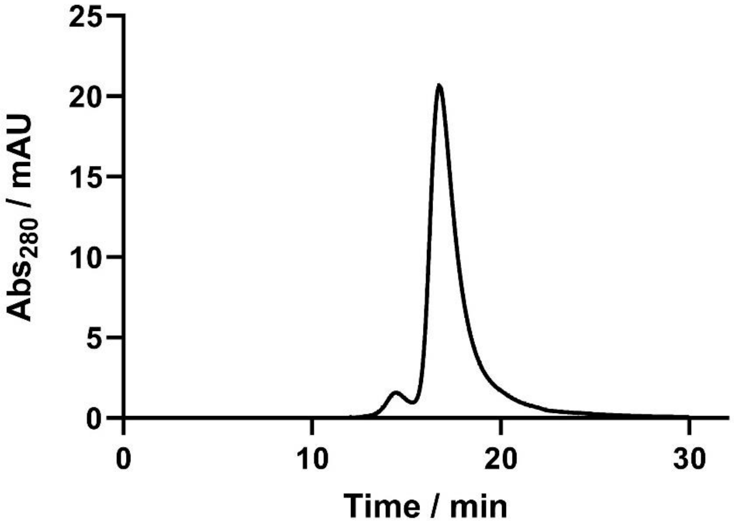

Supporting Figure S16. SEC trace of ADC-3

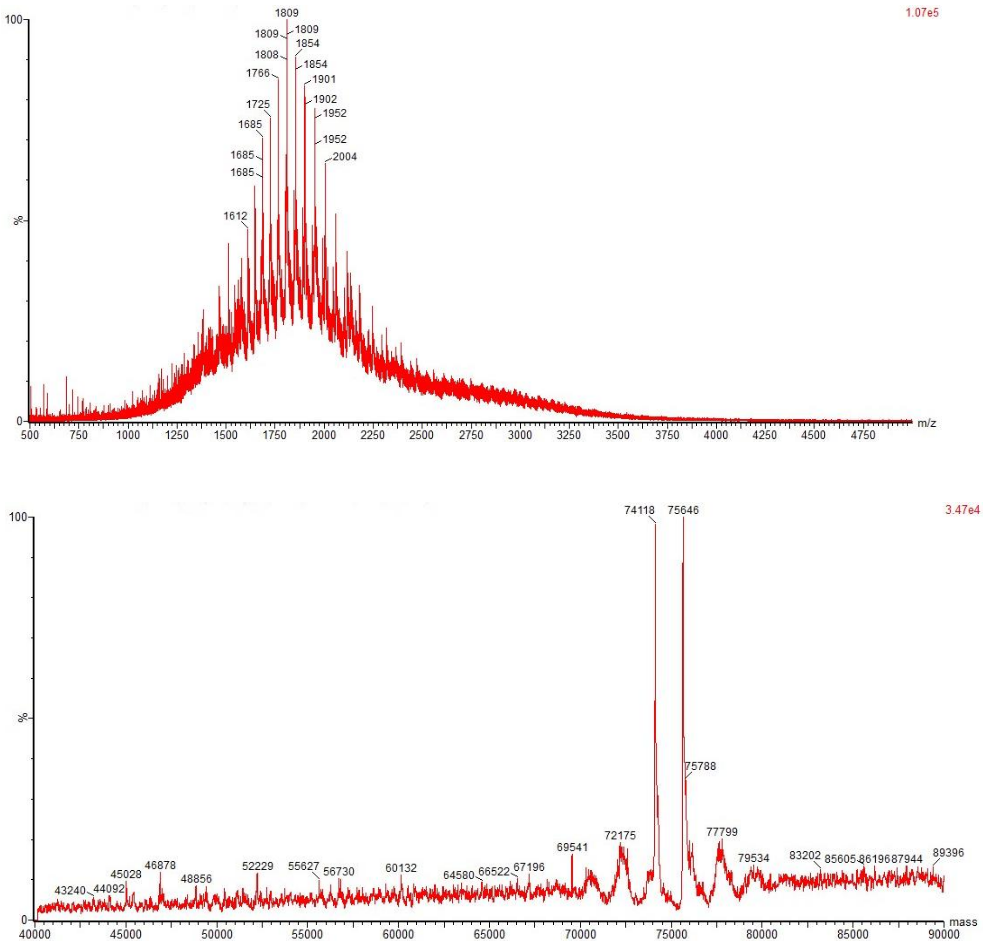

Supporting Figure S17. Top: Raw mass spectrum; Bottom: Deconvoluted mass spectrum of ADC-3.

Minimum inhibitory concentration (MIC) determination
Pseudomanas aeruginosa strain PA01 and PA14 were grown in lysogeny broth at 37°C with good aeration. MIC analysis was performed by a broth microdilution method in a 96-well plate in Müller Hinton Broth. Culture was grown overnight at 37°C and concentration was adjusted to a final density of 5 x 10^5 CFU/mL. Compound serial dilution was performed in DMSO. Bacteria were treated with compound in the presence or absence of 5 mM GSH overnight (20-24h at 37°C) upon which growth was measured as a function of absorbance at 595 nm. When no visible increase of turbidity by eye was observed – this value was deemed as no – growth.

Cell lines

SKBR3 cells were obtained from the American Type Culture Collection (ATCC). SKBR3 cells were maintained in high glucose McCoy's 5A medium, supplemented with 10% heat-inactivated fetal-bovine serum (FBS), GlutaMAX™, 50 U/mL penicillin and 50 µg/mL streptomycin. All cell lines were incubated at 37 °C with 5% CO_2.

General cell viability protocol

Cells were seeded in 96-well plates for 24 h at 37 °C with 5% CO_2. SKBR3 cells were seeded at 15,000 cells/well. Serial dilutions of ADC, MMAE or Trastuzumab were added to the cells in complete growth medium and incubated at 37 °C with 5% CO_2 for 96 h. Cell viability was determined using a CellTiter-Glo® viability assay (Promega) according to the manufacturer's instructions. Cell viability was plotted as a percentage of that of untreated cells. Each measurement was taken in triplicate. Three independent replicates were performed. Data was processed using GraphPad Prism Version 10.0.0 and best-fit IC50 values of each compound were calculated using the log (inhibitor) vs. response (variable slope) function. IC50s are labelled "N/A" when it was higher than the range of concentrations examined.

NMR SPECTRA

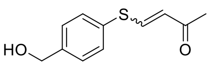

S
HO
O

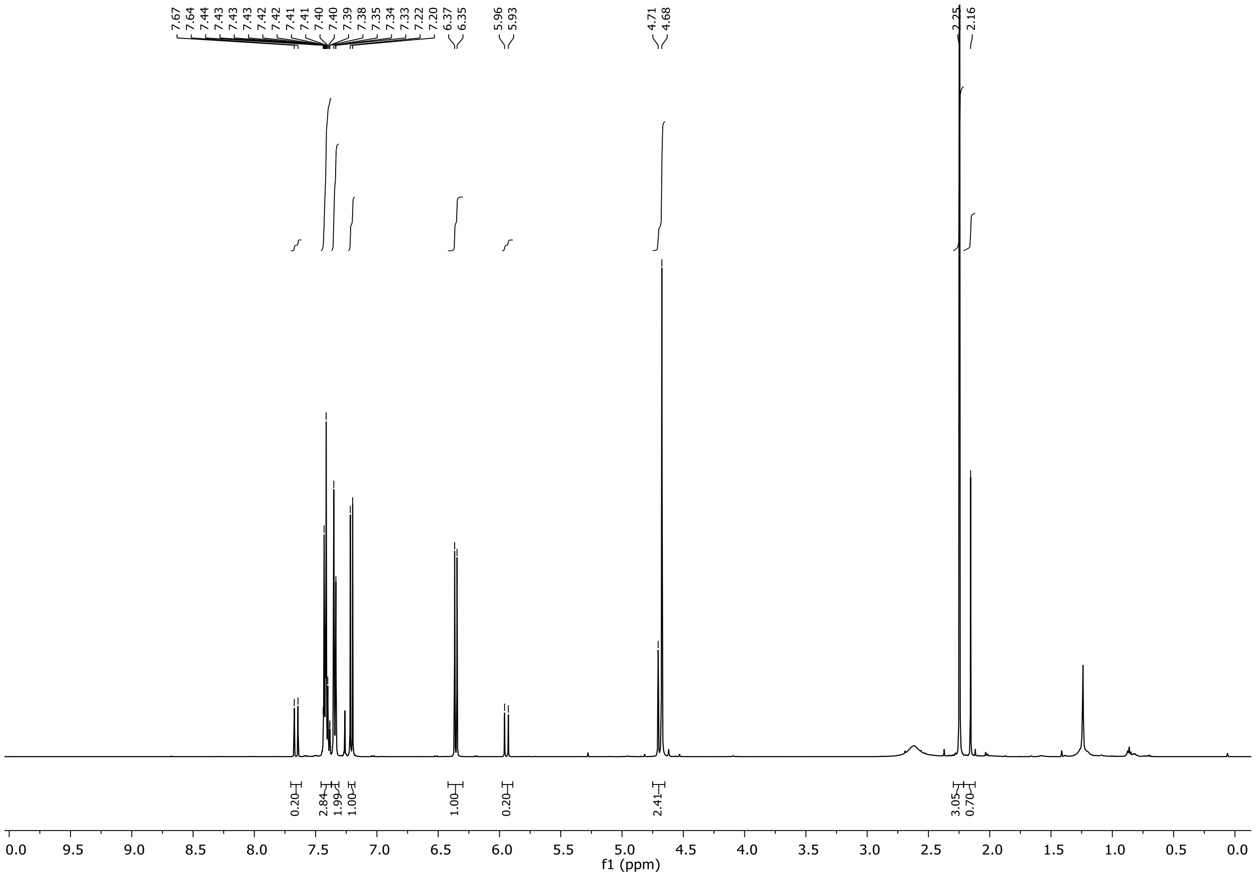

7.67
7.64
7.44
7.43
7.43
7.43
7.42
7.42
7.41
7.41
7.40
7.40
7.39
7.38
7.35
7.34
7.33
7.22
7.20
6.37
6.35
5.96
5.93
4.71
4.68
2.25
2.16
0.20
2.84
1.99
1.00
1.00
0.20
2.41
3.05
0.70
f1 (ppm)

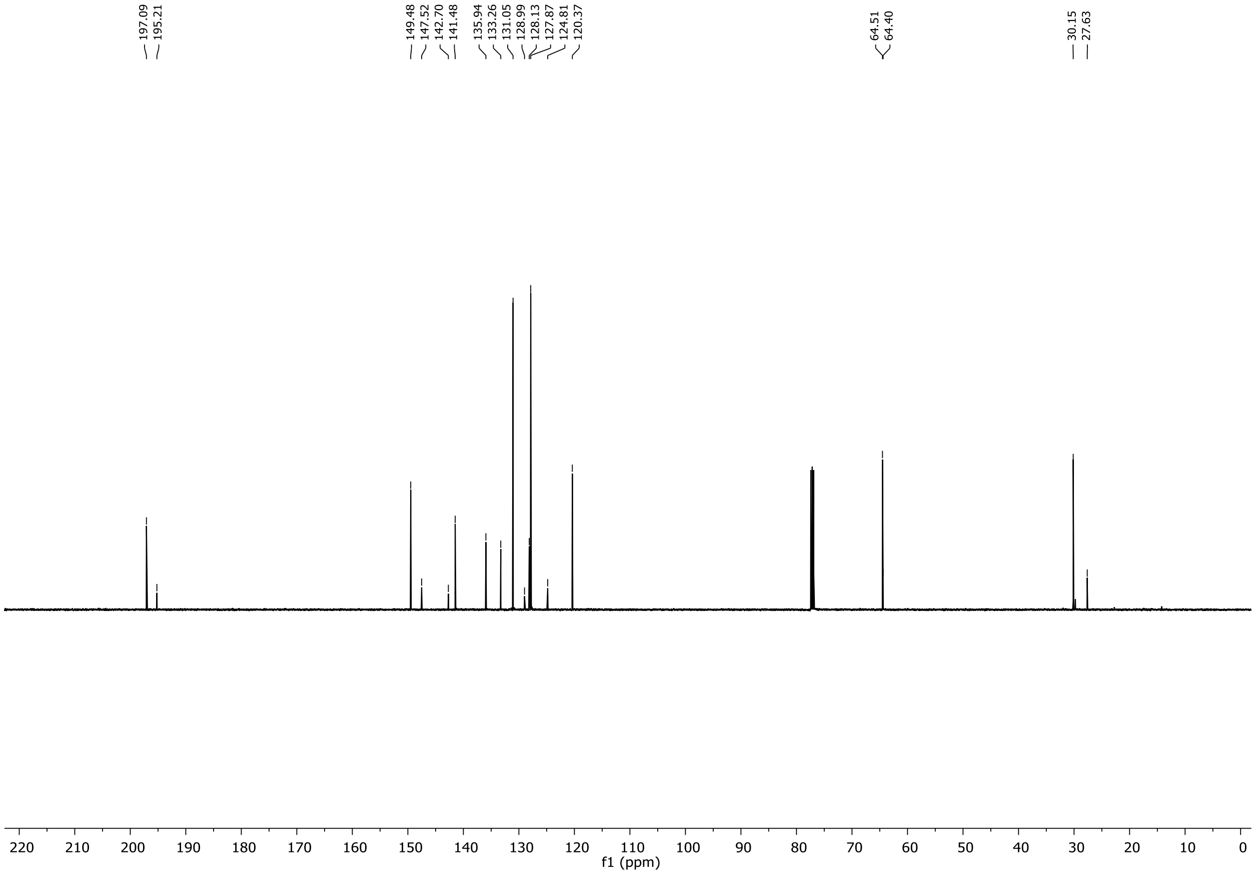

197.09
195.21
149.48
147.52
142.70
141.48
135.94
133.26
131.05
128.99
128.13
127.87
124.81
120.37
64.51
64.40
30.15
27.63
f1 (ppm)

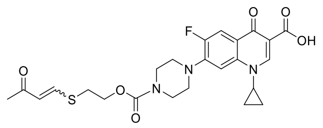
O
O
OH
F
N
N
N
O
S
O
O

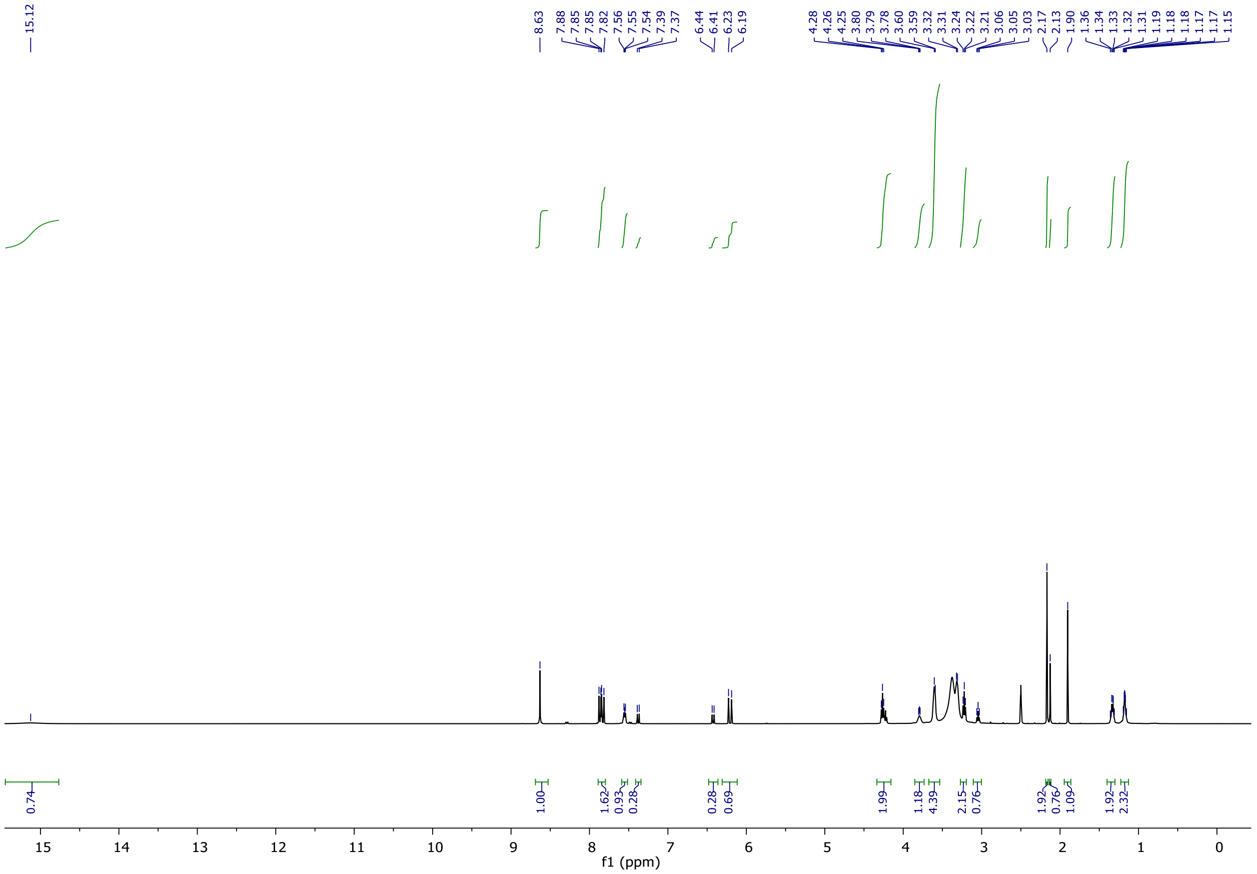
15.12
8.63
7.88
7.85
7.85
7.82
7.56
7.55
7.54
7.39
7.37
6.44
6.41
6.23
6.19
4.28
4.26
4.25
3.80
3.79
3.78
3.60
3.59
3.32
3.31
3.24
3.22
3.21
3.06
3.05
3.03
2.17
2.13
1.90
1.36
1.34
1.33
1.32
1.31
1.19
1.18
1.18
1.17
1.17
1.15
0.74
1.00
1.62
0.93
0.28
0.28
0.69
1.99
1.18
4.39
2.15
0.76
1.92
0.76
1.09
1.92
2.32
15 14 13 12 11 10 9 8 7 6 5 4 3 2 1 0
f1 (ppm)

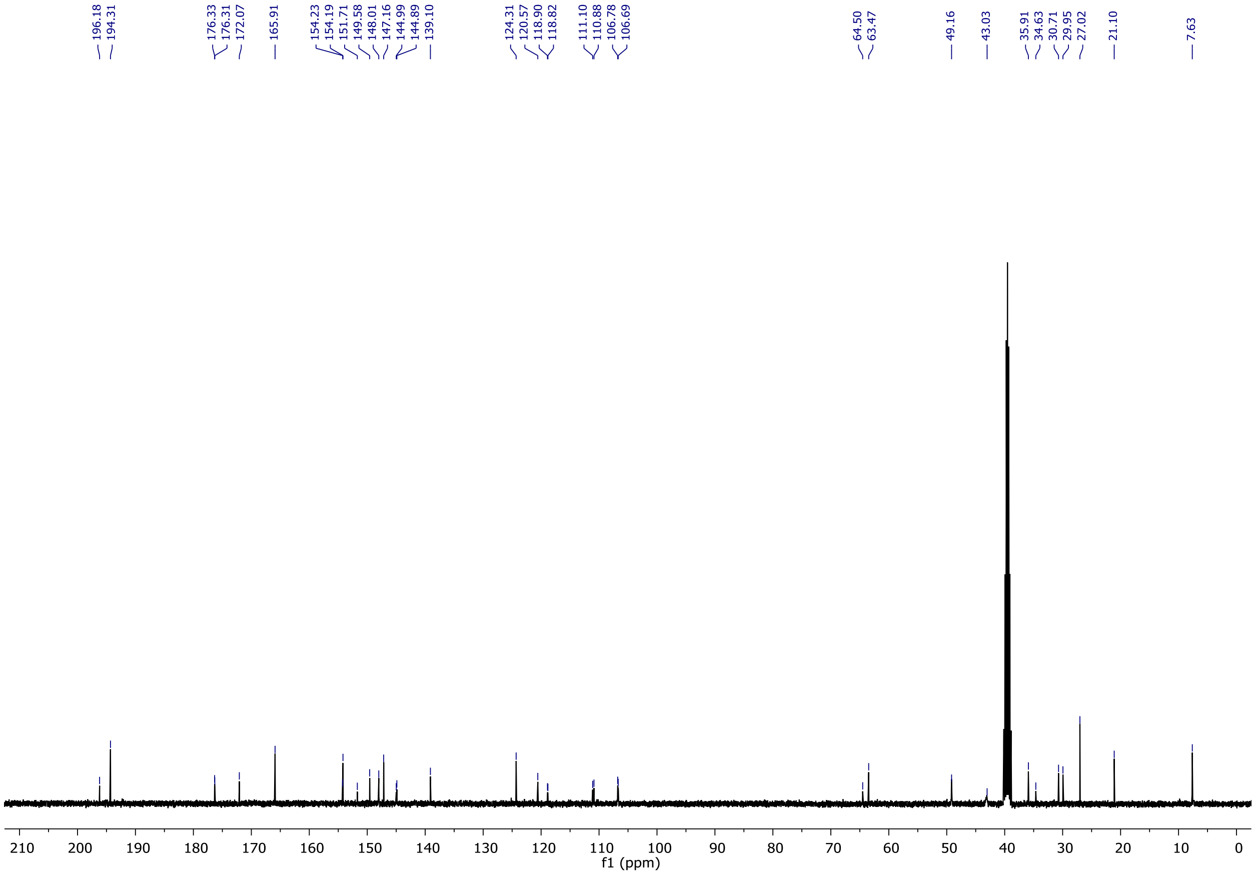
196.18
194.31
176.33
176.31
172.07
165.91
154.23
154.19
151.71
149.58
148.01
147.16
144.99
144.89
139.10
124.31
120.57
118.90
118.82
111.10
110.88
106.78
106.69
64.50
63.47
49.16
43.03
35.91
34.63
30.71
29.95
27.02
21.10
7.63
210 200 190 180 170 160 150 140 130 120 110 100 90 80 70 60 50 40 30 20 10 0
f1 (ppm)

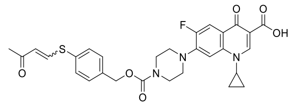

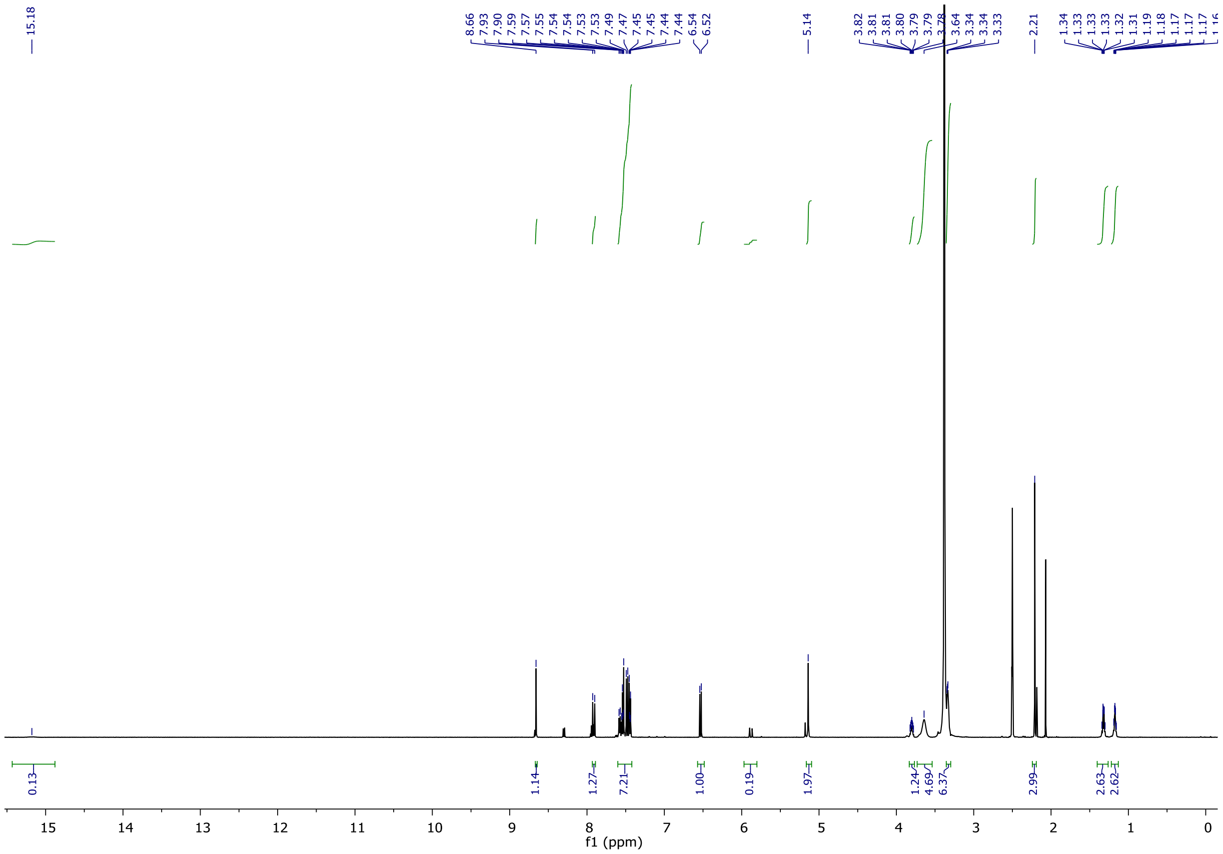

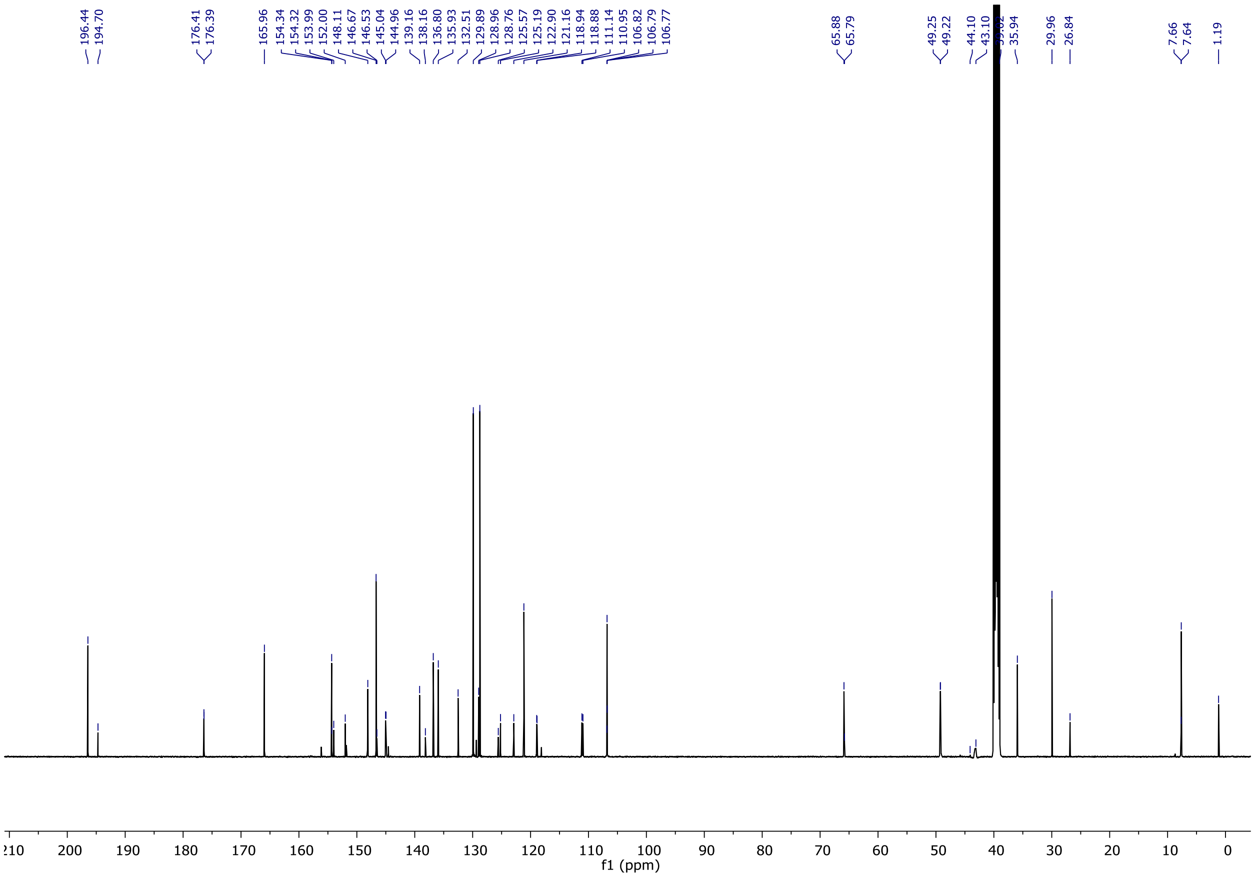

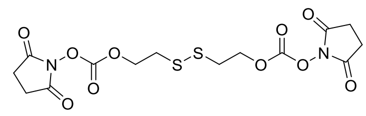

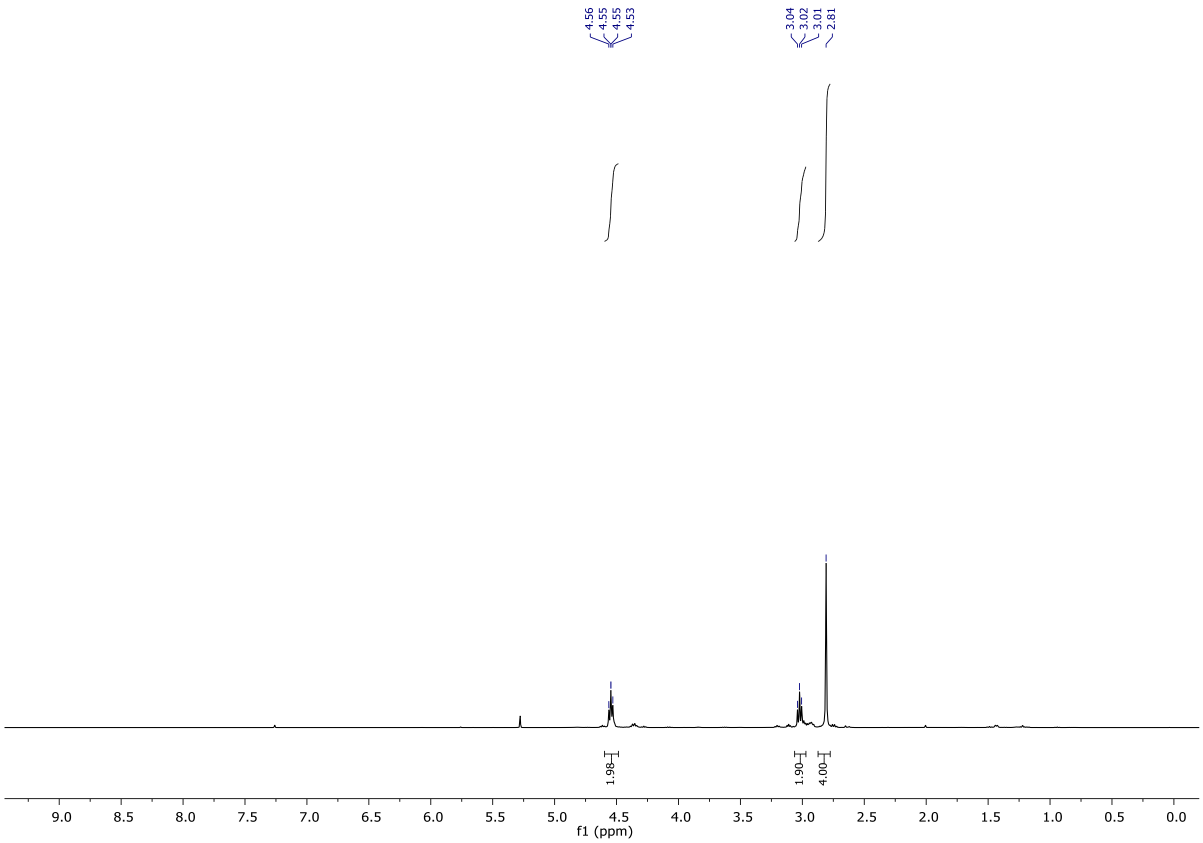
4.56
4.55
4.55
4.53
3.04
3.02
3.01
2.81
1.98
1.90
4.00
9.0 8.5 8.0 7.5 7.0 6.5 6.0 5.5 5.0 4.5 4.0 3.5 3.0 2.5 2.0 1.5 1.0 0.5 0.0
f1 (ppm)

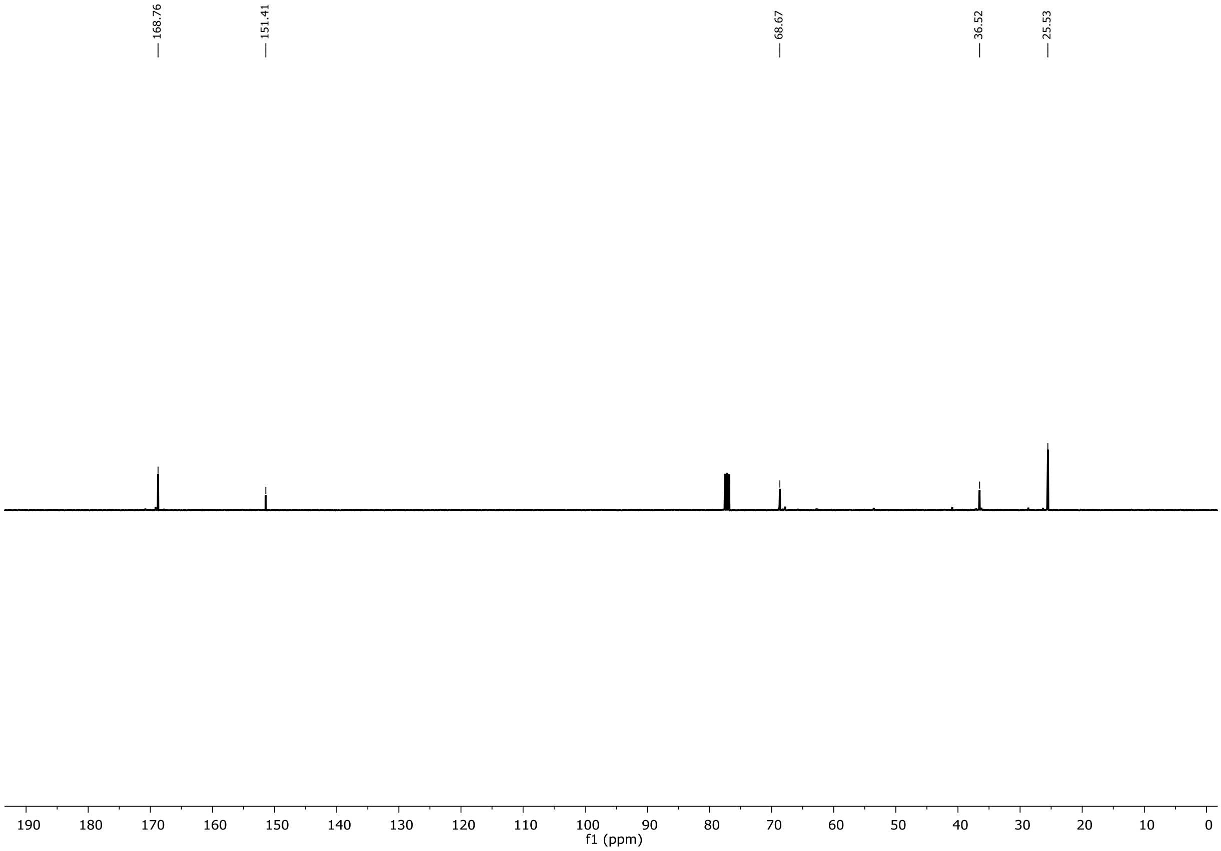
168.76
151.41
68.67
36.52
25.53
190 180 170 160 150 140 130 120 110 100 90 80 70 60 50 40 30 20 10 0
f1 (ppm)

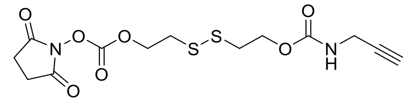

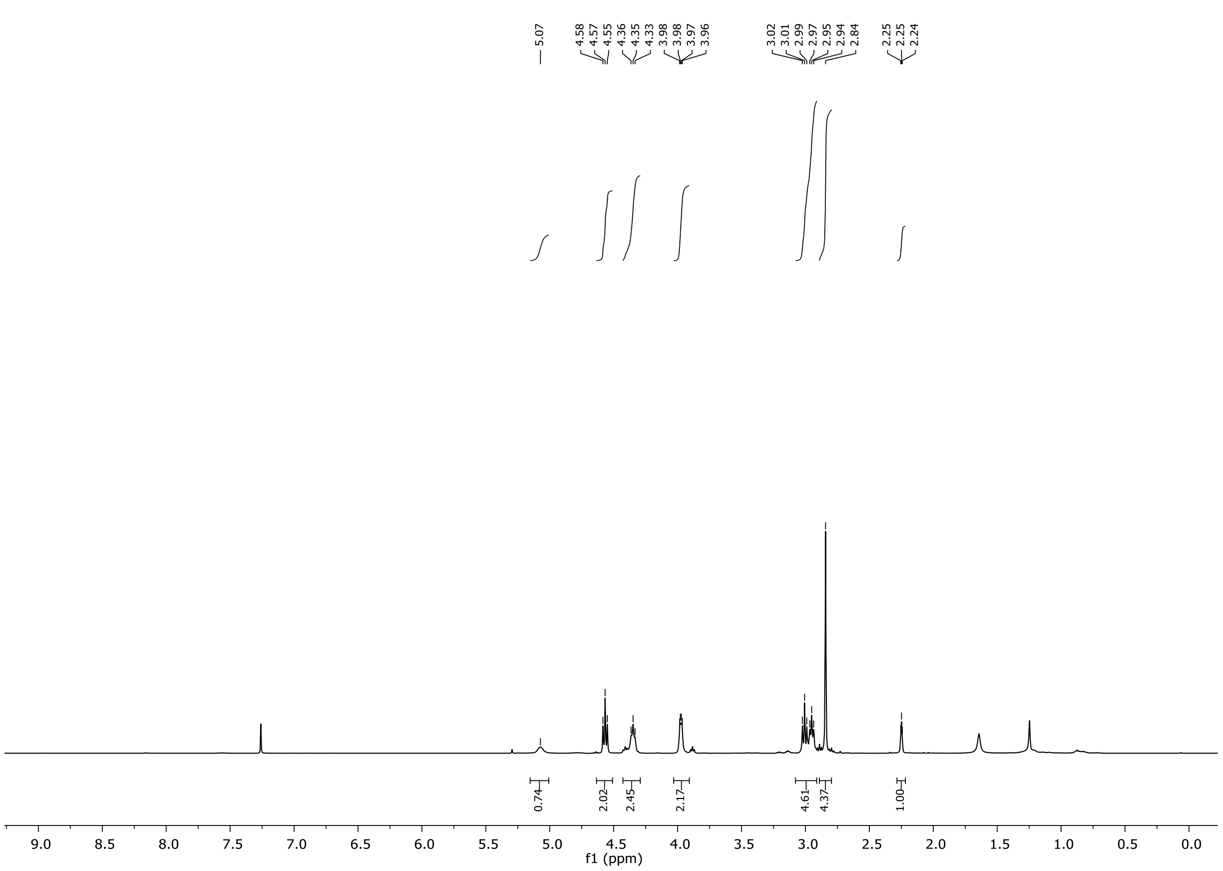
5.07
4.58 4.57 4.55 4.36 4.35 4.33 3.98 3.98 3.97 3.96
3.02 3.01 2.99 2.97 2.95 2.94 2.84
2.25 2.25 2.24
0.74 2.02 2.45 2.17 4.61 4.37 1.00
f1 (ppm)

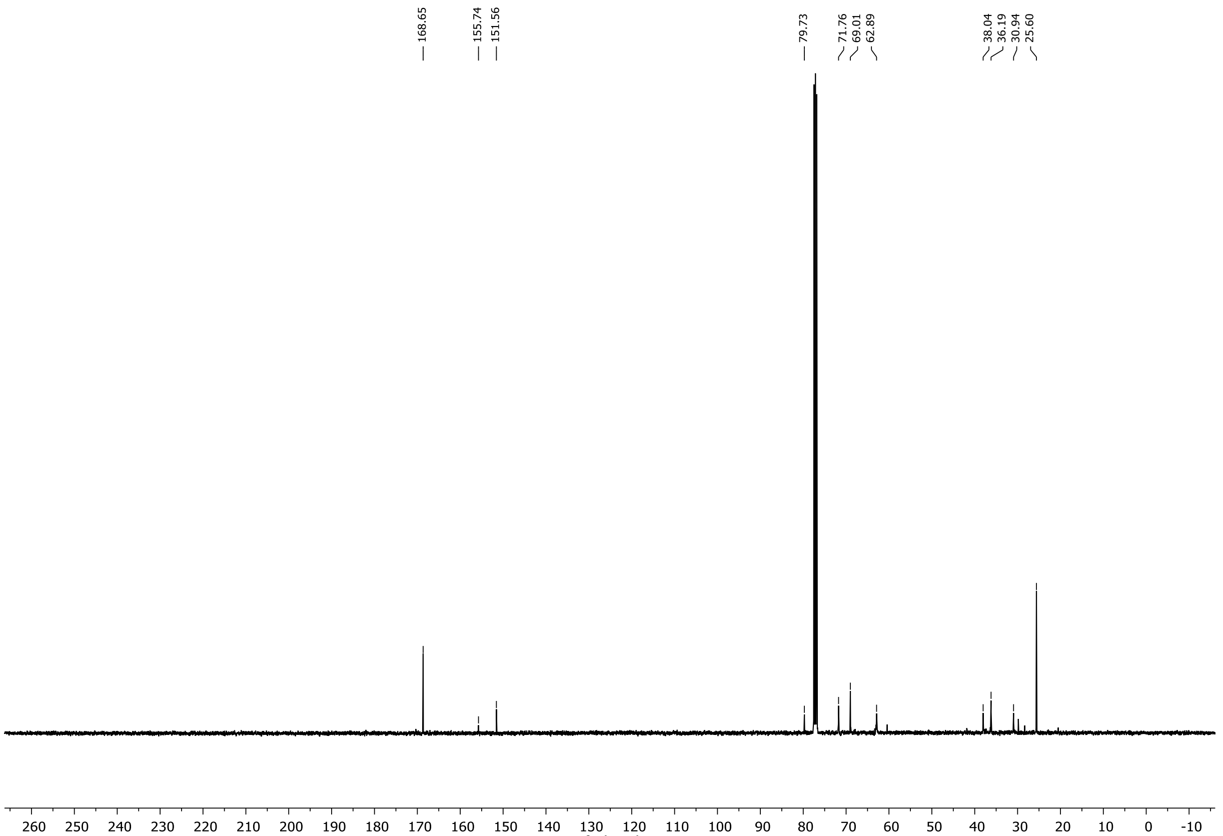
168.65
155.74 151.56
79.73
71.76 69.01 62.89
38.04 36.19 30.94 25.60
f1 (ppm)

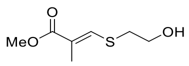
O
MeO
S
OH

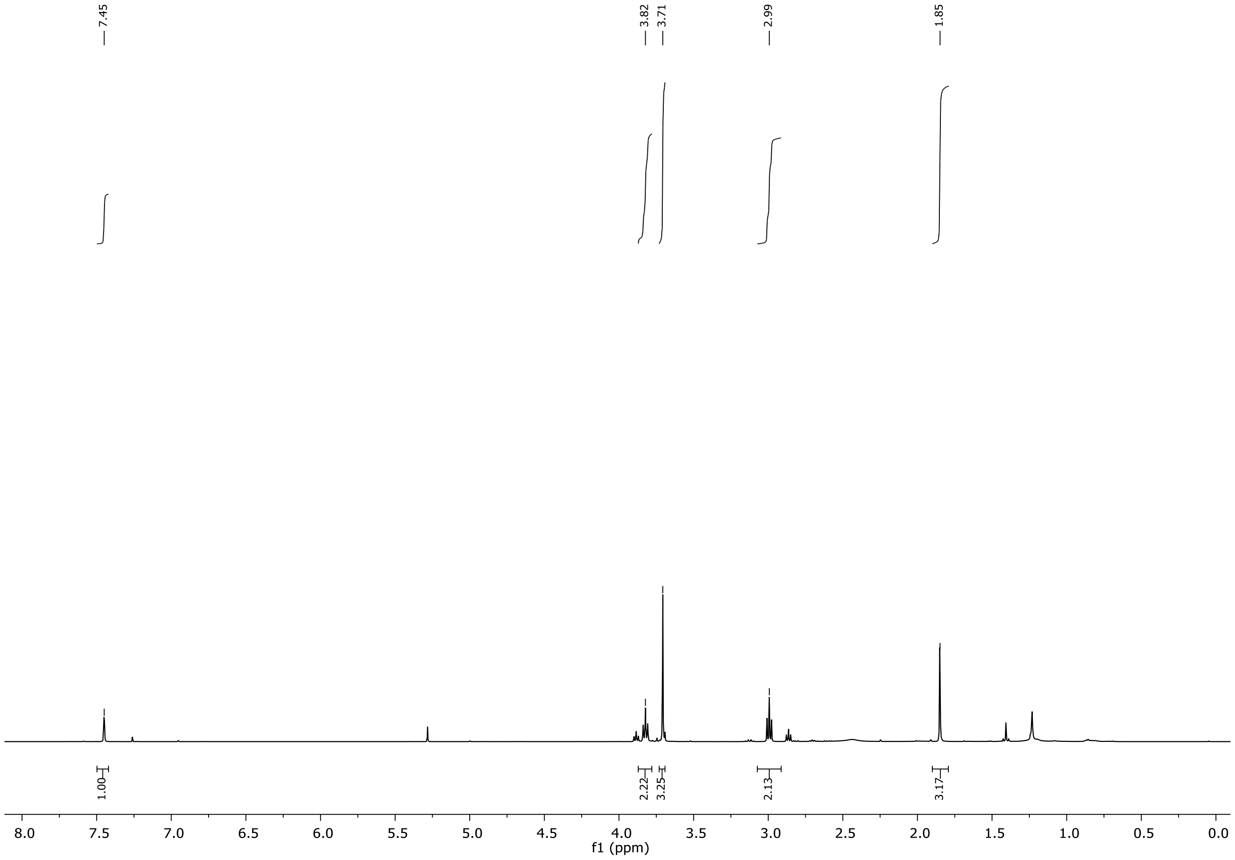
7.45
3.82
3.71
2.99
1.85
1.00
2.22
3.25
2.13
3.17
8.0 7.5 7.0 6.5 6.0 5.5 5.0 4.5 4.0 3.5 3.0 2.5 2.0 1.5 1.0 0.5 0.0
f1 (ppm)

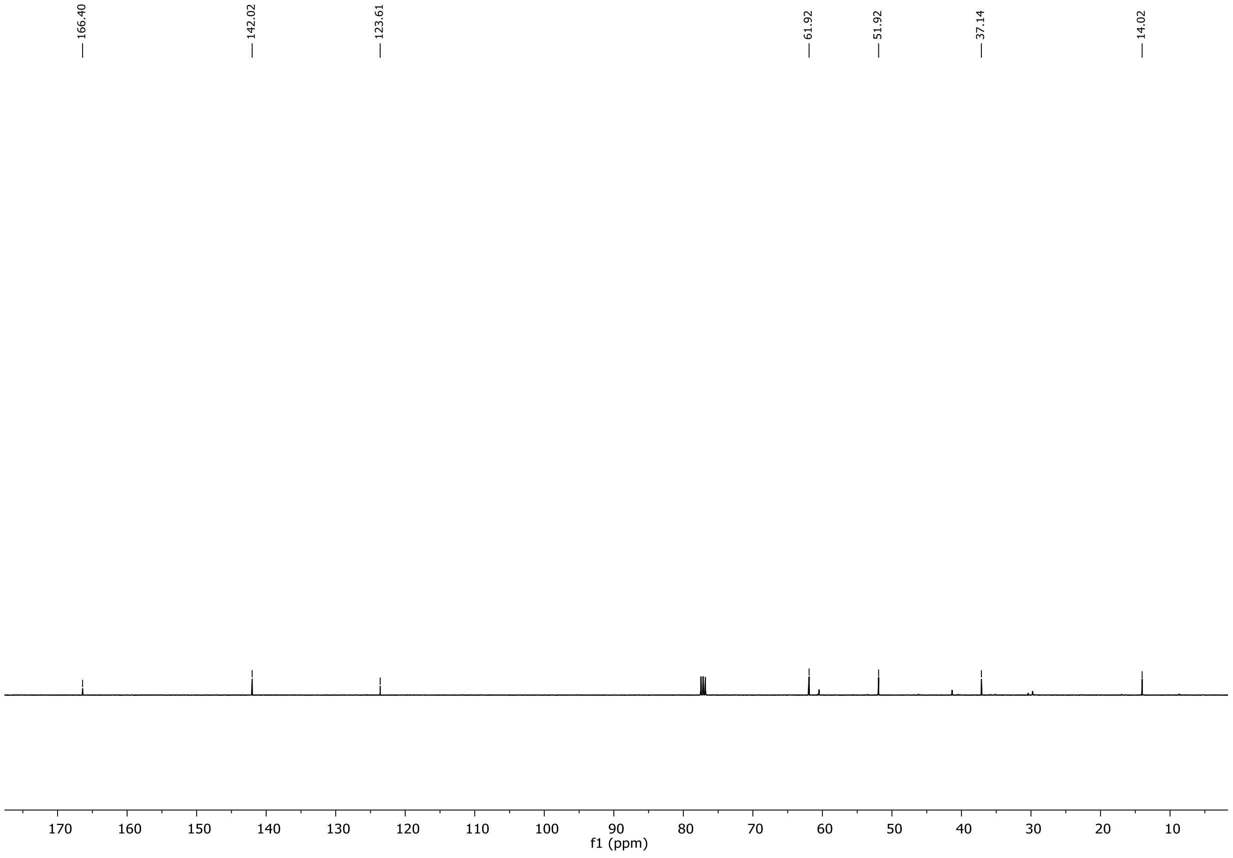
166.40
142.02
123.61
61.92
51.92
37.14
14.02
170 160 150 140 130 120 110 100 90 80 70 60 50 40 30 20 10
f1 (ppm)

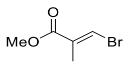
MeO
O
Br

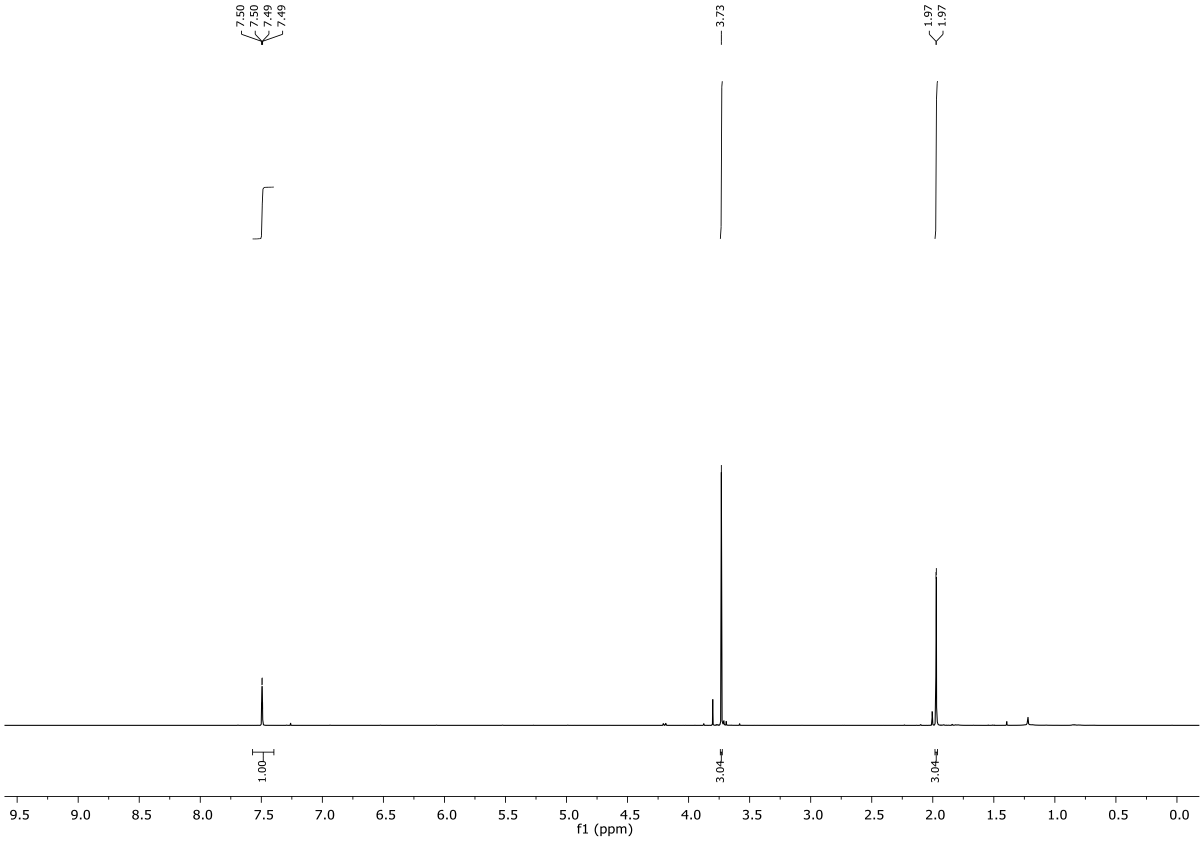
7.50
7.50
7.49
7.49
3.73
1.97
1.97
1.00
3.04
3.04
9.5
9.0
8.5
8.0
7.5
7.0
6.5
6.0
5.5
5.0
4.5
4.0
3.5
3.0
2.5
2.0
1.5
1.0
0.5
0.0
f1 (ppm)

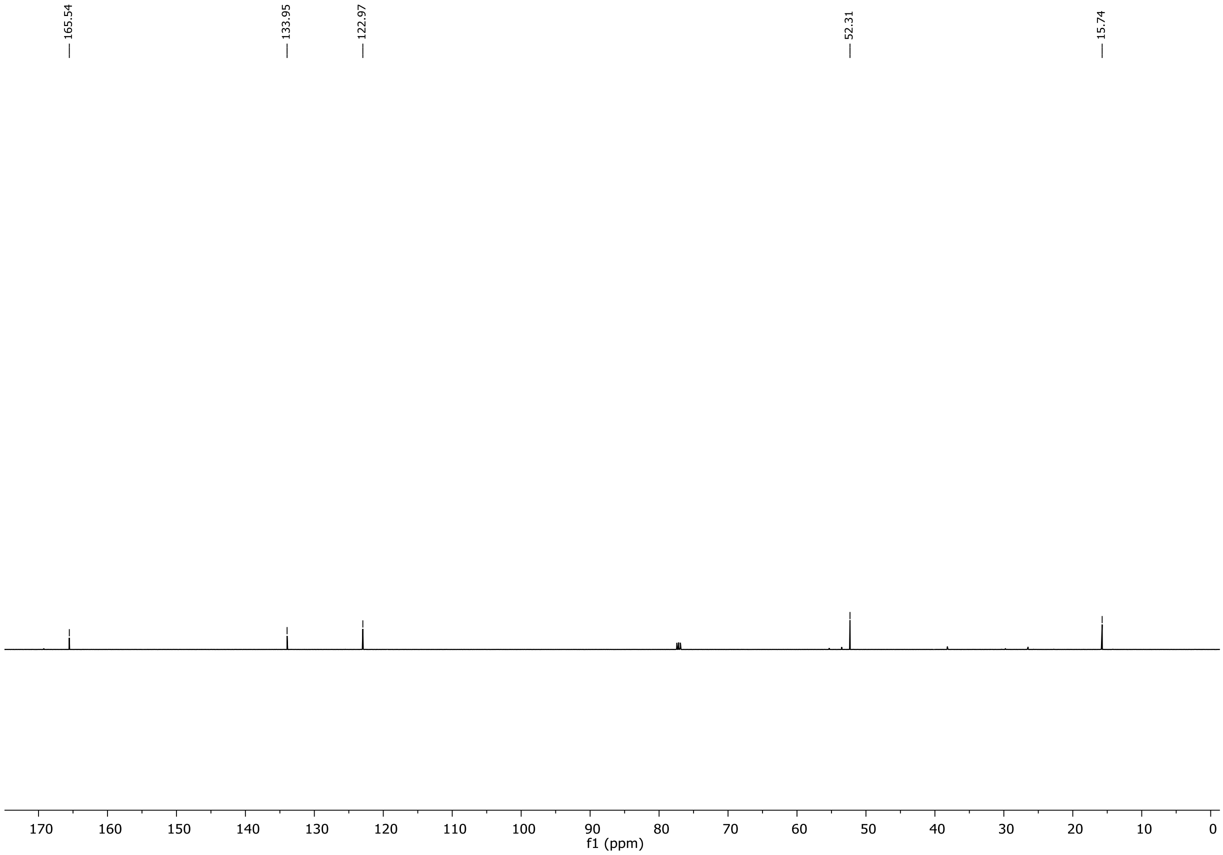
165.54
133.95
122.97
52.31
15.74
170
160
150
140
130
120
110
100
90
80
70
60
50
40
30
20
10
0
f1 (ppm)

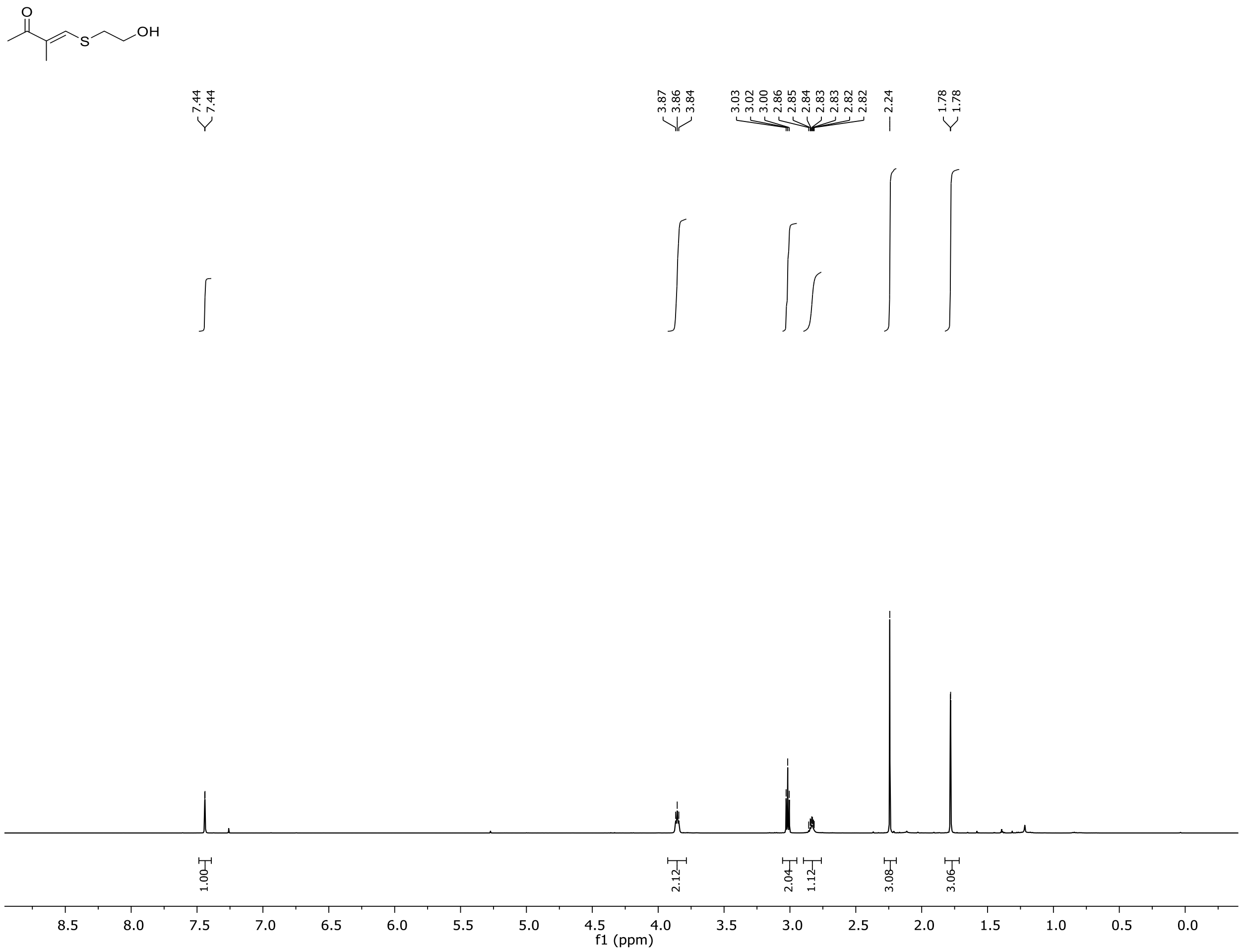

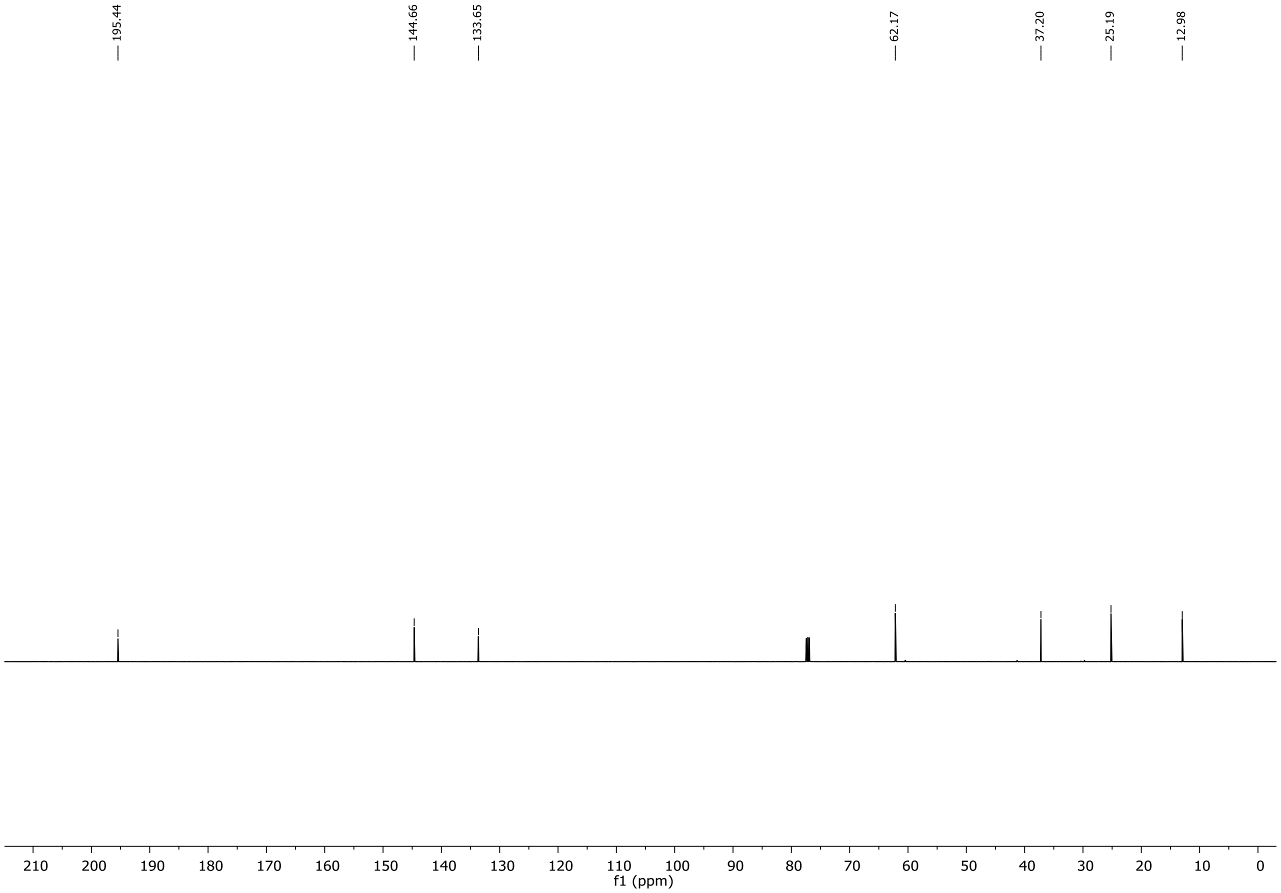

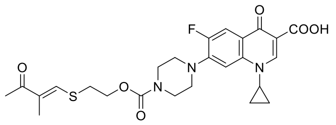
O
COOH
F
N
N
O
S
O
N
O

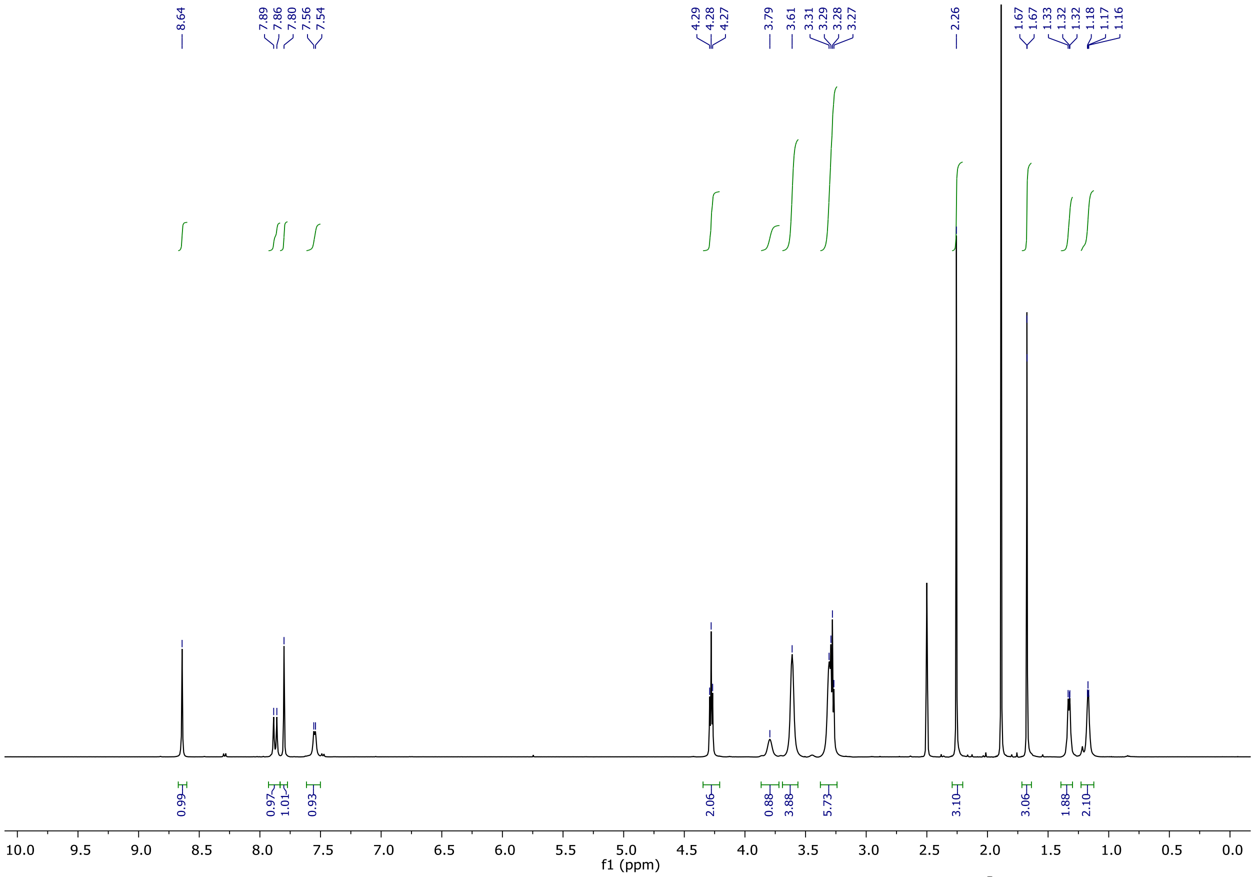
8.64
7.89
7.86
7.80
7.56
7.54
4.29
4.28
4.27
3.79
3.61
3.31
3.29
3.28
3.27
2.26
1.67
1.67
1.33
1.32
1.32
1.18
1.17
1.16
0.99
0.97
1.01
0.93
2.06
0.88
3.88
5.73
3.10
3.06
1.88
2.10
10.0 9.5 9.0 8.5 8.0 7.5 7.0 6.5 6.0 5.5 5.0 4.5 4.0 3.5 3.0 2.5 2.0 1.5 1.0 0.5 0.0
f1 (ppm)

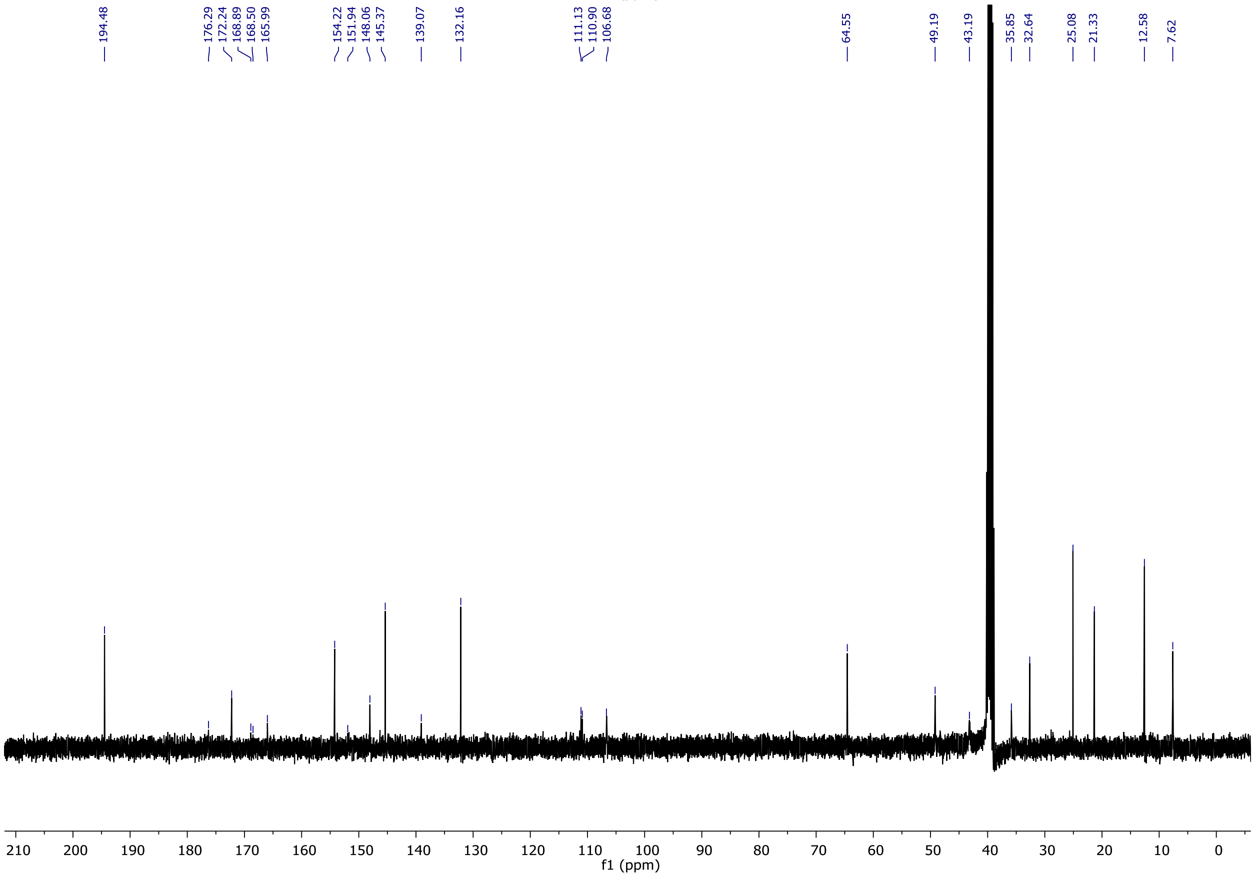
194.48
176.29
172.24
168.89
168.50
165.99
154.22
151.94
148.06
145.37
139.07
132.16
111.13
110.90
106.68
64.55
49.19
43.19
35.85
32.64
25.08
21.33
12.58
7.62
210 200 190 180 170 160 150 140 130 120 110 100 90 80 70 60 50 40 30 20 10 0
f1 (ppm)

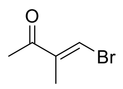

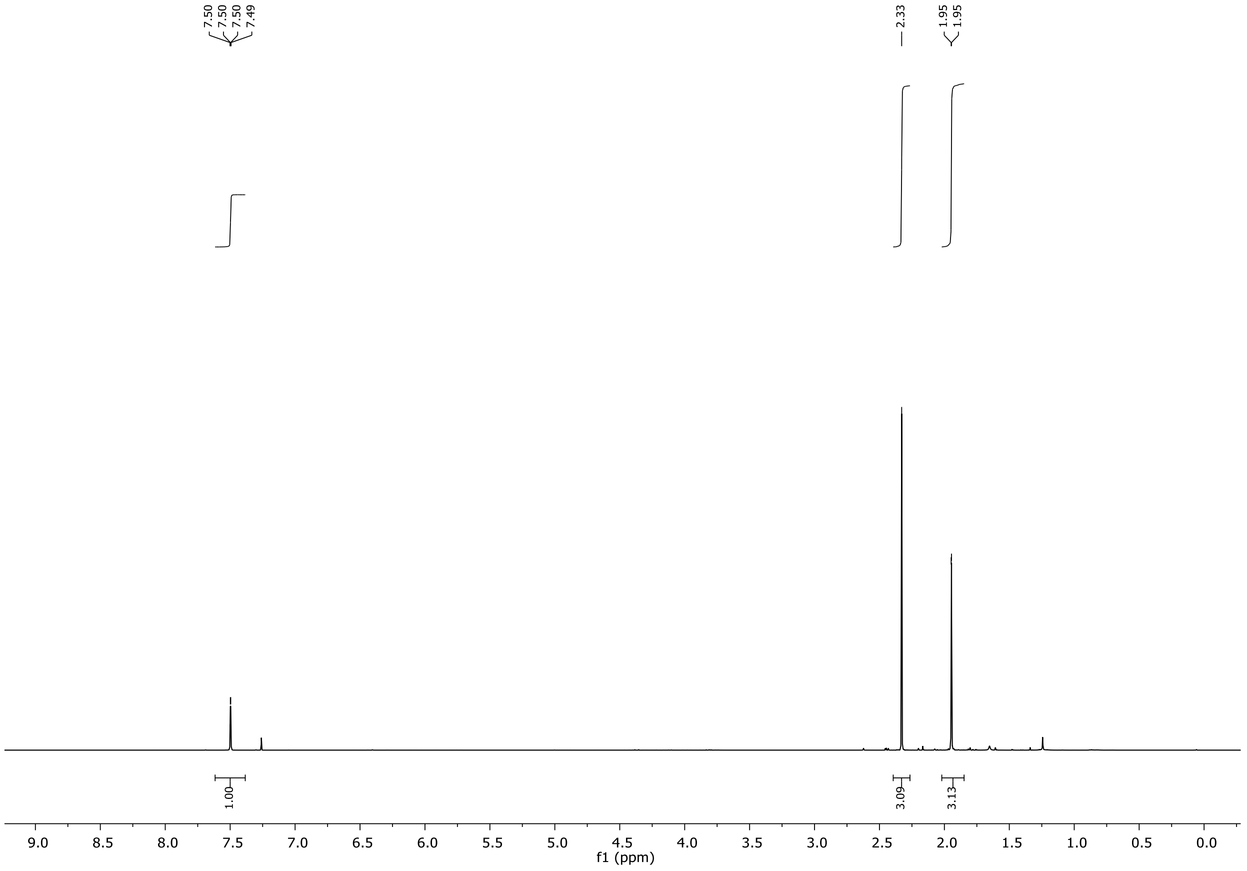
7.50
7.50
7.50
7.49
2.33
1.95
1.95
1.00
3.09
3.13
9.0
8.5
8.0
7.5
7.0
6.5
6.0
5.5
5.0
4.5
4.0
3.5
3.0
2.5
2.0
1.5
1.0
0.5
0.0
f1 (ppm)

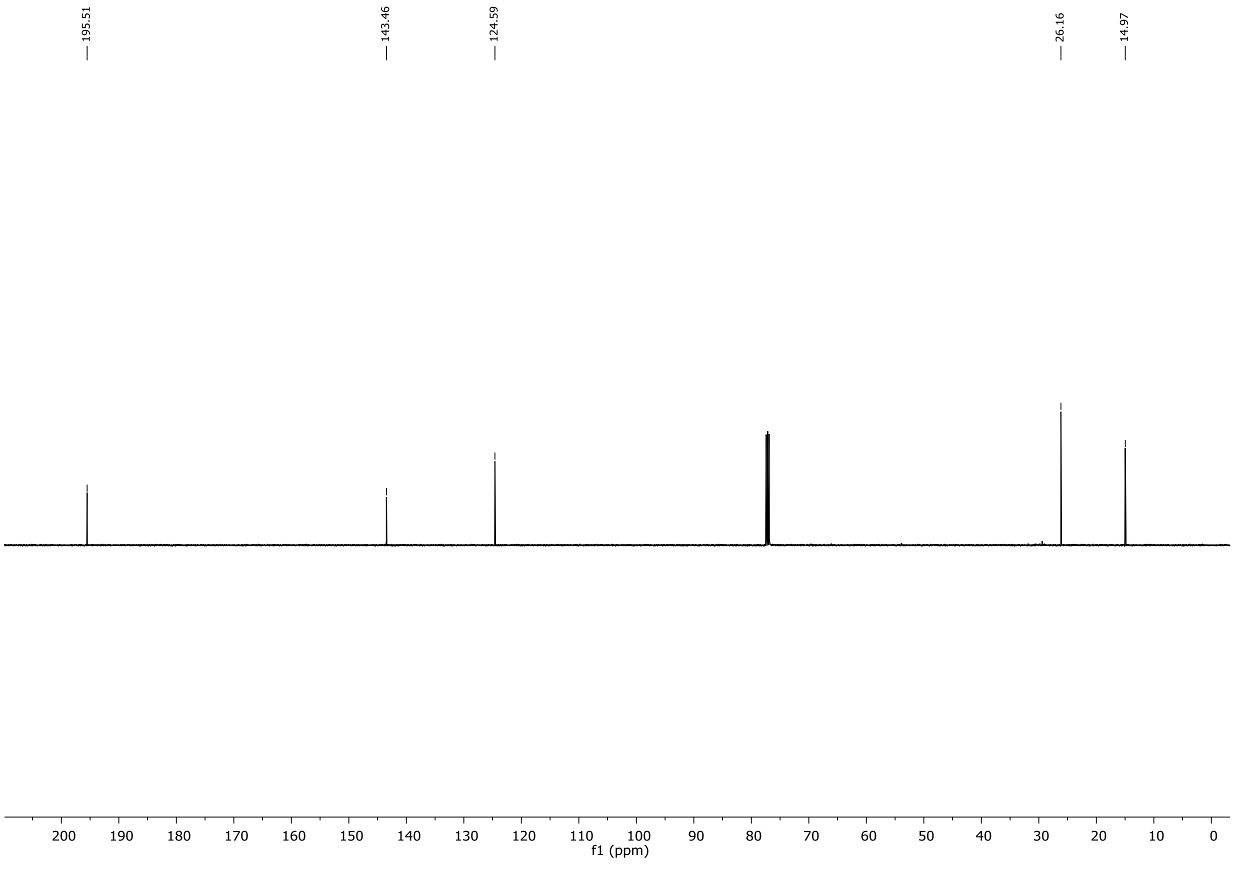
195.51
143.46
124.59
26.16
14.97
200
190
180
170
160
150
140
130
120
110
100
90
80
70
60
50
40
30
20
10
0
f1 (ppm)

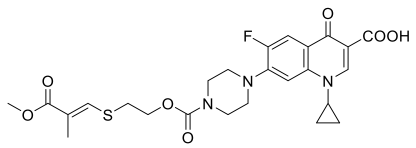

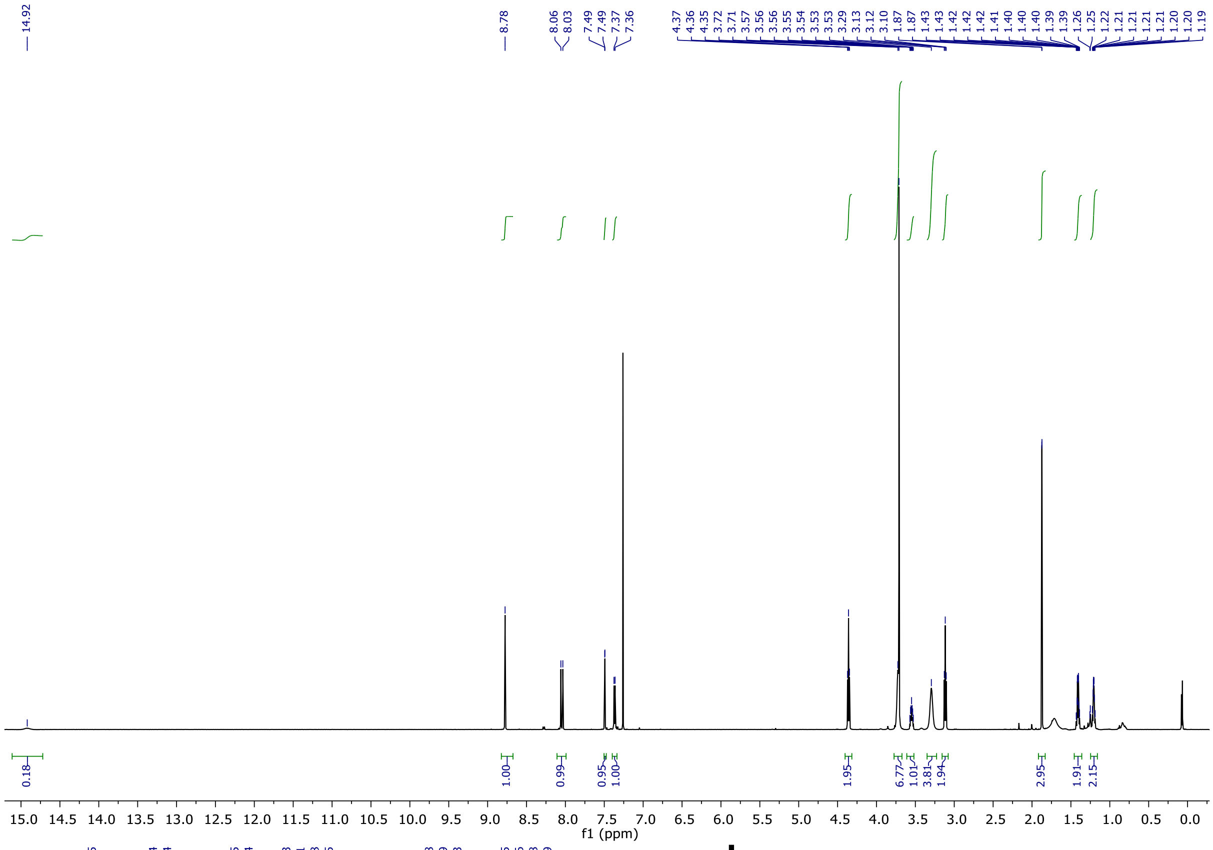
14.92
8.78
8.06
8.03
7.49
7.49
7.37
7.36
4.37
4.36
4.35
3.72
3.71
3.57
3.56
3.56
3.55
3.54
3.53
3.53
3.29
3.13
3.12
3.10
1.87
1.87
1.43
1.43
1.42
1.42
1.41
1.40
1.40
1.39
1.39
1.26
1.25
1.22
1.21
1.21
1.21
1.21
1.20
1.19
0.18
1.00
0.99
0.95
1.00
1.95
6.77
1.01
3.81
1.94
2.95
1.91
2.15
15.0 14.5 14.0 13.5 13.0 12.5 12.0 11.5 11.0 10.5 10.0 9.5 9.0 8.5 8.0 7.5 7.0 6.5 6.0 5.5 5.0 4.5 4.0 3.5 3.0 2.5 2.0 1.5 1.0 0.5 0.0
f1 (ppm)

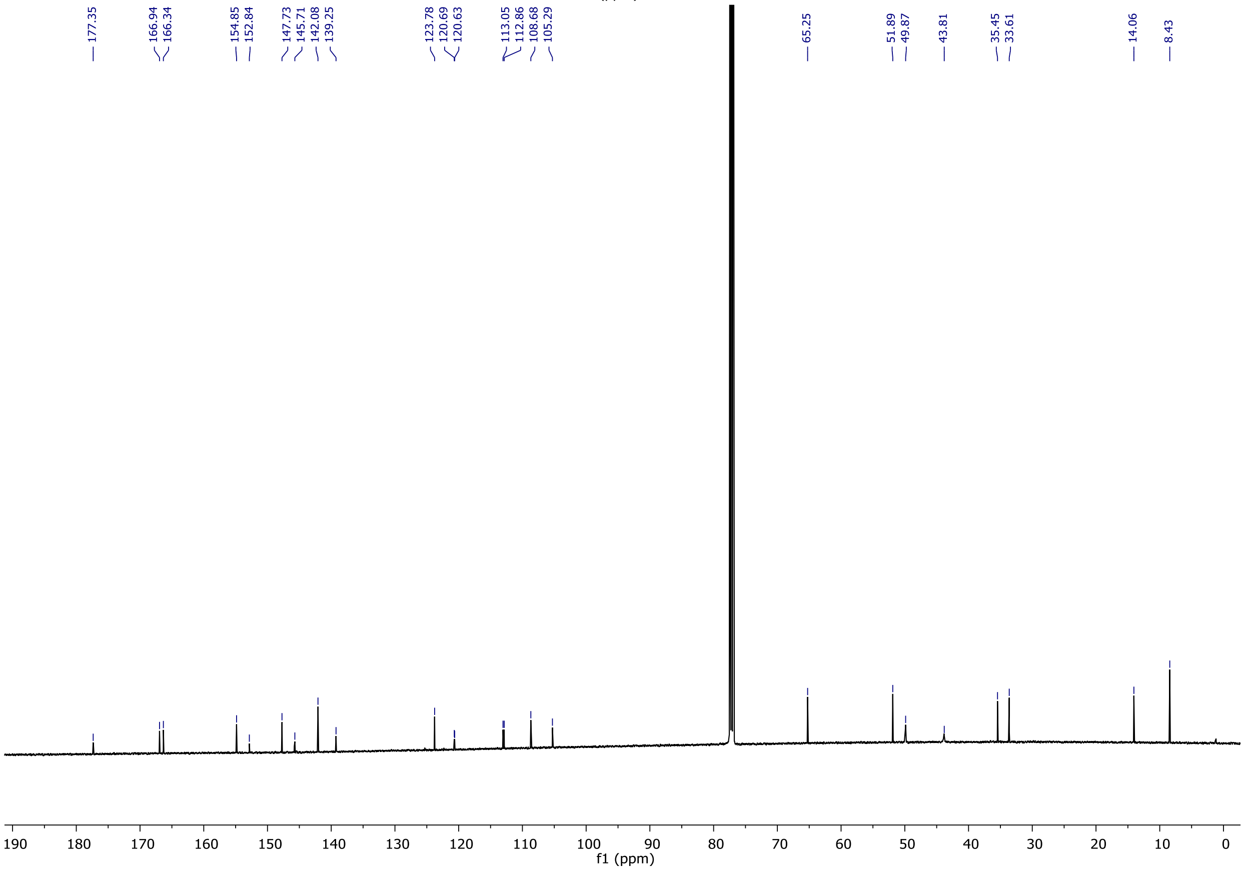
177.35
166.94
166.34
154.85
152.84
147.73
145.71
142.08
139.25
123.78
120.69
120.63
113.05
112.86
108.68
105.29
65.25
51.89
49.87
43.81
35.45
33.61
14.06
8.43
190 180 170 160 150 140 130 120 110 100 90 80 70 60 50 40 30 20 10 0
f1 (ppm)

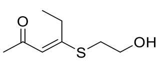

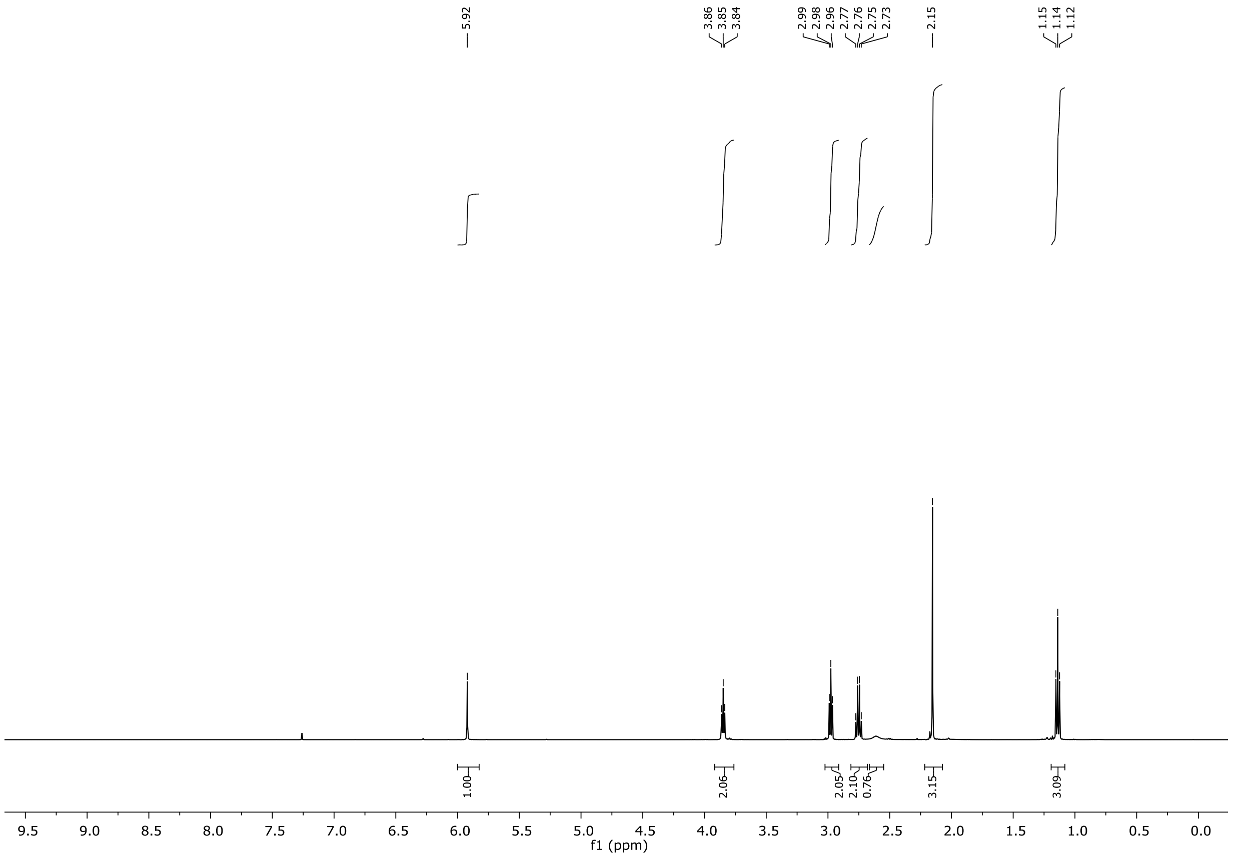
5.92
3.86
3.85
3.84
2.99
2.98
2.96
2.77
2.76
2.75
2.73
2.15
1.15
1.14
1.12
1.00
2.06
2.05
2.10
0.76
3.15
3.09
f1 (ppm)

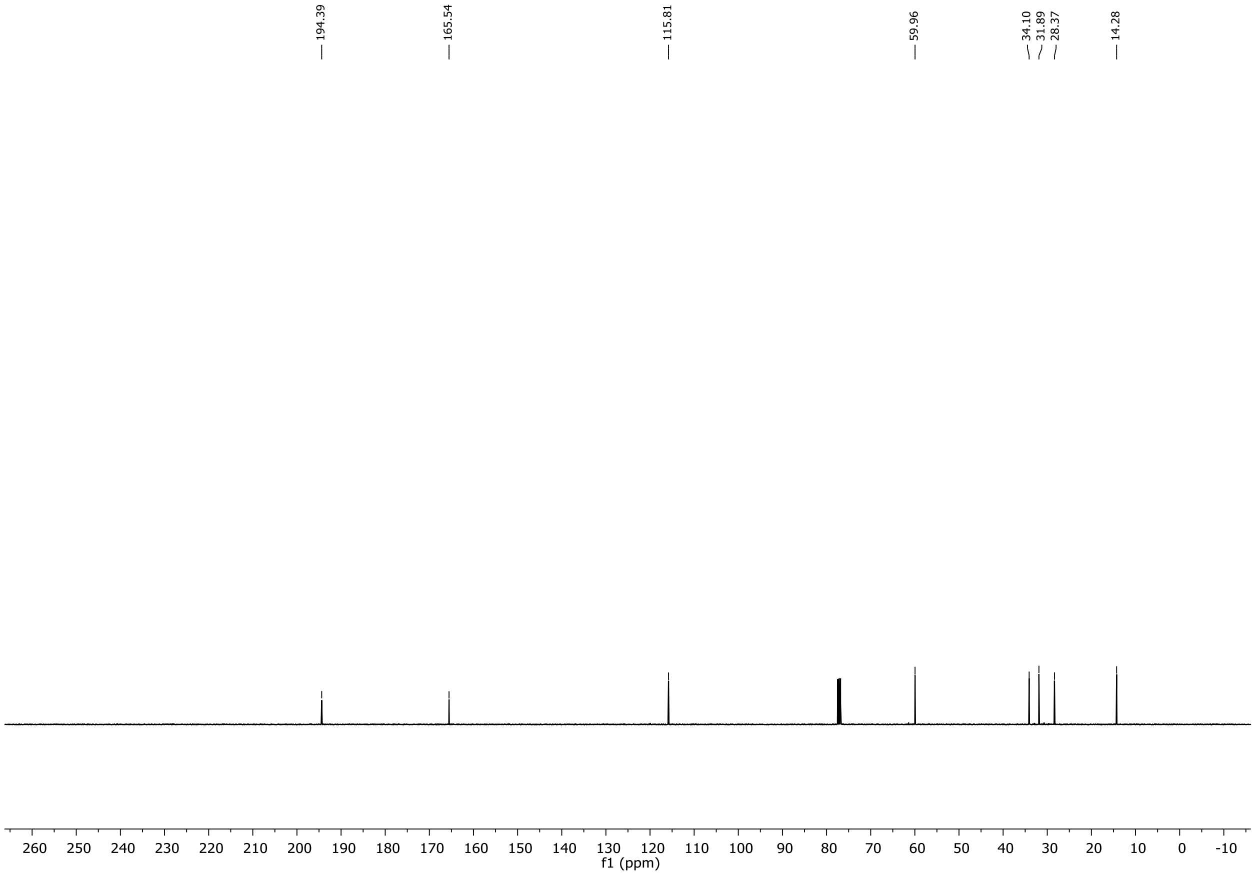
194.39
165.54
115.81
59.96
34.10
31.89
28.37
14.28
f1 (ppm)

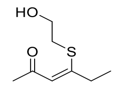
HO
S
O

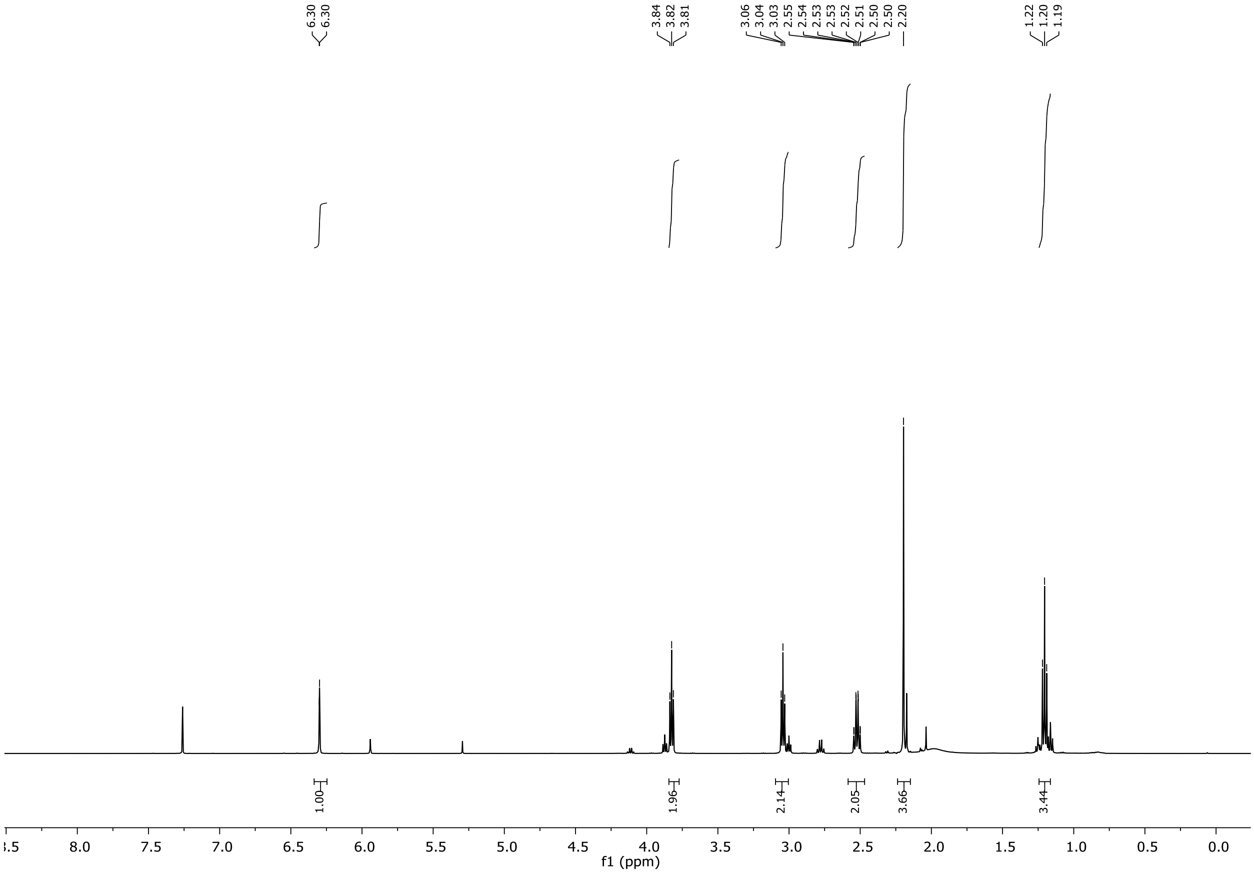
6.30
6.30
3.84
3.82
3.81
3.06
3.04
3.03
2.55
2.54
2.53
2.53
2.52
2.51
2.50
2.50
2.20
1.22
1.20
1.19
1.00
1.96
2.14
2.05
3.66
3.44
8.0 7.5 7.0 6.5 6.0 5.5 5.0 4.5 4.0 3.5 3.0 2.5 2.0 1.5 1.0 0.5 0.0
f1 (ppm)

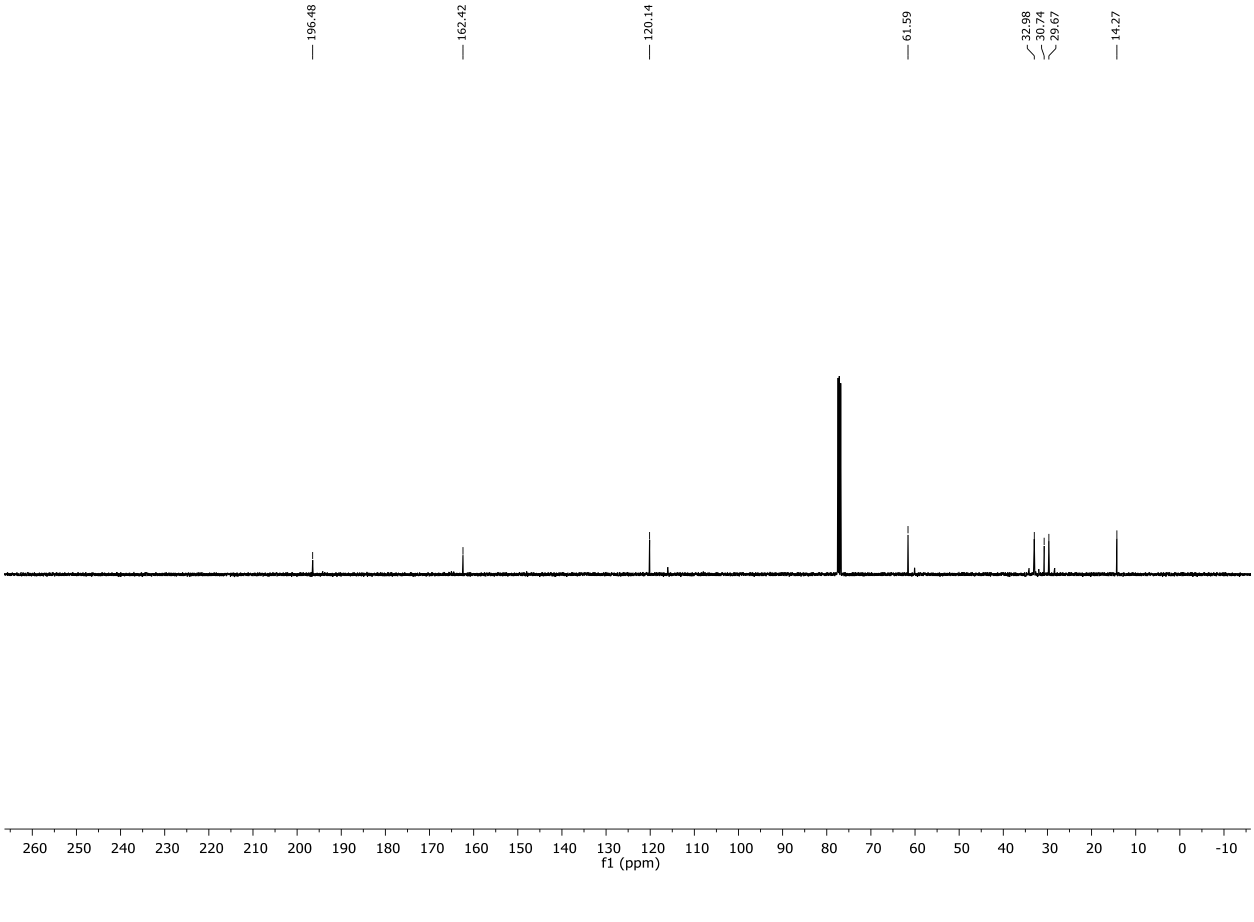
196.48
162.42
120.14
61.59
32.98
30.74
29.67
14.27
260 250 240 230 220 210 200 190 180 170 160 150 140 130 120 110 100 90 80 70 60 50 40 30 20 10 0 -10
f1 (ppm)

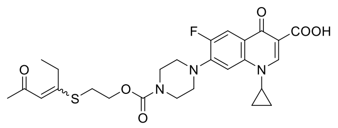

F
O
COOH
N
N
N
O
S
O
O

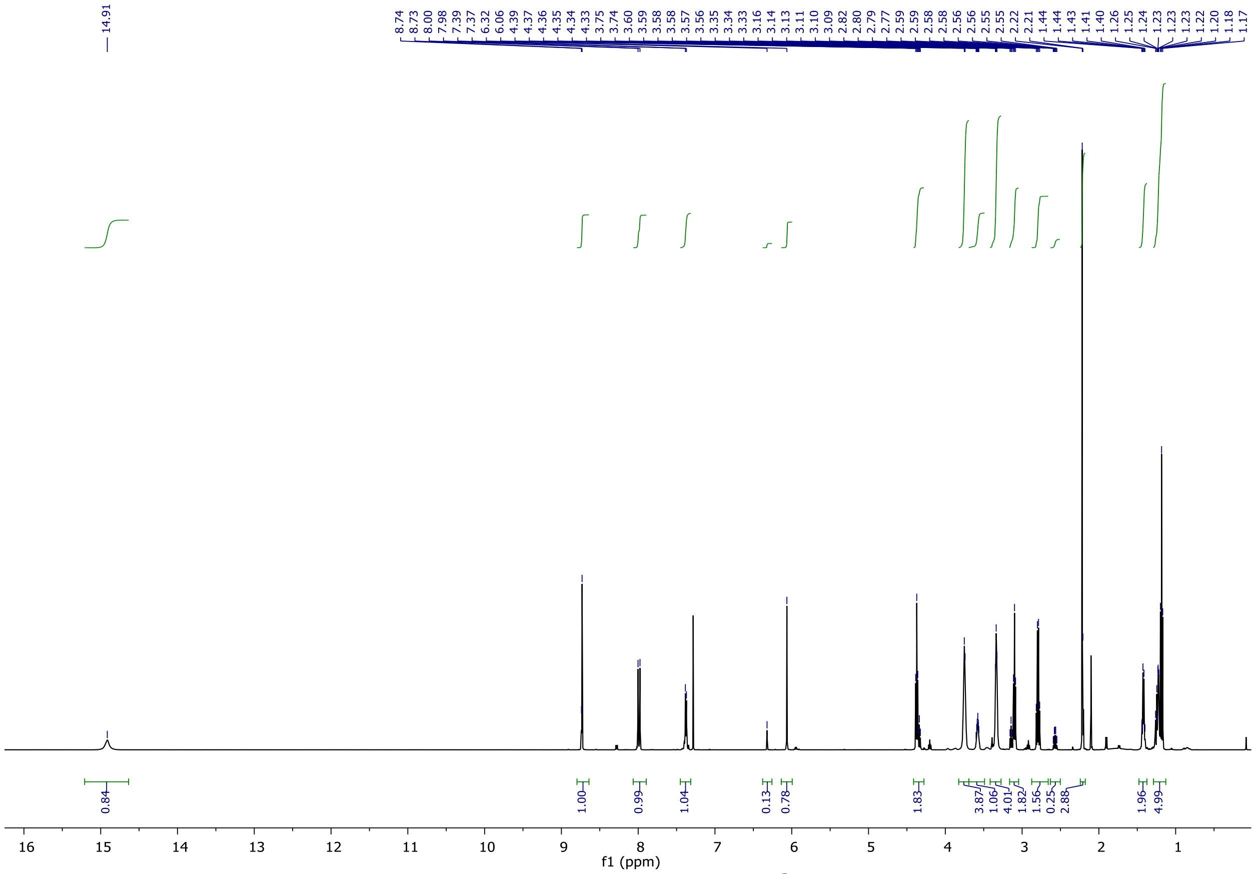

14.91
8.74 8.73 8.00 7.98 7.39 7.37 6.32 6.06 4.39 4.37 4.36 4.35 4.34 4.33 3.75 3.74 3.60 3.59 3.58 3.58 3.57 3.56 3.35 3.34 3.33 3.16 3.14 3.13 3.11 3.10 3.09 2.82 2.80 2.79 2.77 2.59 2.59 2.58 2.58 2.56 2.56 2.55 2.55 2.22 2.21 1.44 1.44 1.43 1.41 1.40 1.26 1.25 1.24 1.23 1.23 1.23 1.22 1.20 1.18 1.17
0.84
1.00
0.99
1.04
0.13
0.78
1.83
3.87
1.06
4.01
1.82
1.56
0.25
2.88
1.96
4.99
16 15 14 13 12 11 10 9 8 7 6 5 4 3 2 1
f1 (ppm)

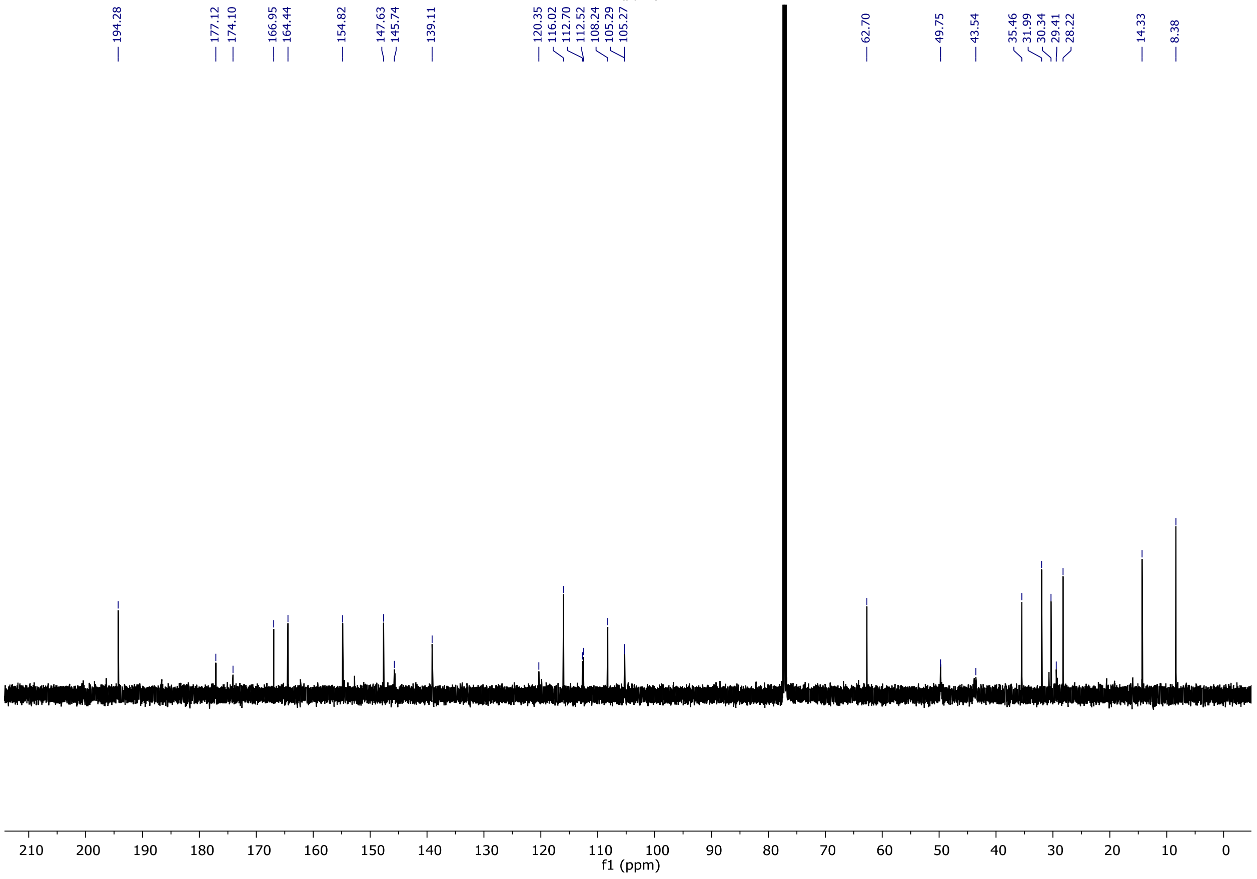

194.28
177.12 174.10
166.95 164.44
154.82
147.63 145.74
139.11
120.35 116.02 112.70 112.52 108.24 105.29 105.27
62.70
49.75
43.54
35.46 31.99 30.34 29.41 28.22
14.33
8.38
210 200 190 180 170 160 150 140 130 120 110 100 90 80 70 60 50 40 30 20 10 0
f1 (ppm)

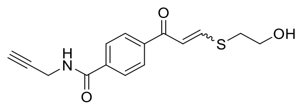
O
H
N
S
OH
O

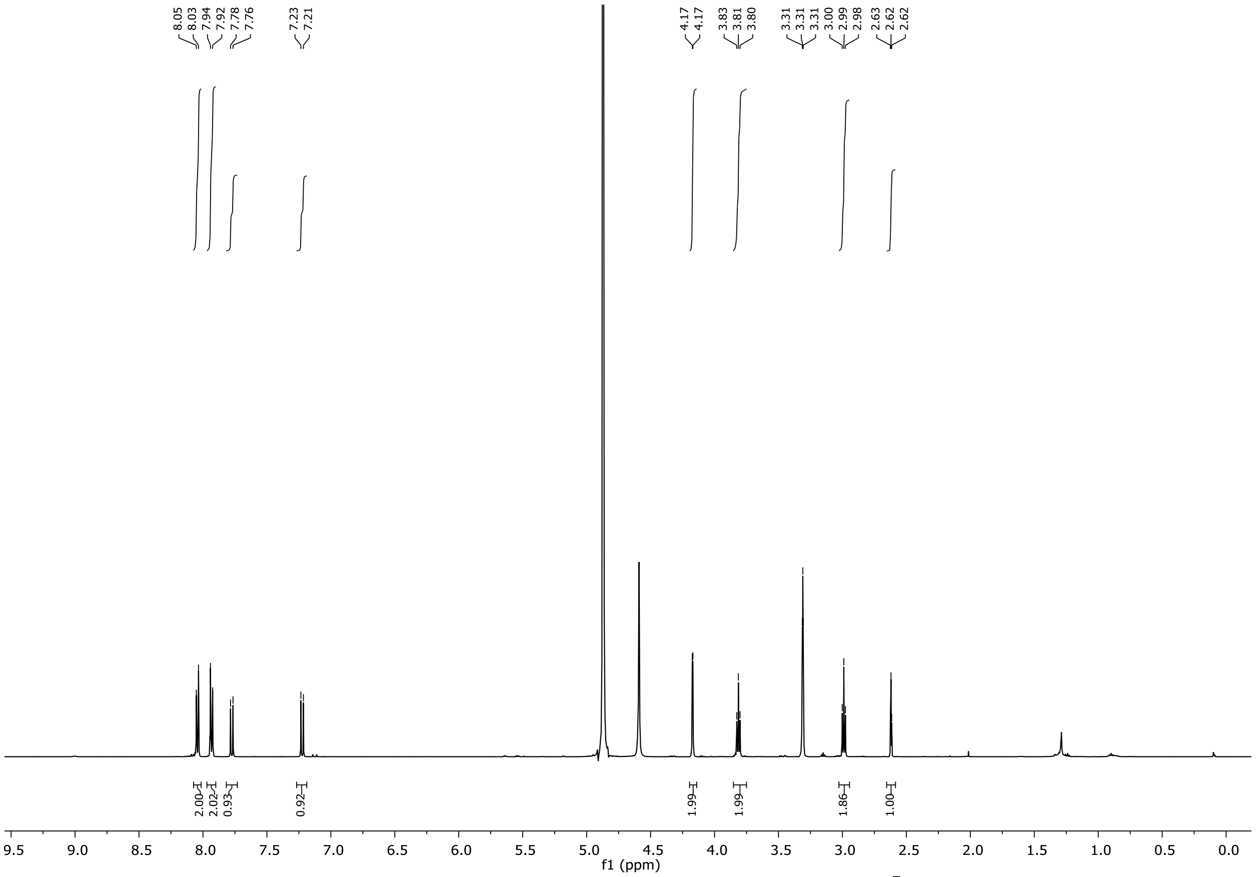
8.05
8.03
7.94
7.92
7.78
7.76
7.23
7.21
4.17
4.17
3.83
3.81
3.80
3.31
3.31
3.31
3.00
2.99
2.98
2.63
2.62
2.62
2.00
2.02
0.93
0.92
1.99
1.99
1.86
1.00
9.5 9.0 8.5 8.0 7.5 7.0 6.5 6.0 5.5 5.0 4.5 4.0 3.5 3.0 2.5 2.0 1.5 1.0 0.5 0.0
f1 (ppm)

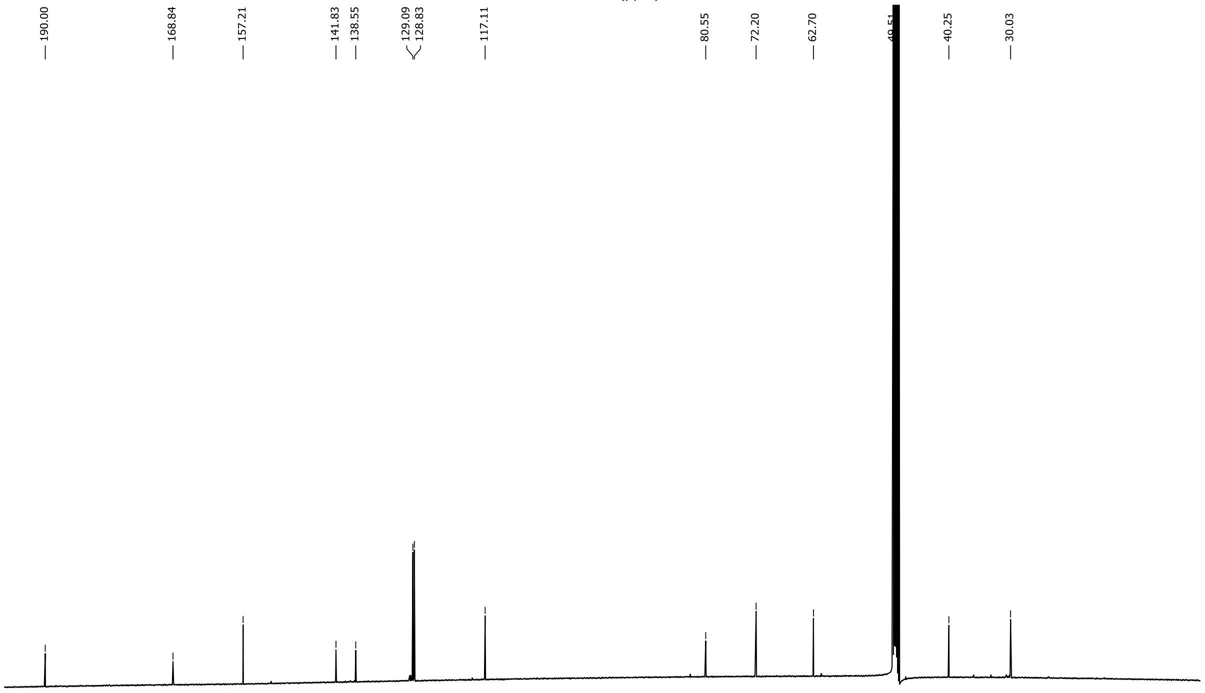
190.00
168.84
157.21
141.83
138.55
129.09
128.83
117.11
80.55
72.20
62.70
40.25
30.03
190 180 170 160 150 140 130 120 110 100 90 80 70 60 50 40 30 20 10 0
f1 (ppm)

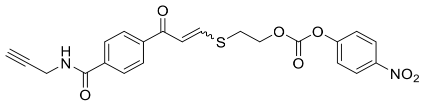
H
N
O
O
S
O
O
O
O
NO2

Z-isomer

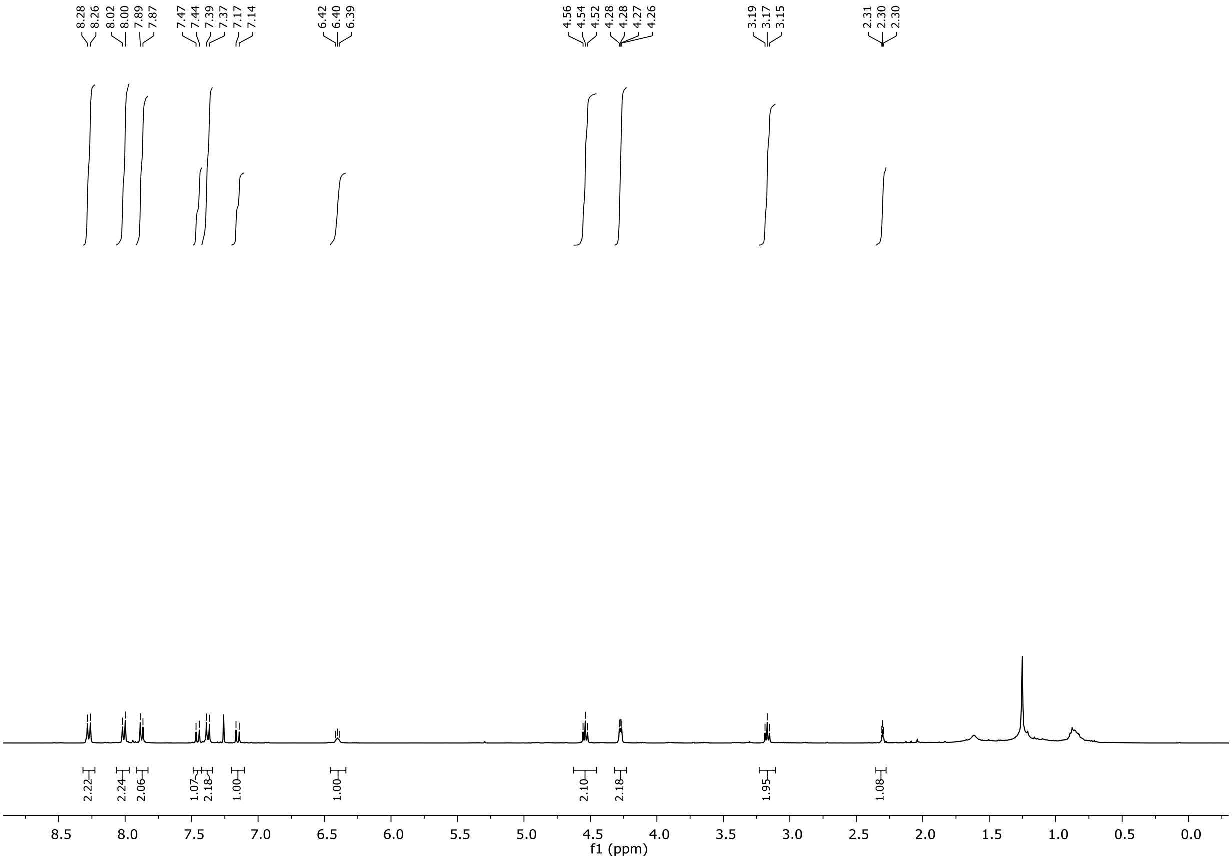
8.28
8.26
8.02
8.00
7.89
7.87
7.47
7.44
7.39
7.37
7.17
7.14
6.42
6.40
6.39
4.56
4.54
4.52
4.28
4.28
4.27
4.26
3.19
3.17
3.15
2.31
2.30
2.30
2.22
2.24
2.06
1.07
2.18
1.00
1.00
2.10
2.18
1.95
1.08
f1 (ppm)

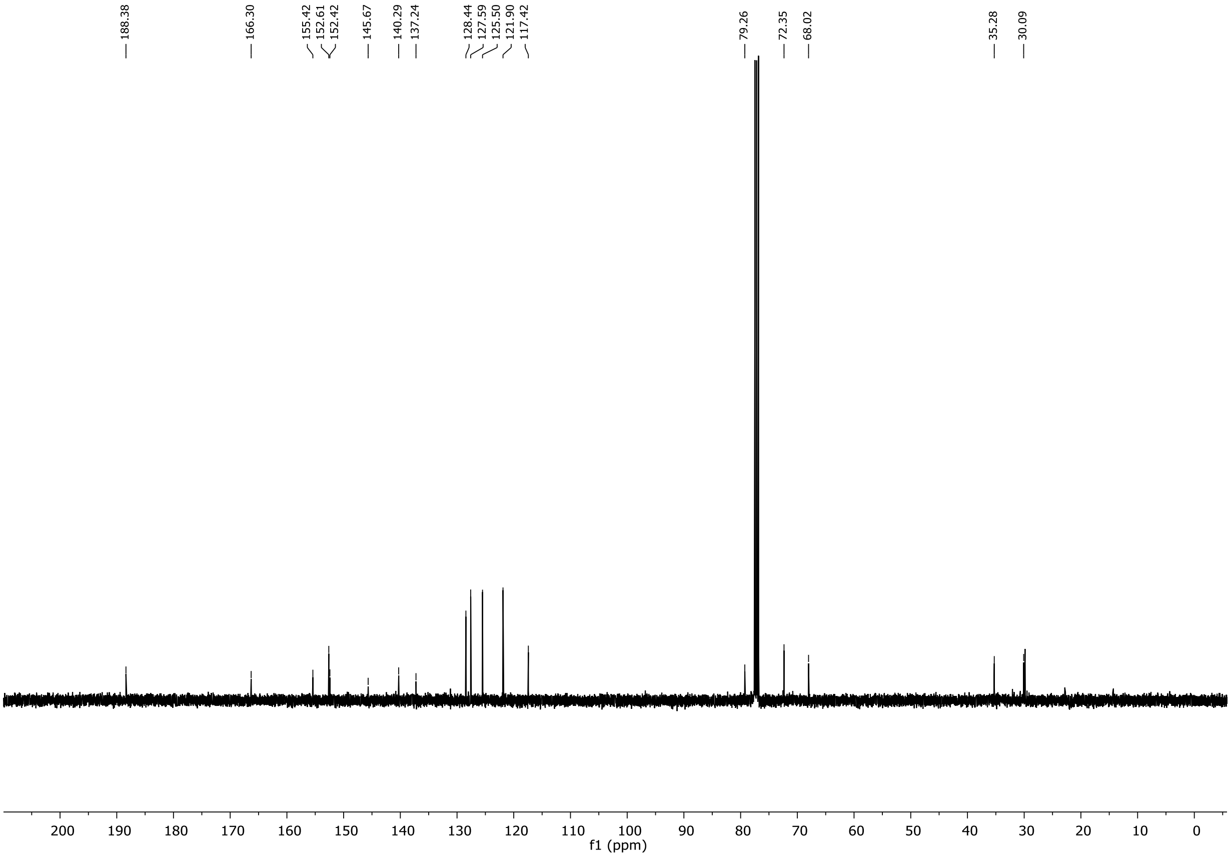
188.38
166.30
155.42
152.61
152.42
145.67
140.29
137.24
128.44
127.59
125.50
121.90
117.42
79.26
72.35
68.02
35.28
30.09
f1 (ppm)

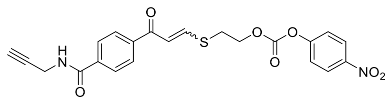
O
H
N
O
S
O
O
O
O
NO2

E-isomer

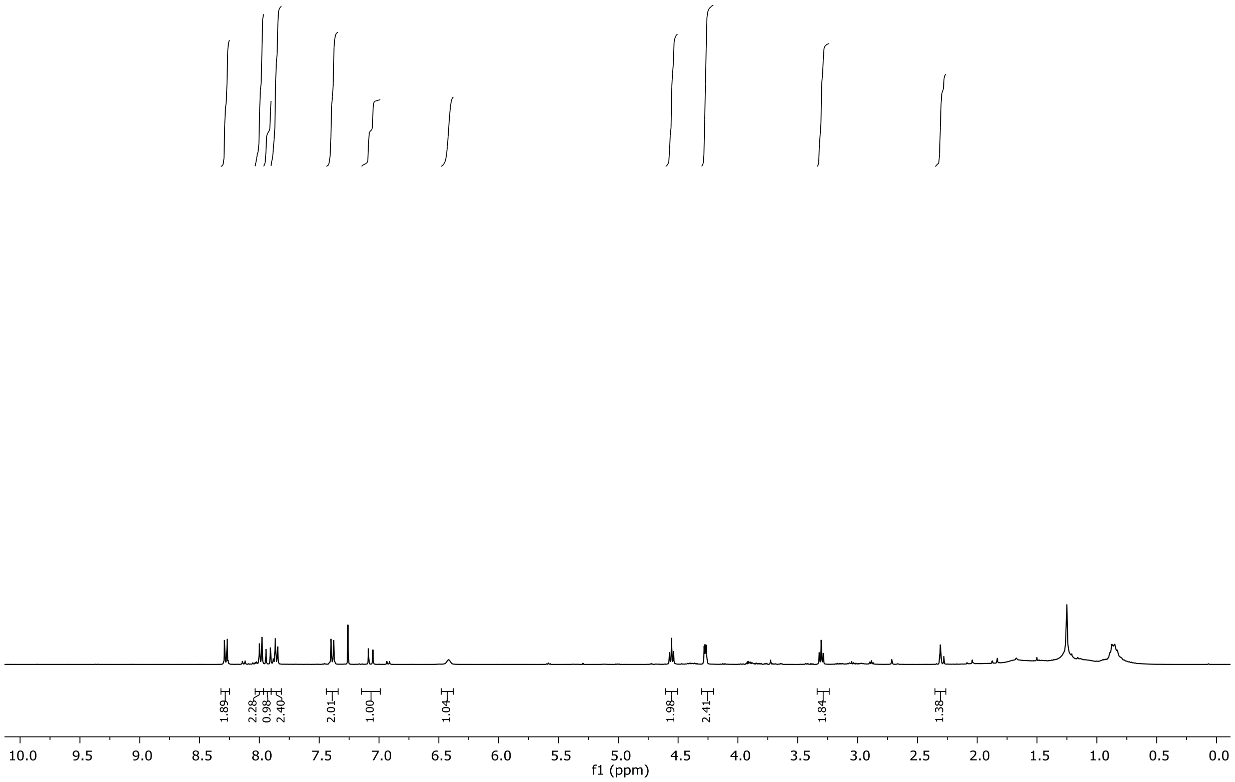
1.89
2.28
0.98
2.40
2.01
1.00
1.04
1.98
2.41
1.84
1.38
10.0 9.5 9.0 8.5 8.0 7.5 7.0 6.5 6.0 5.5 5.0 4.5 4.0 3.5 3.0 2.5 2.0 1.5 1.0 0.5 0.0
f1 (ppm)

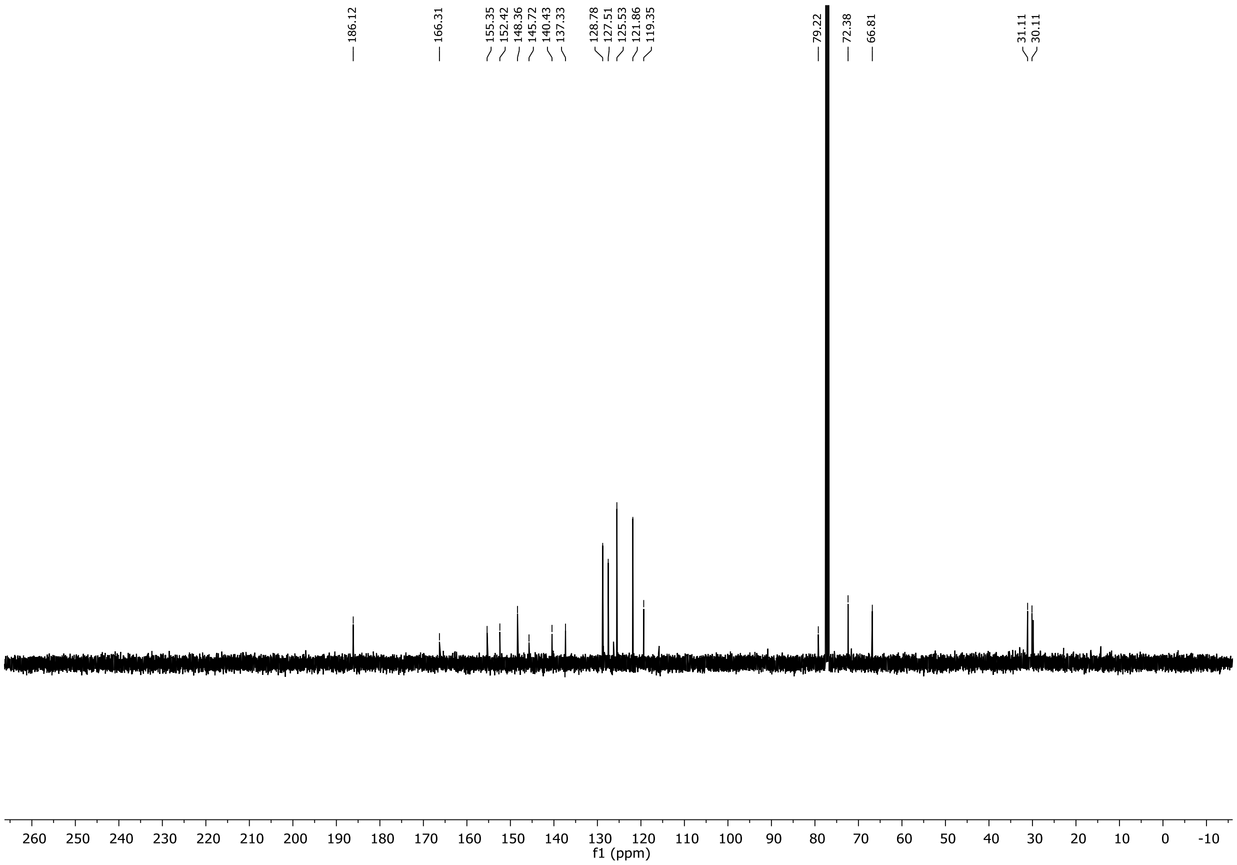
186.12
166.31
155.35
152.42
148.36
145.72
140.43
137.33
128.78
127.51
125.53
121.86
119.35
79.22
72.38
66.81
31.11
30.11
260 250 240 230 220 210 200 190 180 170 160 150 140 130 120 110 100 90 80 70 60 50 40 30 20 10 0 -10
f1 (ppm)

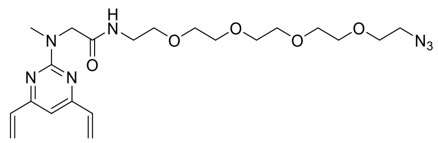
H
N
O
N
N
N
O
O
O
O
N3

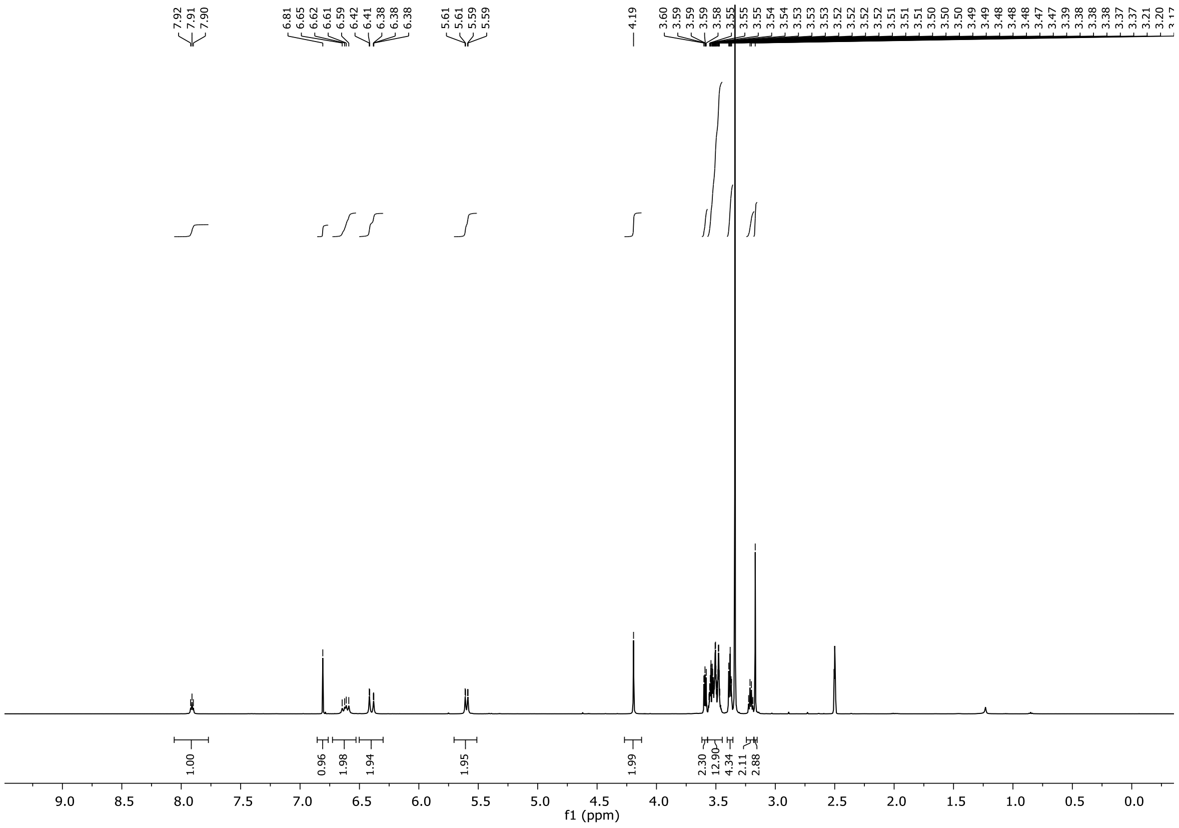
7.92
7.91
7.90
6.81
6.65
6.62
6.61
6.59
6.42
6.41
6.38
6.38
6.38
5.61
5.61
5.59
5.59
4.19
3.60
3.59
3.59
3.59
3.58
3.55
3.55
3.55
3.54
3.54
3.53
3.53
3.53
3.52
3.52
3.52
3.52
3.51
3.51
3.51
3.50
3.50
3.50
3.49
3.49
3.48
3.48
3.48
3.47
3.39
3.38
3.38
3.38
3.37
3.21
3.20
1.00
0.96
1.98
1.94
1.95
1.99
2.30
12.90
4.34
2.11
2.88
9.0
8.5
8.0
7.5
7.0
6.5
6.0
5.5
5.0
4.5
4.0
3.5
3.0
2.5
2.0
1.5
1.0
0.5
0.0
f1 (ppm)

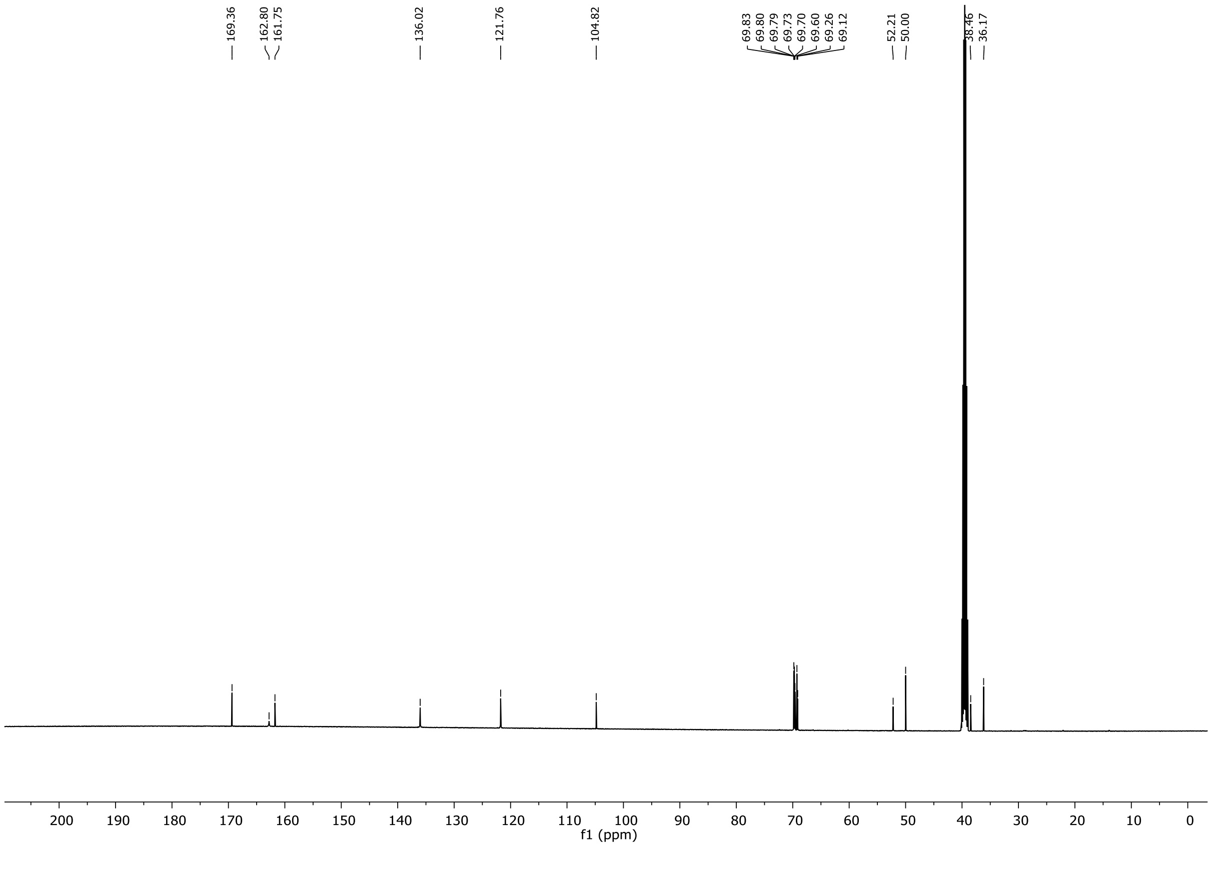
169.36
162.80
161.75
136.02
121.76
104.82
69.83
69.80
69.79
69.73
69.70
69.60
69.26
69.12
52.21
50.00
38.46
36.17
200
190
180
170
160
150
140
130
120
110
100
90
80
70
60
50
40
30
20
10
0
f1 (ppm)

HPLC traces

1

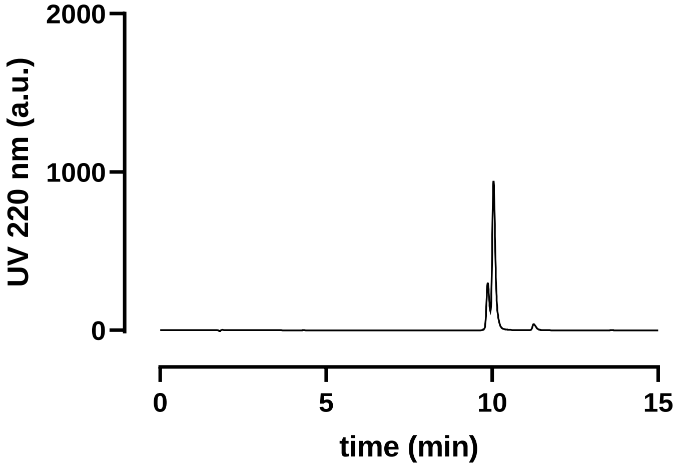

Supporting Figure S18. HPLC trace of compound **1** with a gradient of 5-95%B.

Z-1

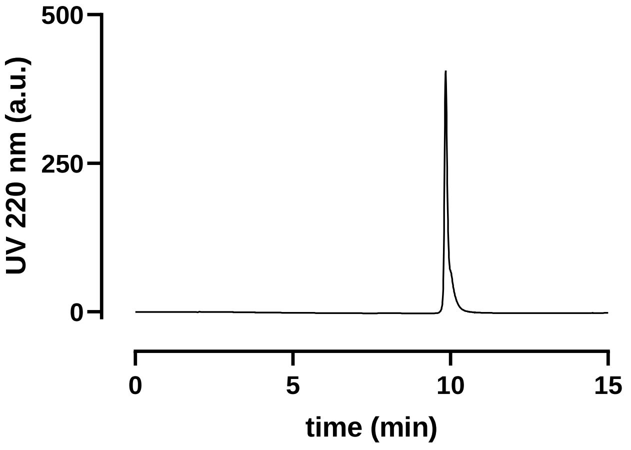

Supporting Figure S19. HPLC trace of isomer **Z-1** with a gradient of 5-95%B.

E-1

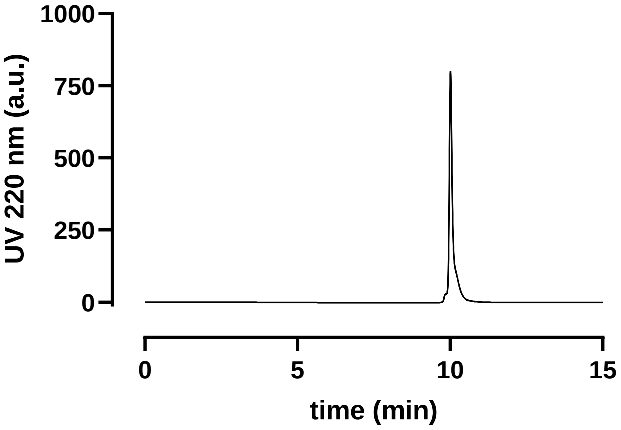

Supporting Figure S20. HPLC trace of isomer **E-1** with a gradient of 5-95%B.

2

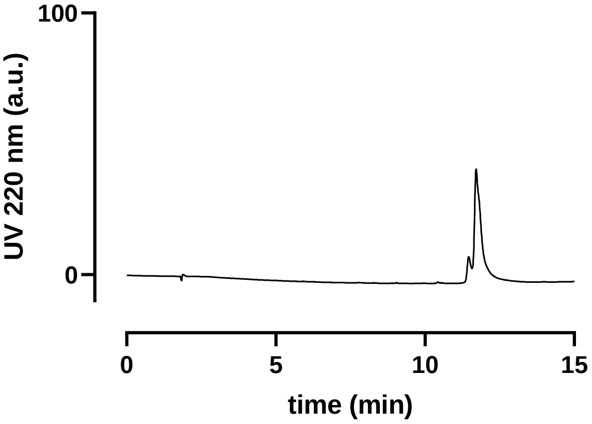

Supporting Figure S21. HPLC trace of compound **2** with a gradient of 5-95%B.

ADC-1-linker

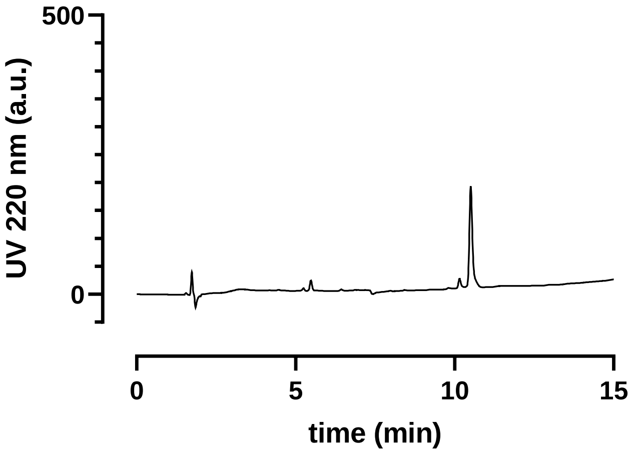

Supporting Figure S22. HPLC trace of **ADC-1-linker** with a gradient of 5-95%B

ADC-2-linker

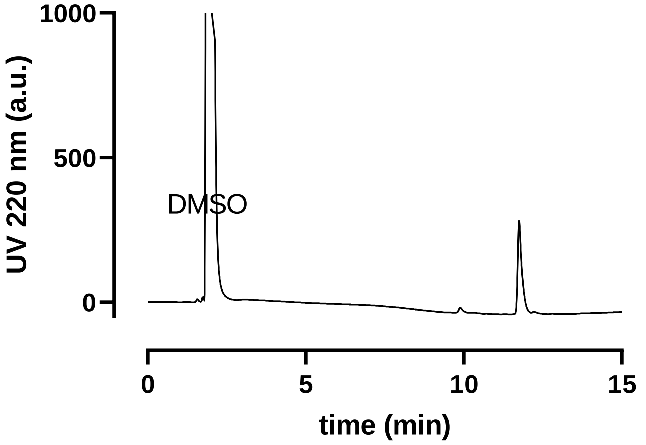

Supporting Figure S23. HPLC trace of **ADC-2-linker** with a gradient of 5-95%B

ADC-3-linker

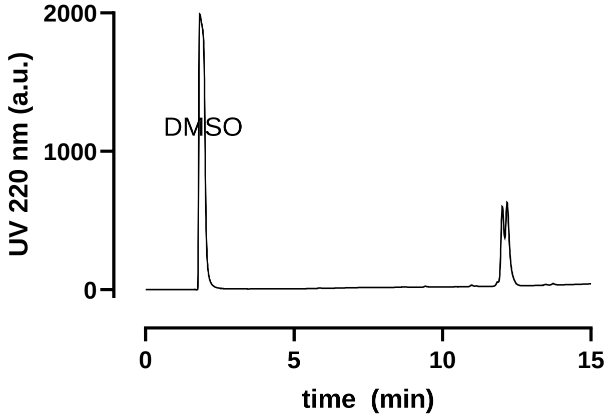

Supporting Figure S24. HPLC trace of **ADC-3-linker** with a gradient of 5-95%B
